# Supplementary material for: RAGE is a key regulator of ductular reaction-mediated fibrosis during cholestasis
Source: EMBO Rep. 2025 Jan 2;26(3):880–907. doi: 10.1038/s44319-024-00356-7 (PMC11811172; doi:10.1038/s44319-024-00356-7)
Supplement: Supplementary file 1 — Appendix [file 44319_2024_356_MOESM1_ESM.pdf]

RAGE is a key regulator of ductular reaction-mediated fibrosis during cholestasis

Appendix Figures and Tables

Table of Contents

Appendix Figure S1. *Rage* is enriched in BECs in CDE-challenged mice. 2

Appendix Figure S2. Differentially expressed genes between *Rage* control and KO BECs in the enriched hepatic fibrosis pathways. 3

Appendix Figure S3. BECs activate stellate cells in *Rage*-dependent manner upon chronic injury. 4

Appendix Figure S4. Confirmation of *Rage* deletion in BEC. 5

Appendix Figure S5. Mass spectrometry analysis of the differential secretory proteomic profile between the conditioned medium of *Rage* WT and KO BECs. 6

Appendix Figure S6. Differential expression of mesenchymal markers in *Rage* control and KO BECs. 7

Appendix Table S1. PCR primers used for genotyping. 8

Appendix Table S2. Mouse primers used for qPCR analysis. 9

Appendix Table S3. KEGG enriched pathways between *Rage* control and *Rage* knockout BECs. 10

Appendix Table S4. REACTOME enriched pathways between *Rage* control and *Rage* knockout BECs. 11

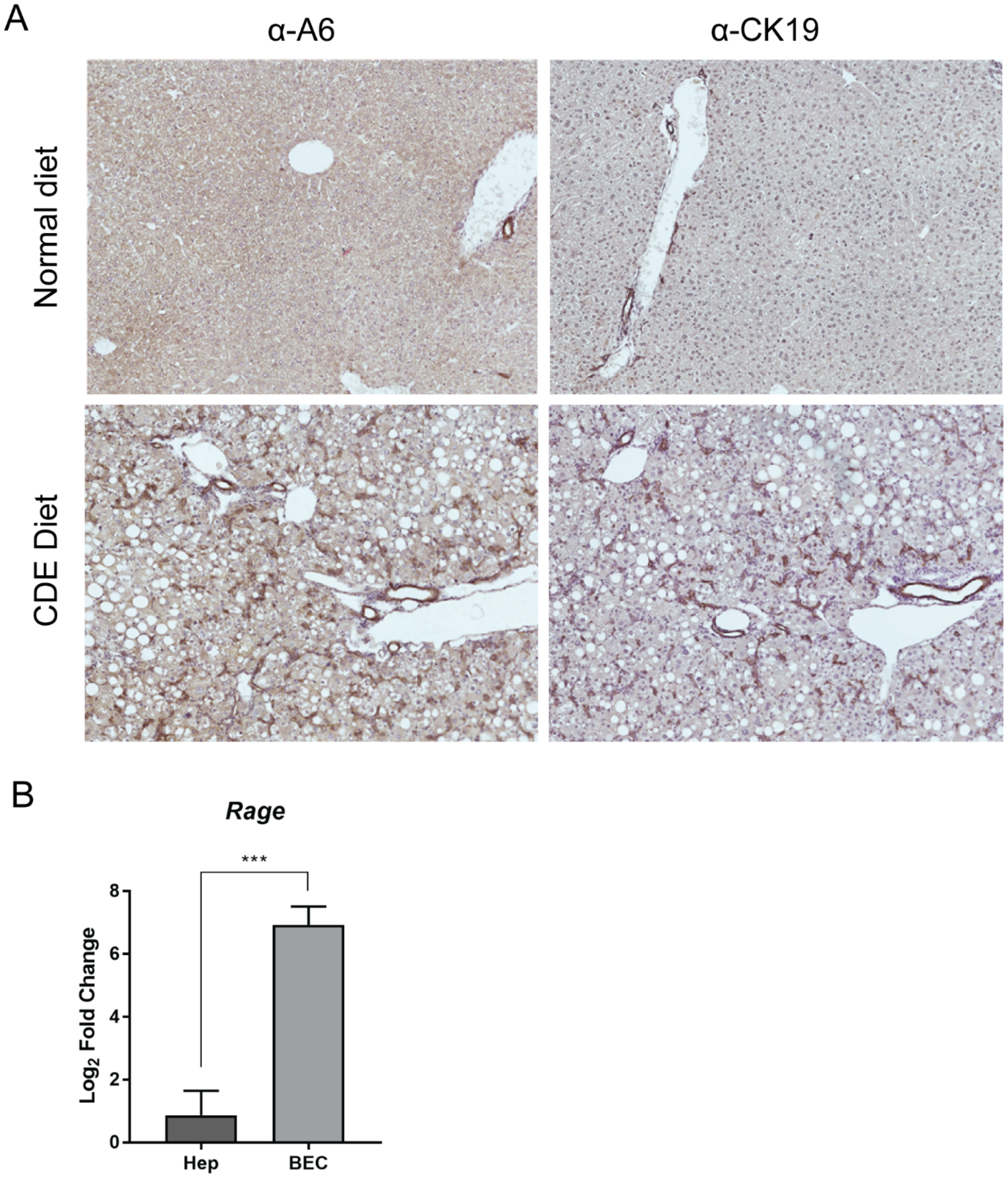

Appendix Figure S1. ***Rage*** **is enriched in BECs in CDE-challenged mice. (A)** IHC staining of BEC-specific markers A6 and CK19 in C57BL/6 wildtype mice fed with normal diet or CDE diet for three weeks. **(B)** mRNA expression of *Rage* in hepatocytes (Hep) and BECs. Data was shown as mean ± s.d. of n=3 independent biological replicates. Two-tailed t-test was used for statistical comparisons (***p < 0.001).

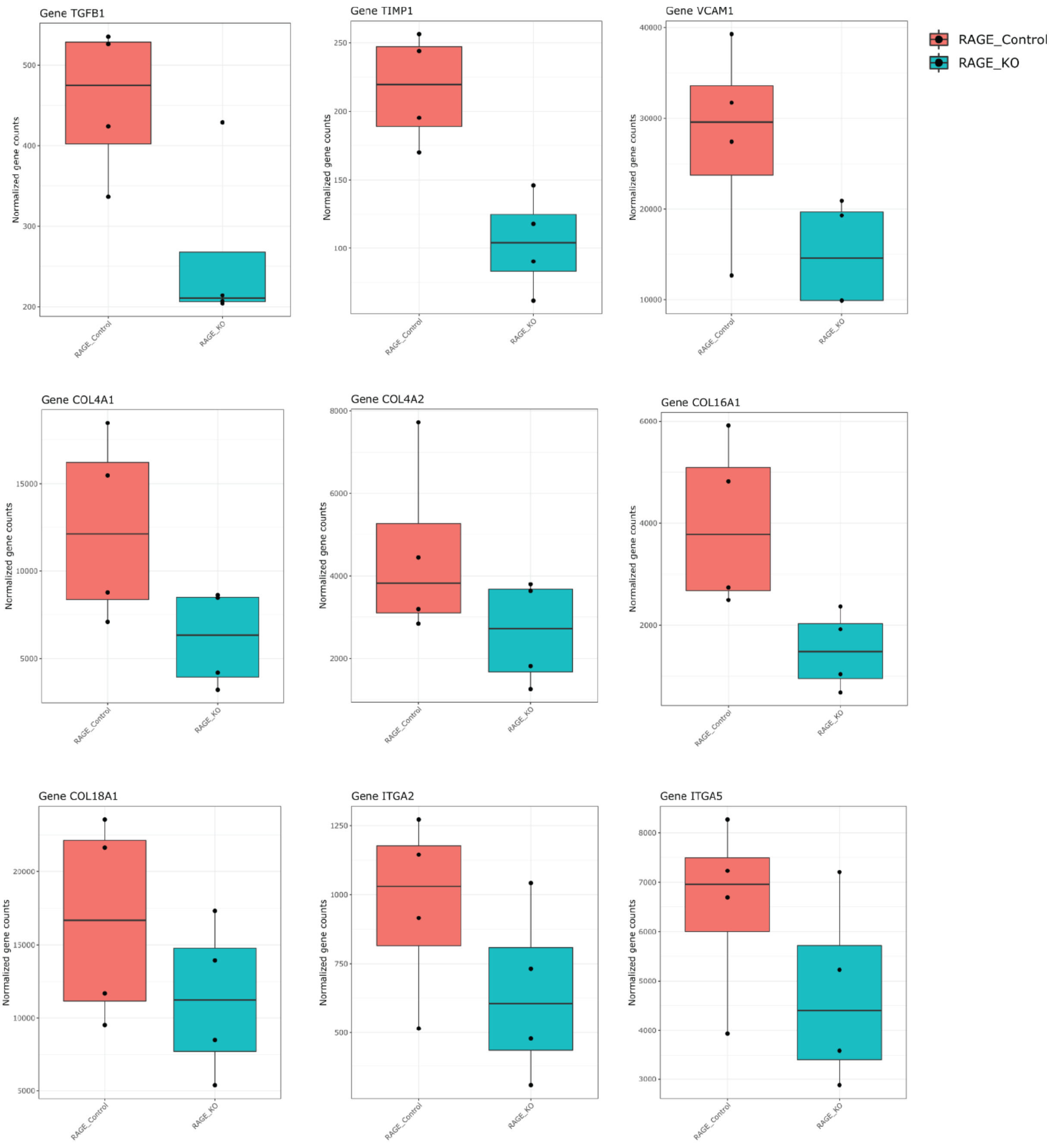

Appendix Figure S2. Differentially expressed genes between *Rage* control and KO BECs in the enriched hepatic fibrosis pathways. Normalized gene counts of the classical fibrotic mediators and markers (*Tgfb1*, *Timp1* and *Vcam1*), collagen of the ECM (*Col4a1*, *Col4a2*, *Col16A1* and *Col18a1*) and cell surface adhesion and signaling integrin (*Itga2*, *Itga5*) in the primary *Rage* control and KO BECs isolated from CDE-challenged mice. n=4 animals (biological replicates) per group.

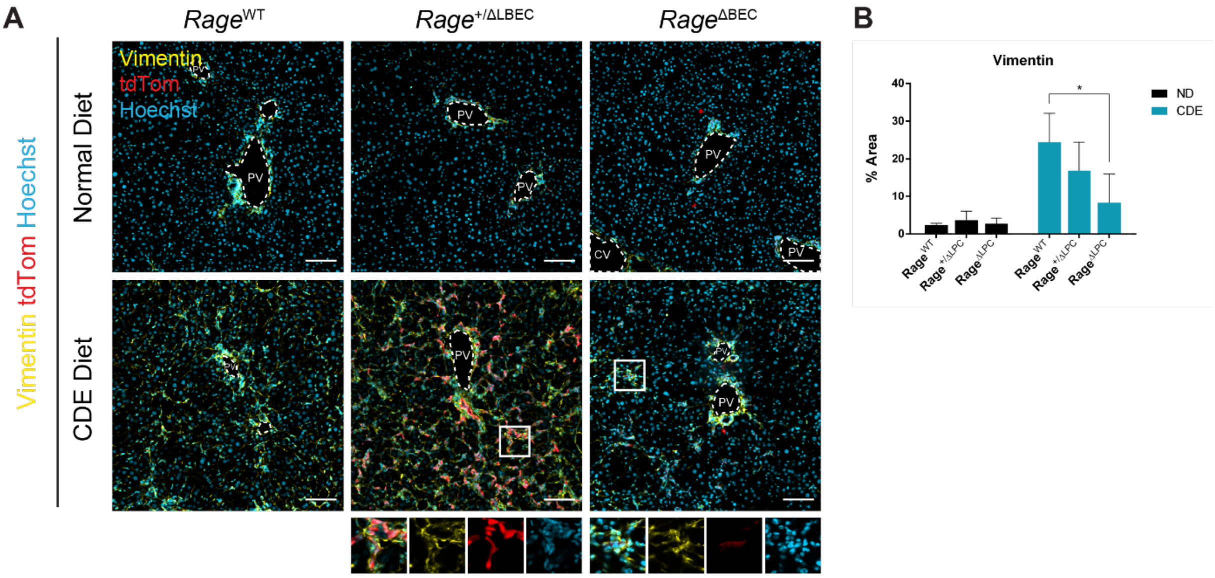

Appendix Figure S3. BECs activate stellate cells in *Rage*-dependent manner upon chronic injury. (A) Representative images showing IF staining of HSC marker Vimentin to assess the abundance of stellate cells in $Rage^{WT}$, $Rage^{+/\Delta BEC}$ and $Rage^{\Delta BEC}$ mice fed with normal diet (ND) or CDE diet. Scale bar = 100 µm. PV, portal vein. **(B)** Quantification of percent area of Vimentin staining (yellow) in A. Data was shown as mean ± s.d. of n=6 animals (biological replicates) per group. Two-way ANOVA with Turkey's multiple comparisons test was used for statistical comparison (*$p < 0.05$).

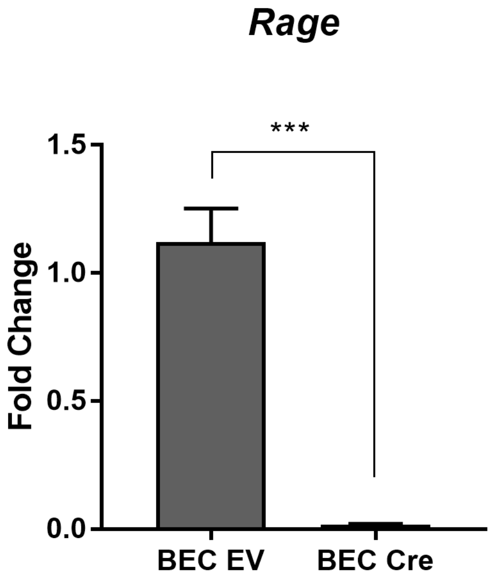

Appendix Figure S4. Confirmation of *Rage* deletion in BEC. qPCR analysis was performed on *Rage*$^{fl/fl}$ BEC transfected with pMXpie plasmid that carries an empty vector (EV) or Cre recombinase (Cre). *Rage* was successfully deleted in BECs transfected with pMXpie-Cre. Data was shown as mean ± s.d. of n=3 technical replicates. Two-tailed t-test was used for statistical comparison (***$p < 0.001$).

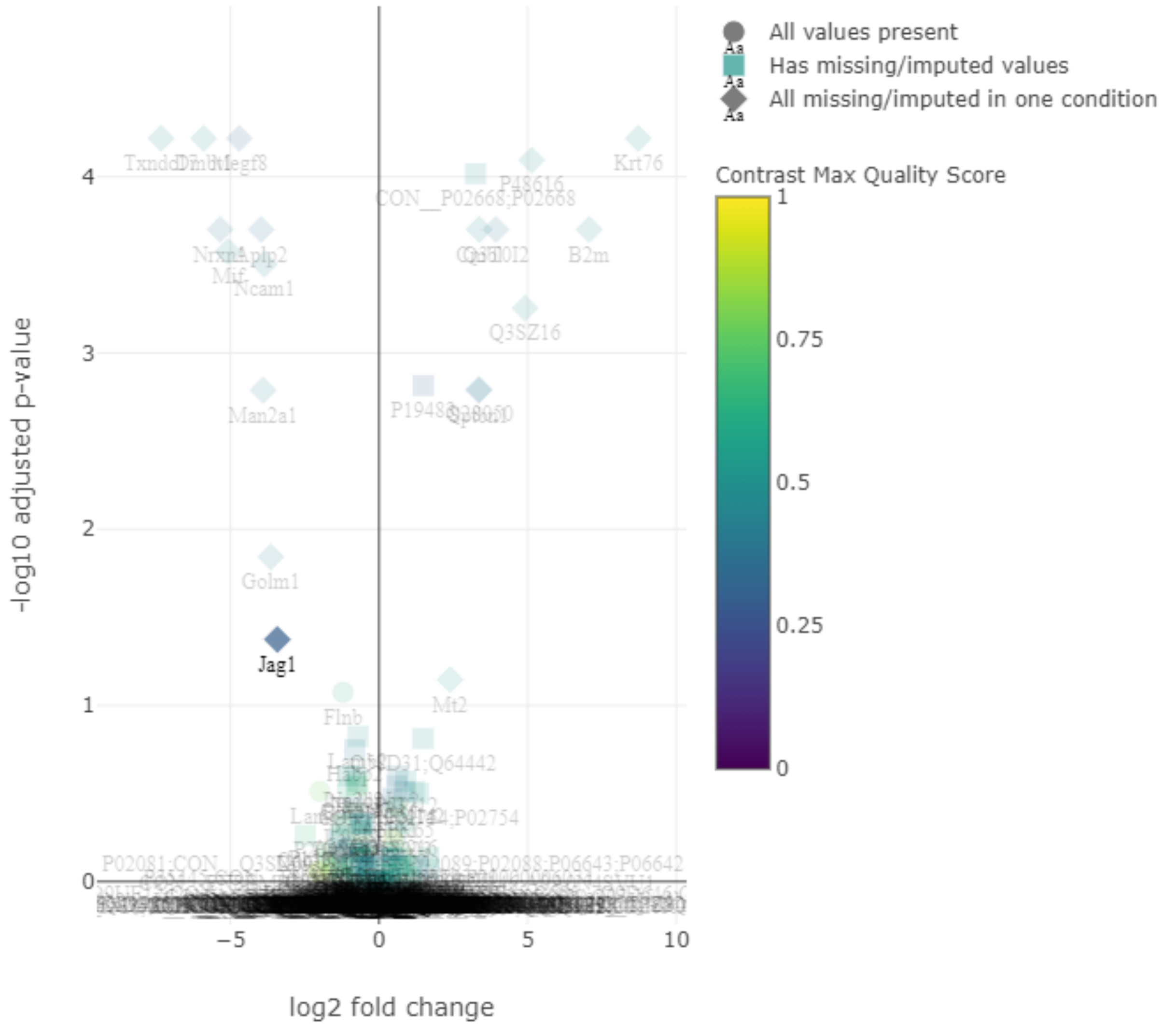

Appendix Figure S5. Mass spectrometry analysis of the differential secretory proteomic profile between the conditioned medium of *Rage* WT and KO BECs. Jag1 is one of the secretory factors showing differential abundance

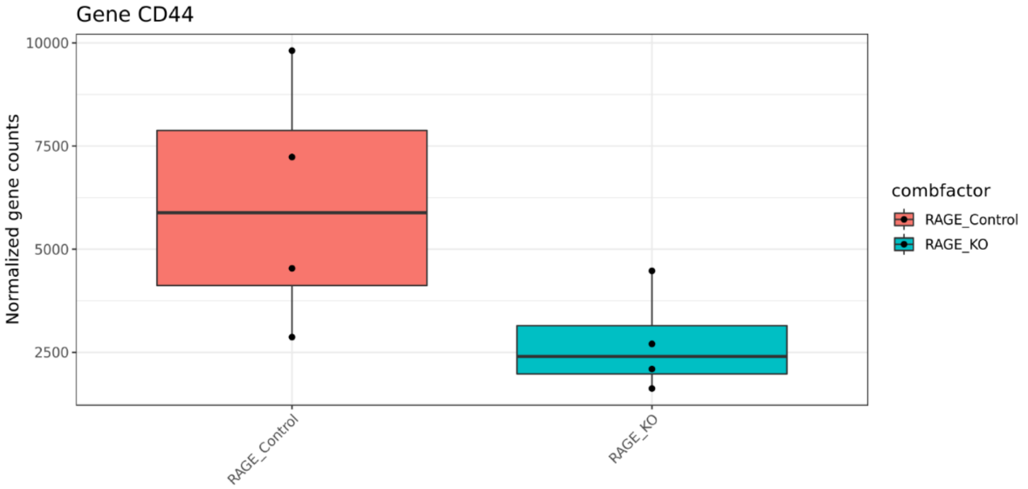

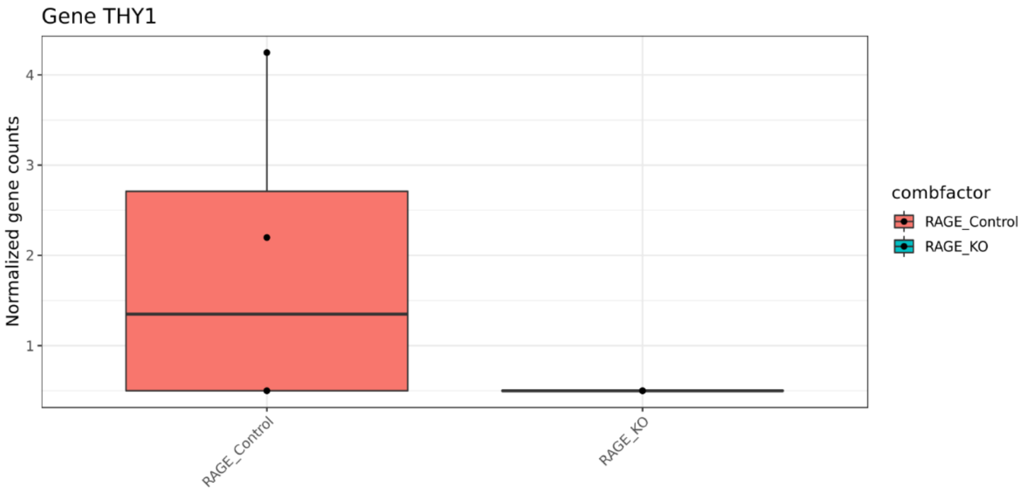

Appendix Figure S6. Differential expression of mesenchymal markers in *Rage* control and KO BECs. Normalized gene counts of the mesenchymal markers *Cd44* and *Thy1* in the primary *Rage* control and KO BECs isolated from CDE-challenged mice. n=4 animals (biological replicates) per group.

Appendix Tables

Appendix Table S1. PCR primers used for genotyping.

**Gene names**	**Strands**	**Sequences (5' to 3')**	**Amplicon sizes (bp)**
Rage exon 5/7	Forward	AGCTGGCACTTAGATGGGAAACTT	500
	Reverse	TGGGCAGAGATGGCACAGGT	
Rage exon 6/8	Forward	CCCCACCCAAGGAGGAAC	950
	Reverse	TCAGGGAGGAGCAGCACAG	
eGFP	Forward	CAGGAGCGCACCATCTTCTT	300
	Reverse	TGGGGGTGTTCTGCTGGTAG	
CreERT2 control	Forward	CAATGGTAGGCTCACTCTGG	300
	Reverse	AACACACACTGGCAGGACTG	
CreERT2 347/349	Forward	CCTGGAAAATGCTTCTGT	400
	Reverse	CAGGGTGTTATAAGCAATCCC	
tdTomato control	Forward	AAGGGAGCTGCAGTGGAGTA	200
	Reverse	CCGAAAATCTGTGGGAAGTC	
tdTomato	Forward	CTGTTCCTGTACGGCATGG	300
	Reverse	GGCATTAAAGCAGCGTATCC	

Appendix Table S2. Mouse primers used for qPCR analysis.

**Real time PCR mouse primers**		
**Primer**	**Forward sequence (5’ -> 3’)**	**Reverse sequence (5’ -> 3’)**
Acta2	GTCCCAGACATCAGGGAGTAA	TCGGATACTTCAGCGTCAGGA
Col1a1	GCTCCTCTTAGGGGCCACT	CCACGTCTCACCATTGGGG
Gapdh	TGAAGCAGGCATCTGAGGG	CGAAGGTGGAAGAGTGGGA
Hprt	CTGGTTAAGCAGTACAGCCC	CAAAAGTCTGGGGACGCAGC
Jag1	CCTCGGGTCAGTTTGAGCTG	CCTTGAGGCACACTTTGAAGTA
Rage	ACAGGCGAGGGAAGGAGGTC	TTTGCCATCGGGAATCAGAAG

Appendix Table S3. KEGG enriched pathways between *Rage* control and *Rage* knockout BECs.

KEGG enriched pathways					
**Pathway name**	**Pathway code**	**DE genes**	**Pathway size**	**Fraction (DE/pathway size)**	***p.adj.* value**
Proteoglycans in cancer	KEGG:05205	21	196	0.107143	2.57E-05
ECM-receptor interaction	KEGG:04512	12	87	0.137931	0.000582
TNF signaling pathway	KEGG:04668	13	109	0.119266	0.0008
Focal adhesion	KEGG:04510	17	194	0.087629	0.001357
Amoebiasis	KEGG:05146	12	103	0.116505	0.001357
Small cell lung cancer	KEGG:05222	10	88	0.113636	0.006649
IL-17 signaling pathway	KEGG:04657	9	88	0.102273	0.026794
Salmonella infection	KEGG:05132	8	75	0.106667	0.035034
Pathways in cancer	KEGG:05200	27	522	0.051724	0.035895
Cell adhesion molecules (CAMs)	KEGG:04514	12	159	0.075472	0.040351
Rheumatoid arthritis	KEGG:05323	8	83	0.096386	0.049291

Appendix Table S4. REACTOME enriched pathways between *Rage* control and *Rage* knockout BECs.

**REACTOME enriched pathways**					
**Pathway name**	**Pathway code**	**DE genes**	**Pathway size**	**Fraction (DE/pathway size)**	***p.adj*. value**
Extracellular matrix organization	REAC:R-MMU-1474244	23	253	0.090909	0.000405
Laminin interactions	REAC:R-MMU-3000157	6	25	0.24	0.011944
Non-integrin membrane-ECM interactions	REAC:R-MMU-3000171	7	34	0.205882	0.011944
Integrin cell surface interactions	REAC:R-MMU-216083	9	67	0.134328	0.016526
