# Supplementary figures and images for: RAGE is a key regulator of ductular reaction-mediated fibrosis during cholestasis (part 1 of 2)
Source: EMBO Rep. 2025 Jan 2;26(3):880–907. doi: 10.1038/s44319-024-00356-7 (PMC11811172; doi:10.1038/s44319-024-00356-7)

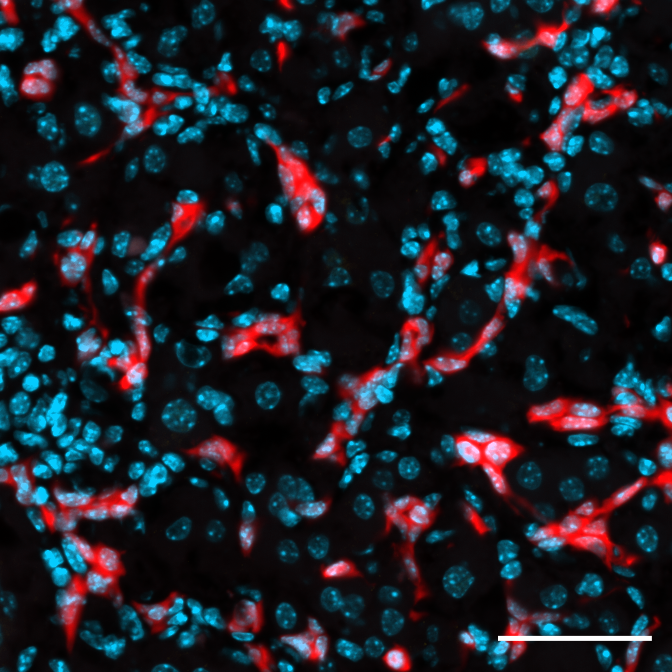

Supplement: Supplementary file 3 — Source data Fig. 1 [file 44319_2024_356_MOESM3_ESM.zip › Figure 1/1C/HET_22192_GFP.tif]

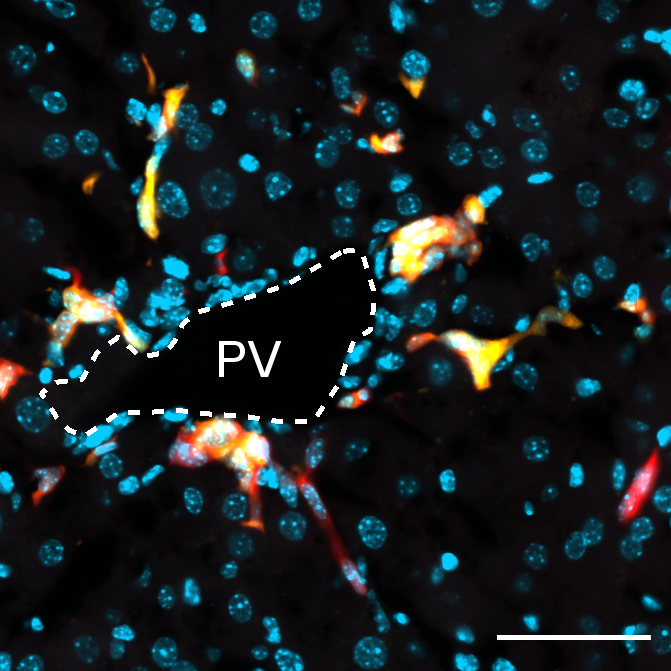

Supplement: Supplementary file 3 — Source data Fig. 1 [file 44319_2024_356_MOESM3_ESM.zip › Figure 1/1C/KO_22294_GFP.tif]

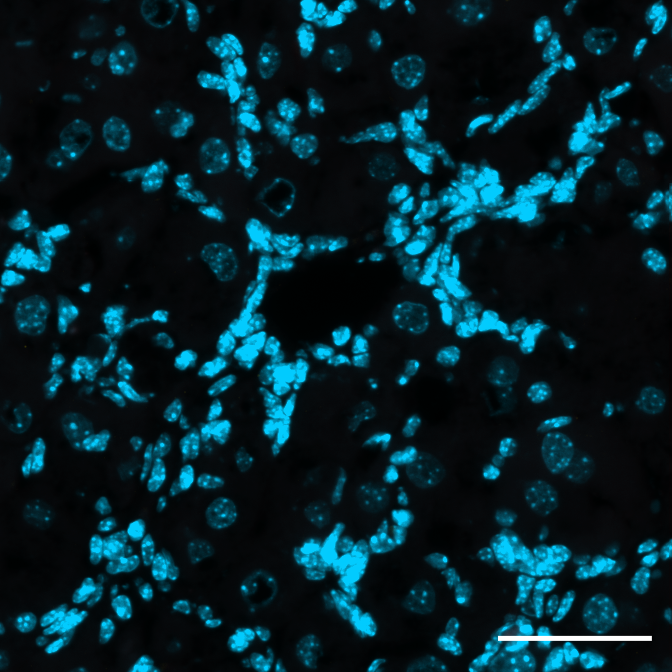

Supplement: Supplementary file 3 — Source data Fig. 1 [file 44319_2024_356_MOESM3_ESM.zip › Figure 1/1C/WT_24288_GFP.tif]

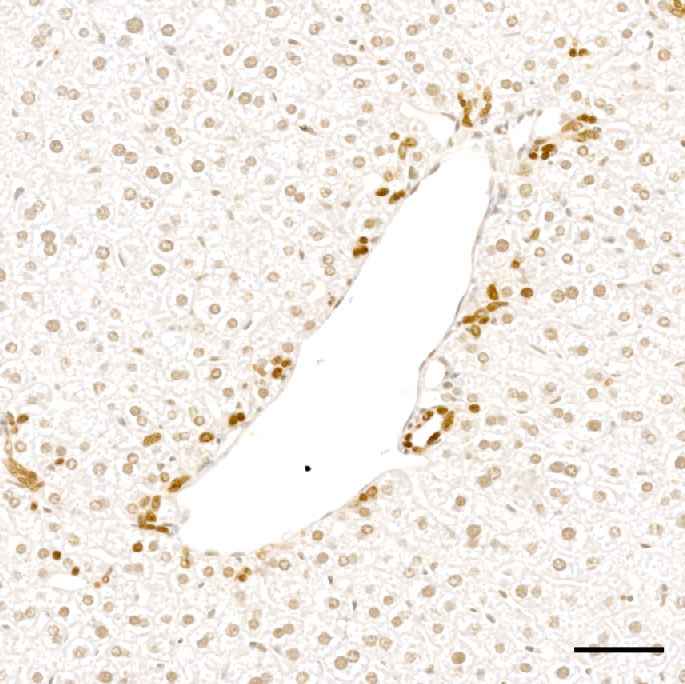

Supplement: Supplementary file 3 — Source data Fig. 1 [file 44319_2024_356_MOESM3_ESM.zip › Figure 1/1D/HNF1B/1_ND_WT_m410_19_hnf1b.png]

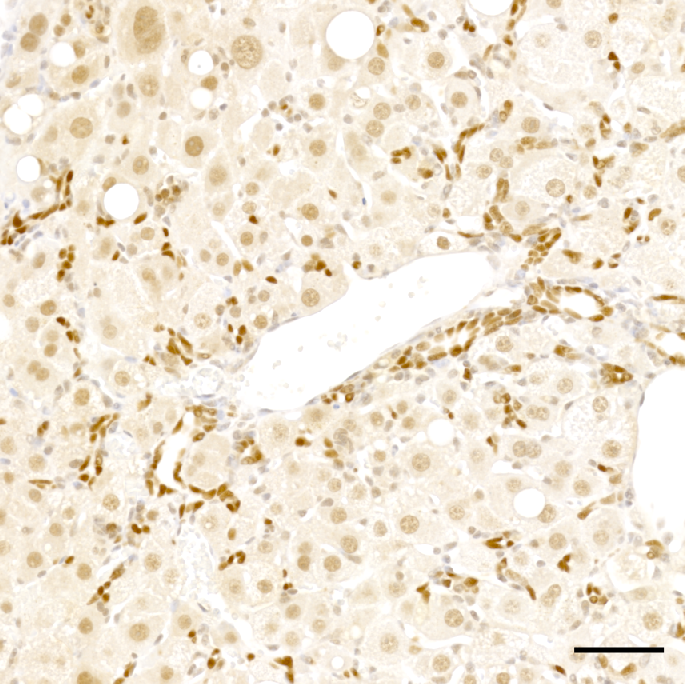

Supplement: Supplementary file 3 — Source data Fig. 1 [file 44319_2024_356_MOESM3_ESM.zip › Figure 1/1D/HNF1B/2_CDE_WT_m488_19_hnf1b.png]

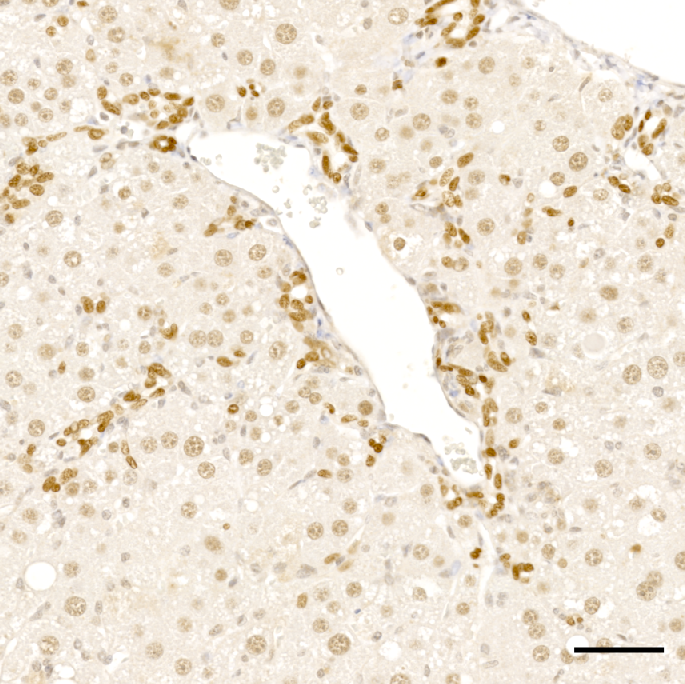

Supplement: Supplementary file 3 — Source data Fig. 1 [file 44319_2024_356_MOESM3_ESM.zip › Figure 1/1D/HNF1B/3_CDE_HET_m25_19_hnf1b.png]

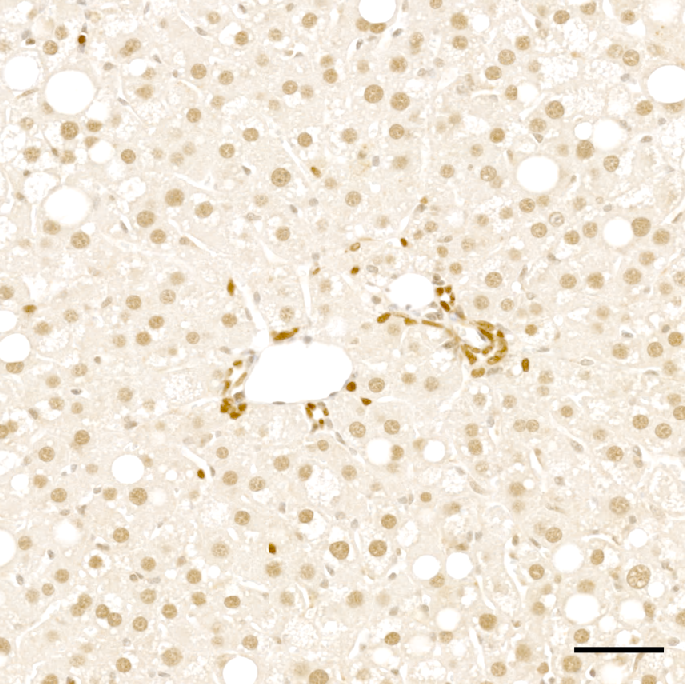

Supplement: Supplementary file 3 — Source data Fig. 1 [file 44319_2024_356_MOESM3_ESM.zip › Figure 1/1D/HNF1B/4_CDE_KO_m416_19_hnf1b.png]

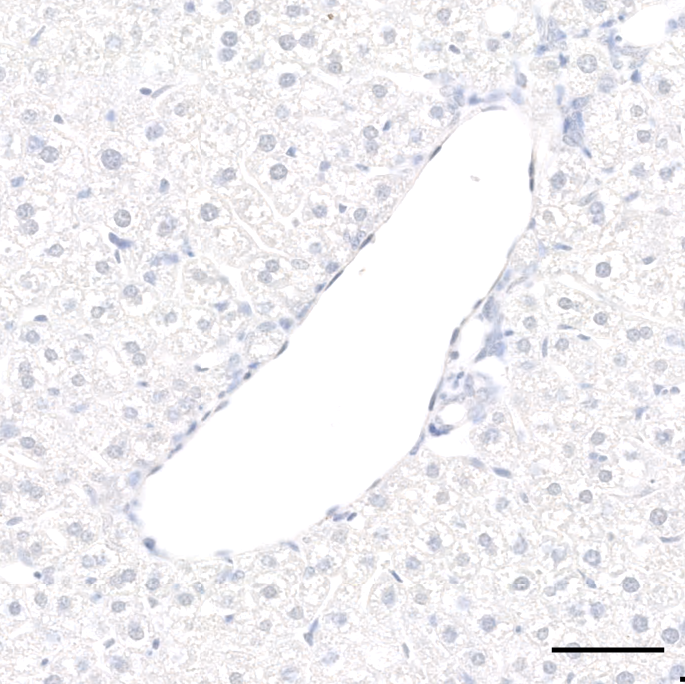

Supplement: Supplementary file 3 — Source data Fig. 1 [file 44319_2024_356_MOESM3_ESM.zip › Figure 1/1D/mCherry/1_ND_WT_m410_19_mCherry.png]

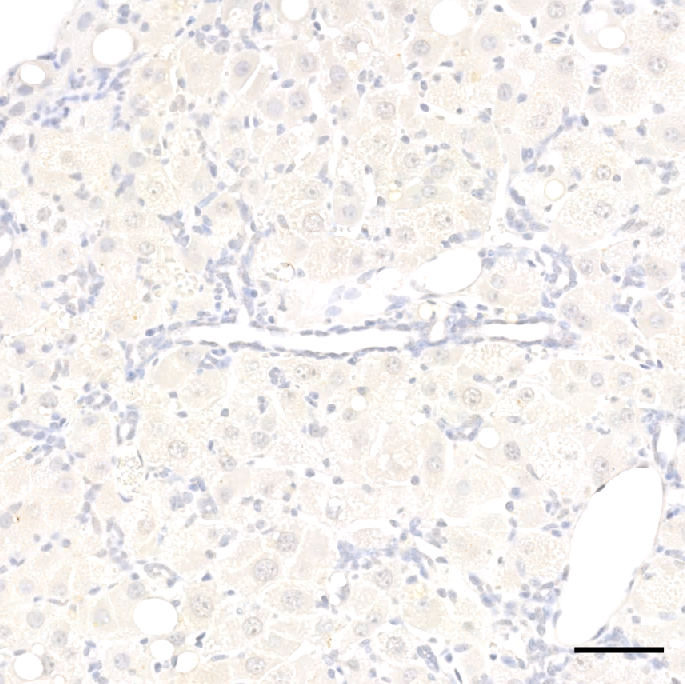

Supplement: Supplementary file 3 — Source data Fig. 1 [file 44319_2024_356_MOESM3_ESM.zip › Figure 1/1D/mCherry/2_CDE_WT_m488_19_mCherry.png]

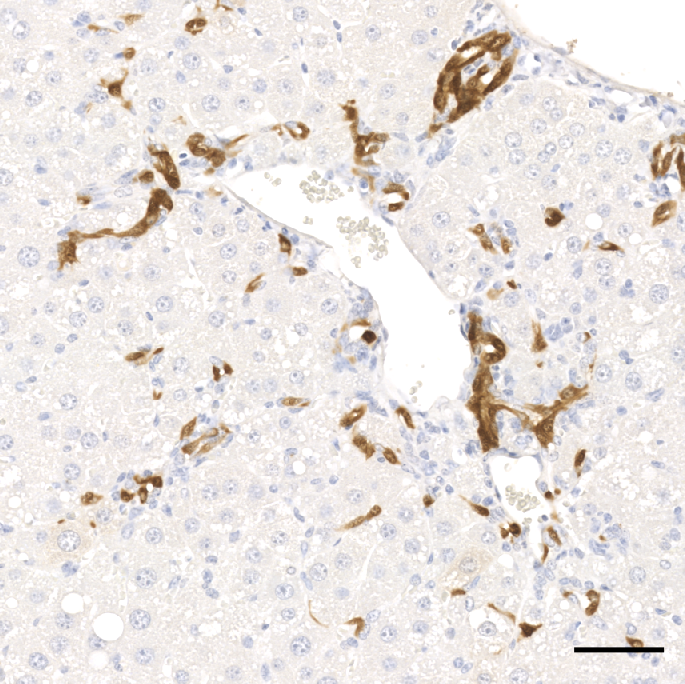

Supplement: Supplementary file 3 — Source data Fig. 1 [file 44319_2024_356_MOESM3_ESM.zip › Figure 1/1D/mCherry/3_CDE_Het_m25_19_mcherry.png]

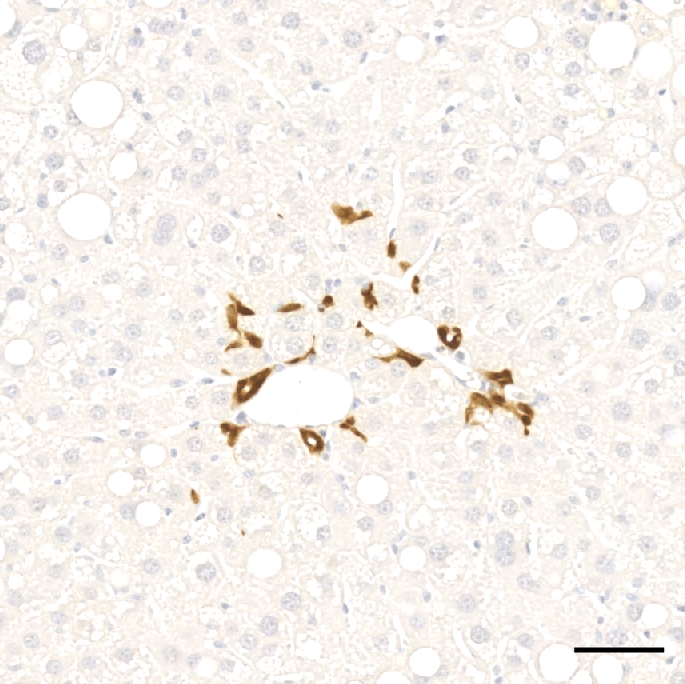

Supplement: Supplementary file 3 — Source data Fig. 1 [file 44319_2024_356_MOESM3_ESM.zip › Figure 1/1D/mCherry/4_CDE_KO_m416_19_mCherry.png]

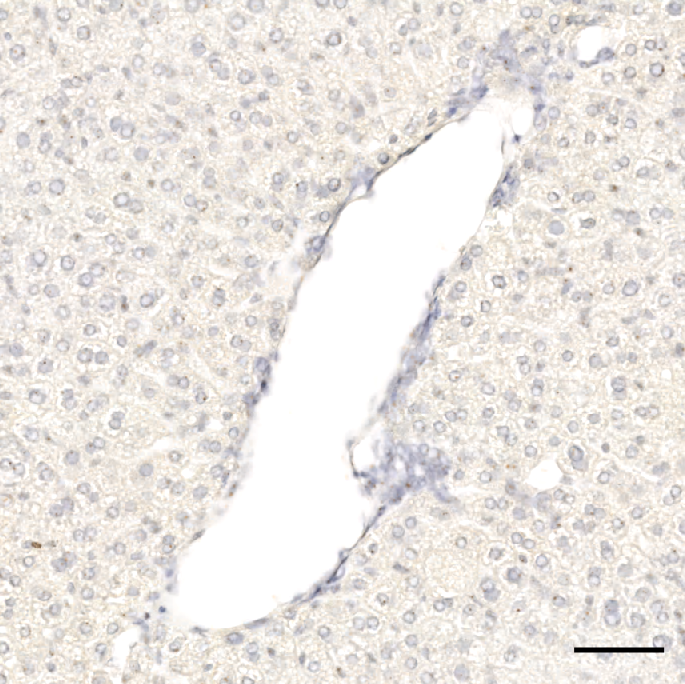

Supplement: Supplementary file 3 — Source data Fig. 1 [file 44319_2024_356_MOESM3_ESM.zip › Figure 1/1D/RAGE/1_ND_WT_m410_19_Rage.png]

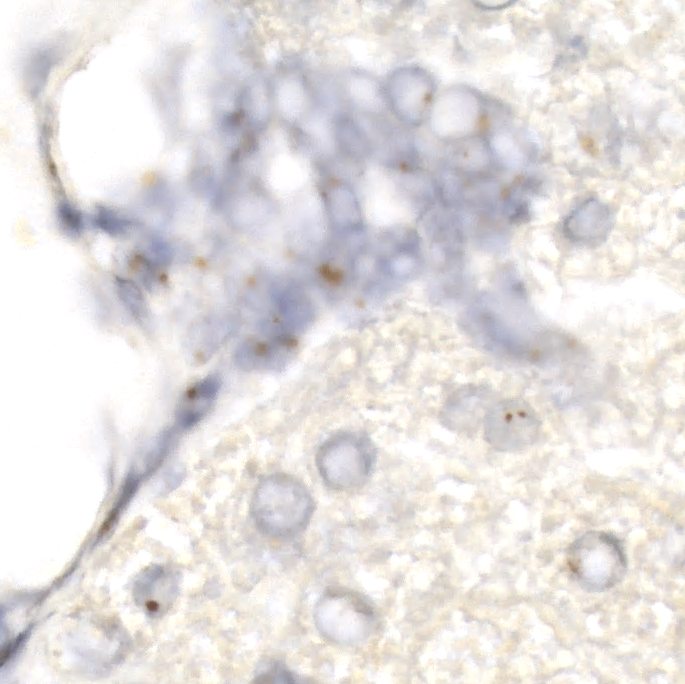

Supplement: Supplementary file 3 — Source data Fig. 1 [file 44319_2024_356_MOESM3_ESM.zip › Figure 1/1D/RAGE/1_ND_WT_m410_19_Rage_zoom.png]

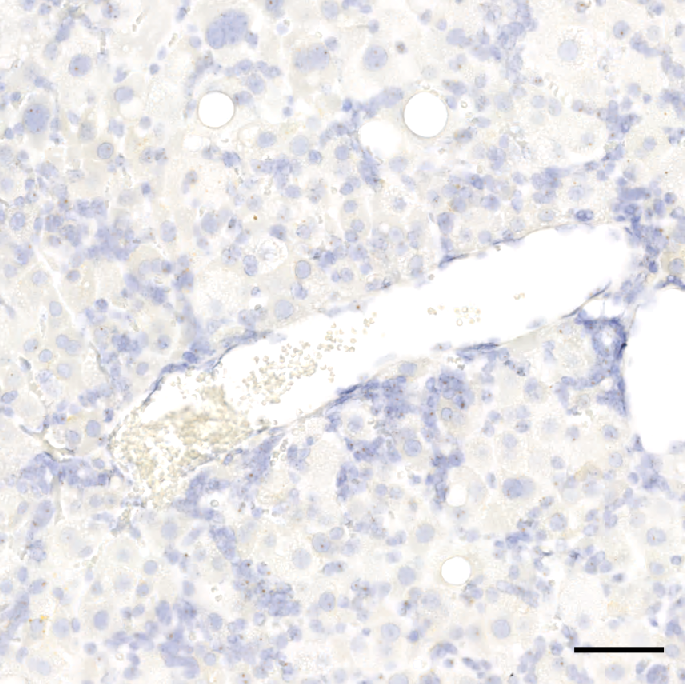

Supplement: Supplementary file 3 — Source data Fig. 1 [file 44319_2024_356_MOESM3_ESM.zip › Figure 1/1D/RAGE/2_CDE_WT_m488_19_Rage.png]

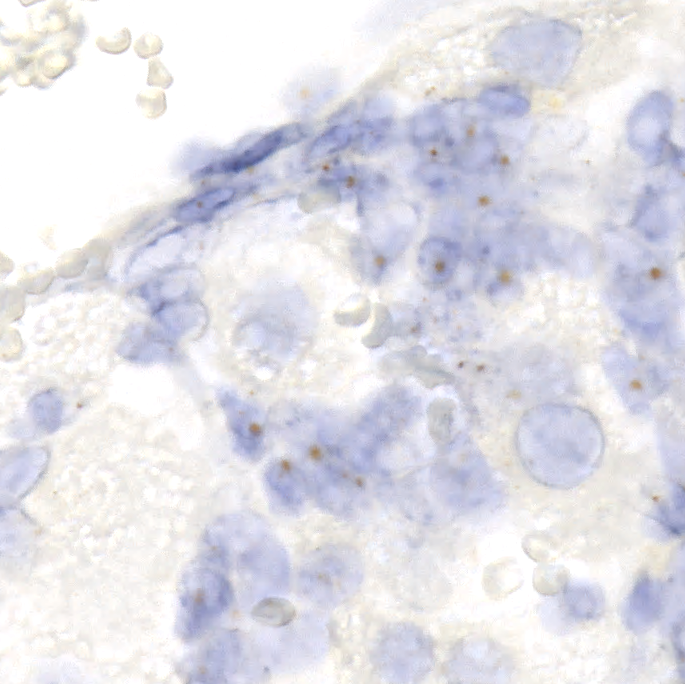

Supplement: Supplementary file 3 — Source data Fig. 1 [file 44319_2024_356_MOESM3_ESM.zip › Figure 1/1D/RAGE/2_CDE_WT_m488_19_Rage_zoom.png]

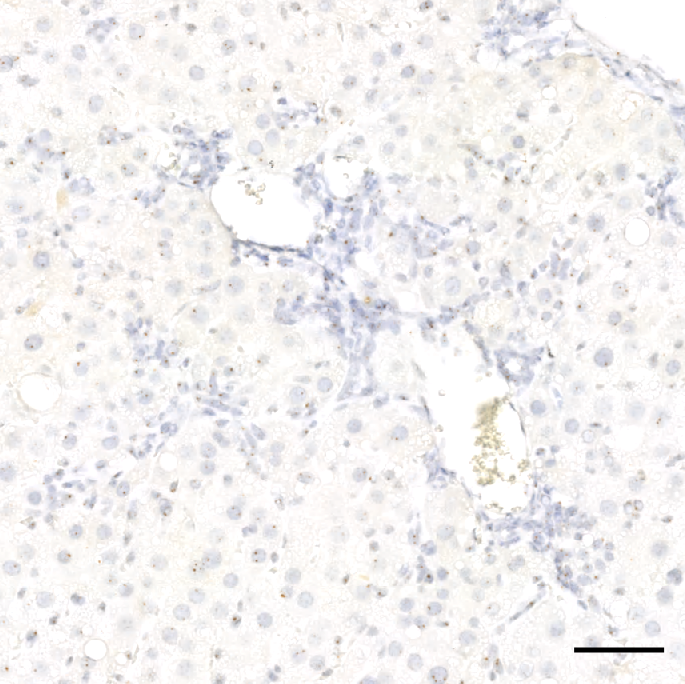

Supplement: Supplementary file 3 — Source data Fig. 1 [file 44319_2024_356_MOESM3_ESM.zip › Figure 1/1D/RAGE/3_CDE_HET_m25_19_Rage.png]

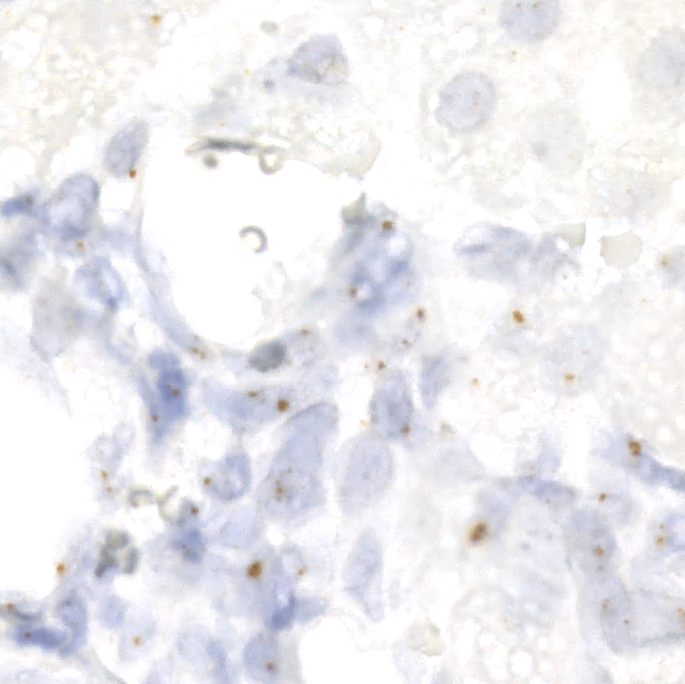

Supplement: Supplementary file 3 — Source data Fig. 1 [file 44319_2024_356_MOESM3_ESM.zip › Figure 1/1D/RAGE/3_CDE_HET_m25_19_Rage_zoom.png]

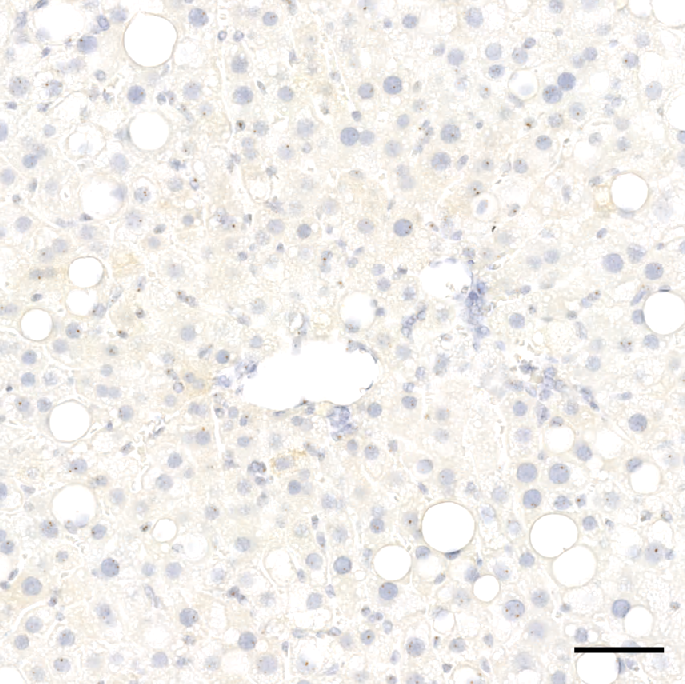

Supplement: Supplementary file 3 — Source data Fig. 1 [file 44319_2024_356_MOESM3_ESM.zip › Figure 1/1D/RAGE/4_CDE_KO_m416_19_Rage.png]

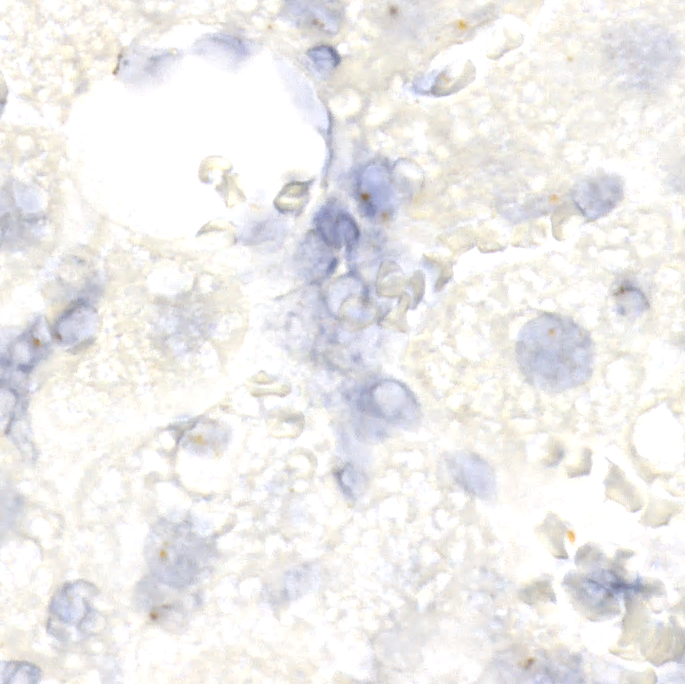

Supplement: Supplementary file 3 — Source data Fig. 1 [file 44319_2024_356_MOESM3_ESM.zip › Figure 1/1D/RAGE/4_CDE_KO_m416_19_Rage_zoom.png]

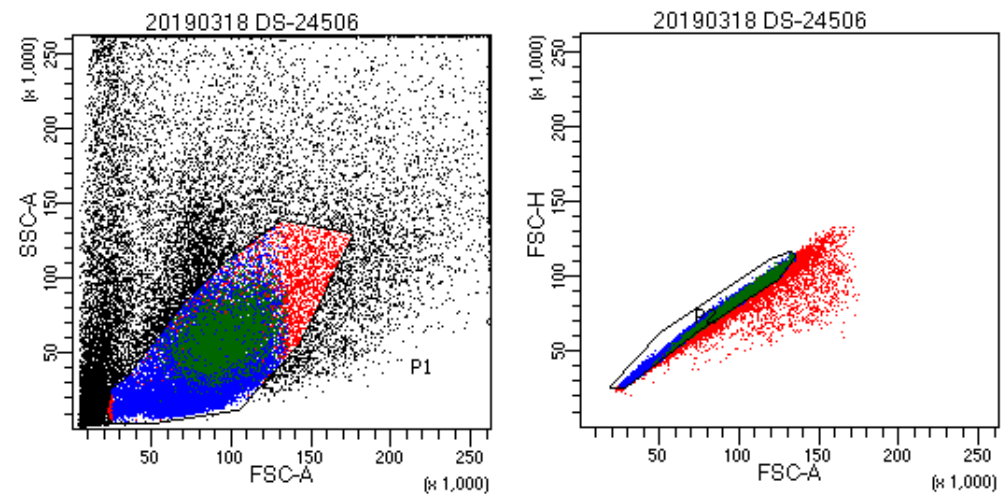

Tube: 24506

| Population | #Events | %Parent | %Total |
|------------|---------|---------|--------|
| All Events | 54,943  | ####    | 100.0  |
| P1         | 37,205  | 67.7    | 67.7   |
| P2         | 32,406  | 87.1    | 59.0   |
| P3         | 410     | 1.3     | 0.7    |
| P4         | 4,312   | 13.3    | 7.8    |

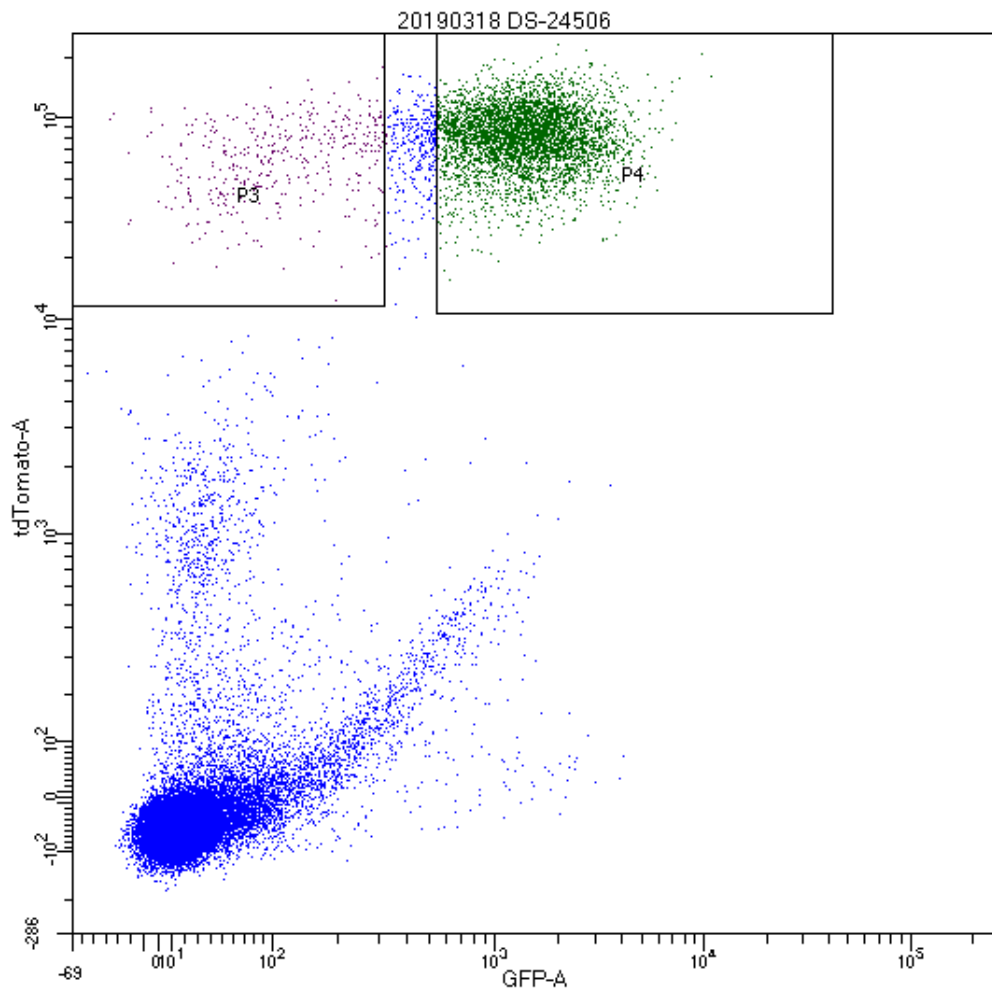

|       | p3     | p4      |
|-------|--------|---------|
| 24461 |        |         |
| 24506 | 28.200 | 290.100 |

Supplement: Supplementary file 4 — Source data Fig. 2 [file 44319_2024_356_MOESM4_ESM.zip › Figure 2/2B/Batch 3/20190318 DS_24506_18032019153019.pdf]

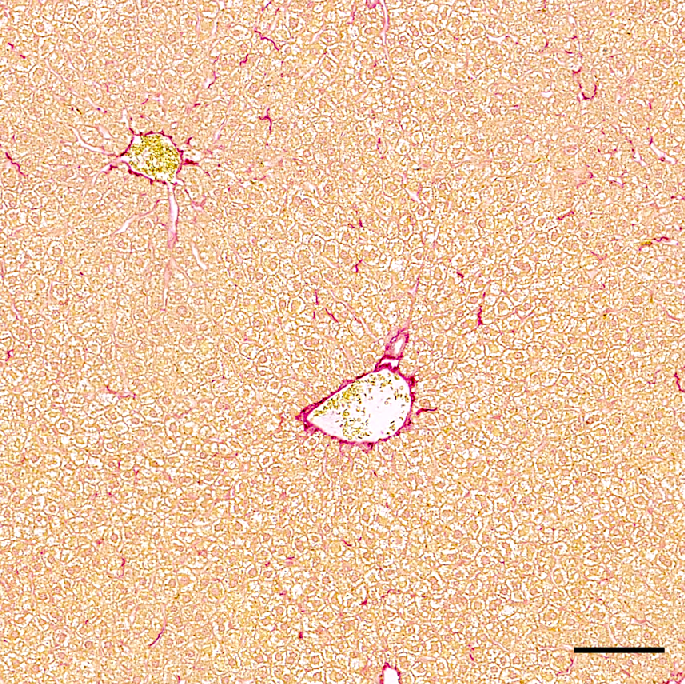

Supplement: Supplementary file 5 — Source data Fig. 3 [file 44319_2024_356_MOESM5_ESM.zip › Figure 3/3A/1_ND_WT_m472_2019_Sirius Red.tif]

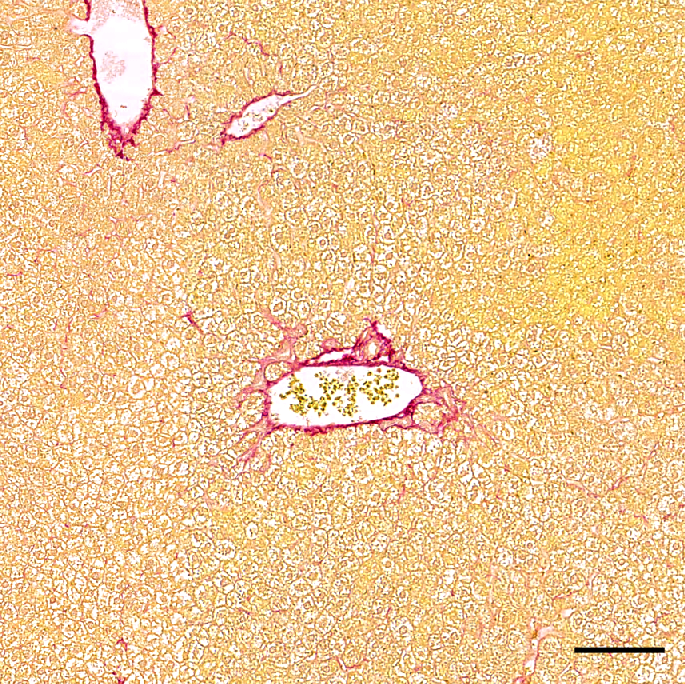

Supplement: Supplementary file 5 — Source data Fig. 3 [file 44319_2024_356_MOESM5_ESM.zip › Figure 3/3A/2_ND_HET_m422_2019_Sirius Red.tif]

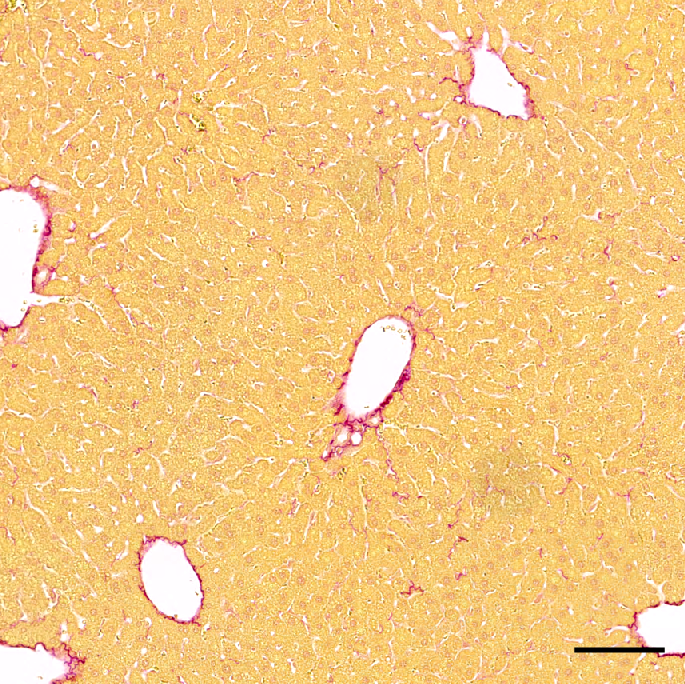

Supplement: Supplementary file 5 — Source data Fig. 3 [file 44319_2024_356_MOESM5_ESM.zip › Figure 3/3A/3_ND_KO_m466_2019_Sirius Re.tif]

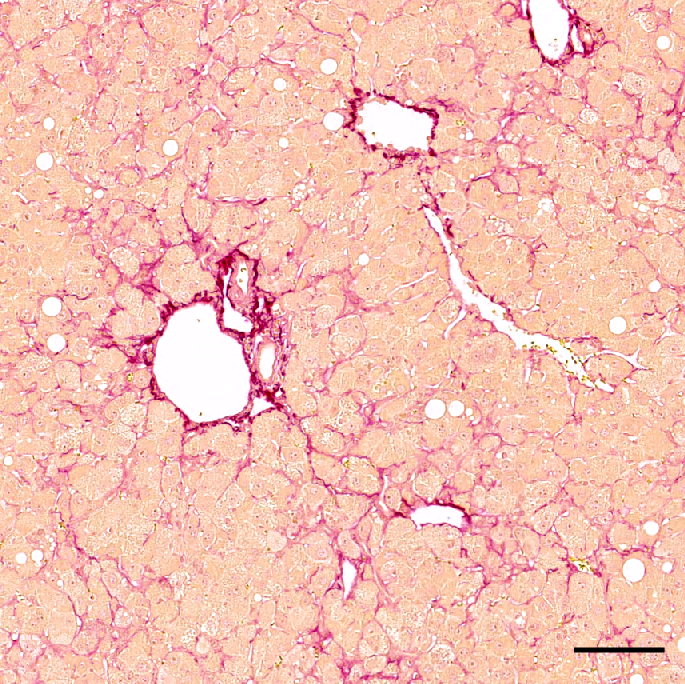

Supplement: Supplementary file 5 — Source data Fig. 3 [file 44319_2024_356_MOESM5_ESM.zip › Figure 3/3A/4_CDE_WT_m486_2019_Sirius Red.tif]

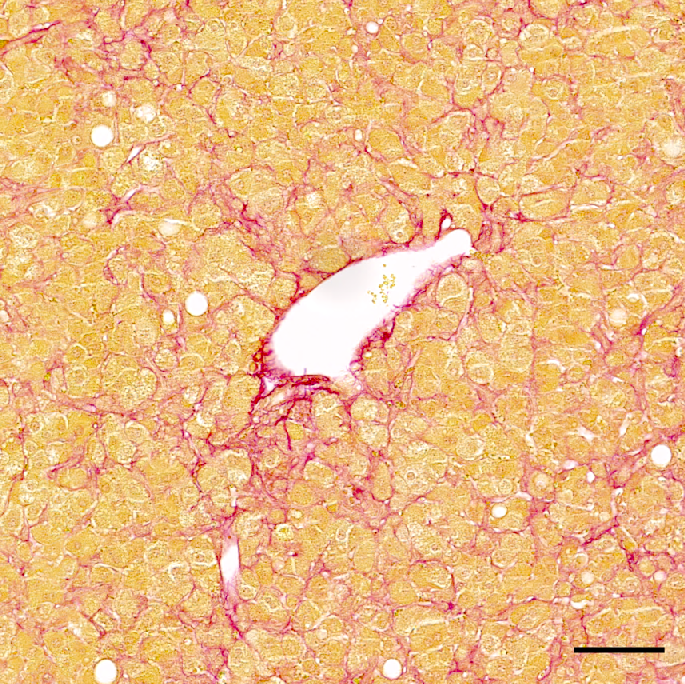

Supplement: Supplementary file 5 — Source data Fig. 3 [file 44319_2024_356_MOESM5_ESM.zip › Figure 3/3A/5_CDE_HET_m414_2019_Sirius Red.tif]

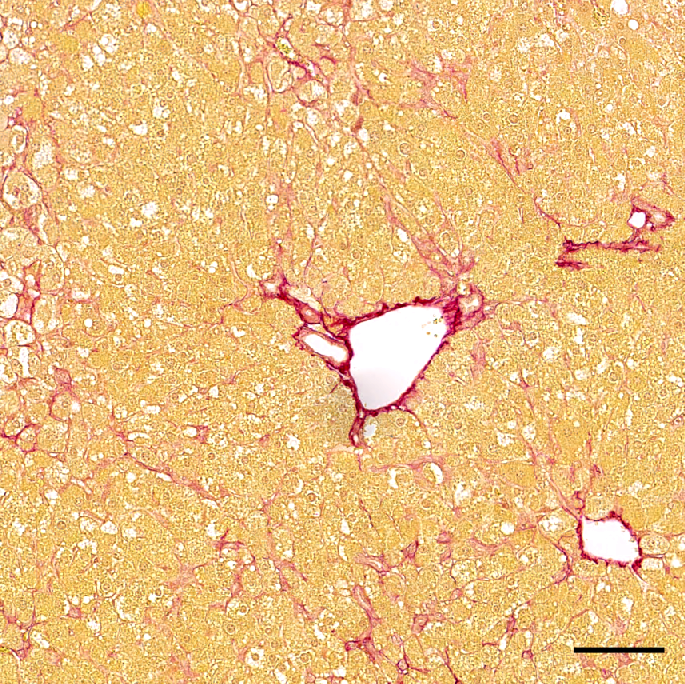

Supplement: Supplementary file 5 — Source data Fig. 3 [file 44319_2024_356_MOESM5_ESM.zip › Figure 3/3A/6_CDE_KO_m436_2019_Sirius Red.tif]

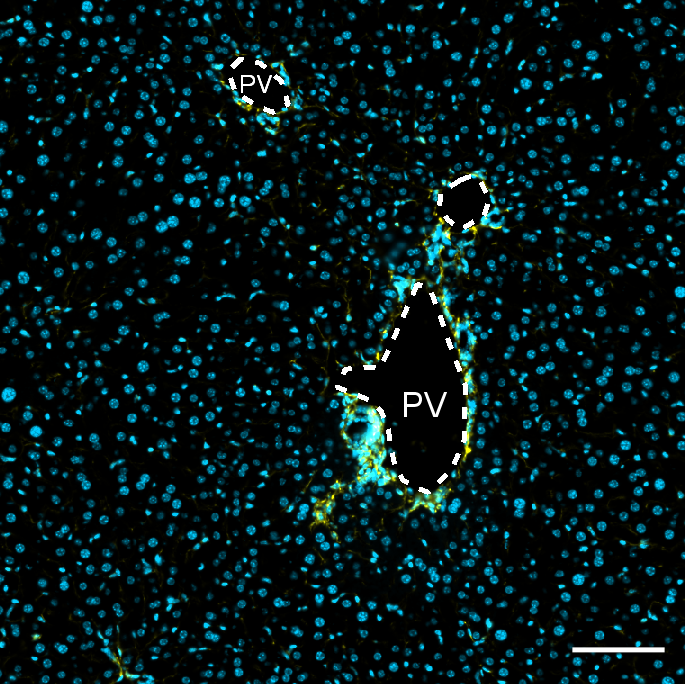

Supplement: Supplementary file 6 — Source data Fig. 4 [file 44319_2024_356_MOESM6_ESM.zip › Figure 4/4A/1_ND_WT_30007_vim.tif]

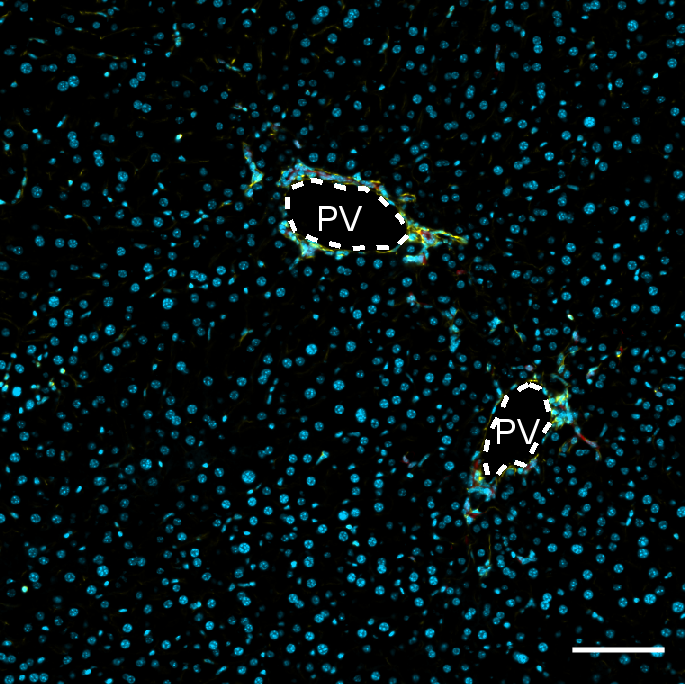

Supplement: Supplementary file 6 — Source data Fig. 4 [file 44319_2024_356_MOESM6_ESM.zip › Figure 4/4A/2_ND_HET_29765_vim.tif]

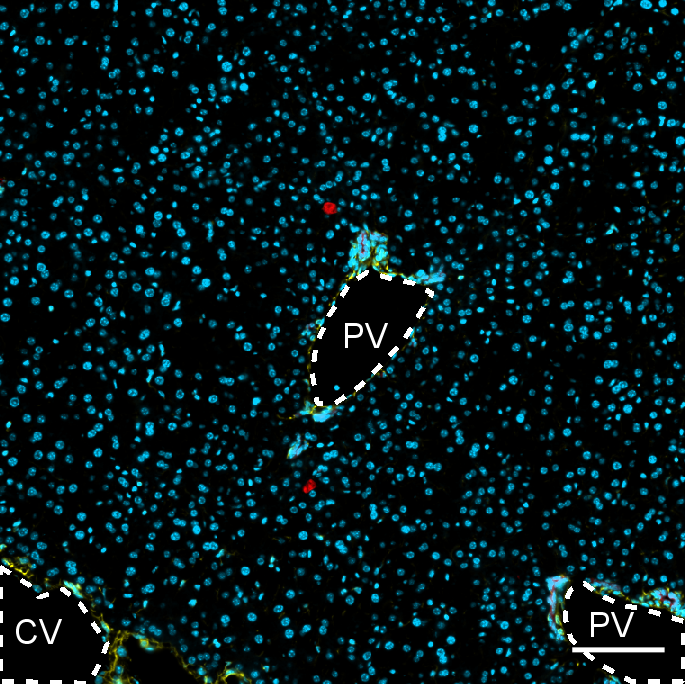

Supplement: Supplementary file 6 — Source data Fig. 4 [file 44319_2024_356_MOESM6_ESM.zip › Figure 4/4A/3_ND_KO_29820_vim.tif]

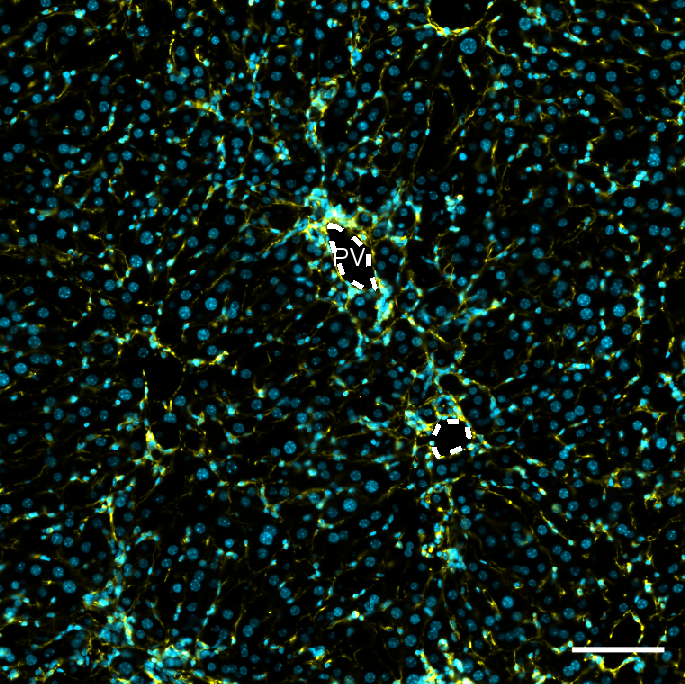

Supplement: Supplementary file 6 — Source data Fig. 4 [file 44319_2024_356_MOESM6_ESM.zip › Figure 4/4A/4_CDE_WT_25505_vim.tif]

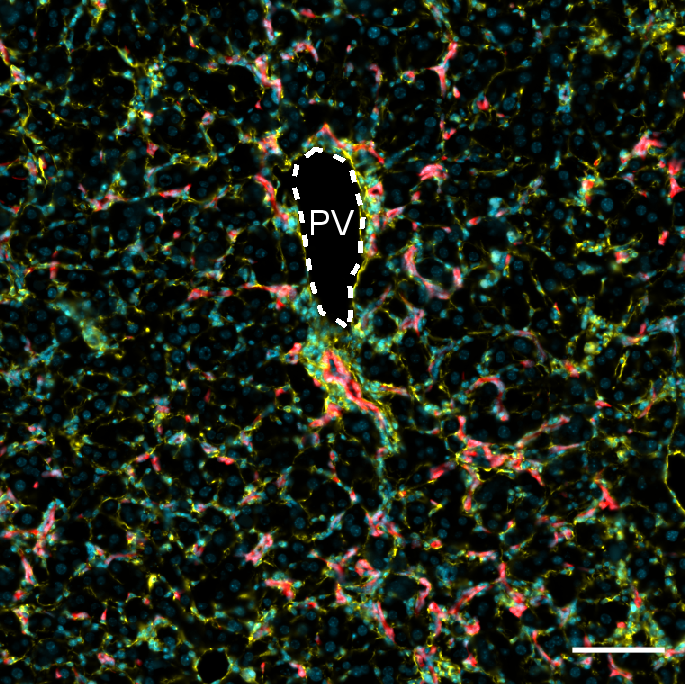

Supplement: Supplementary file 6 — Source data Fig. 4 [file 44319_2024_356_MOESM6_ESM.zip › Figure 4/4A/5_CDE_HET_29238_vim.tif]

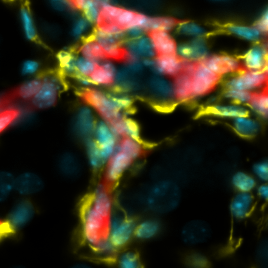

Supplement: Supplementary file 6 — Source data Fig. 4 [file 44319_2024_356_MOESM6_ESM.zip › Figure 4/4A/5_CDE_HET_29238_vim_Split channel/5_29238_vim_250_-Image Export-01_b0s0c0-3x0-268y0-268.png]

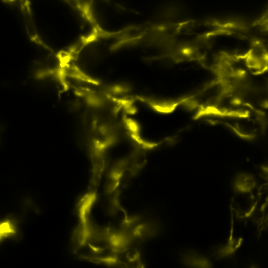

Supplement: Supplementary file 6 — Source data Fig. 4 [file 44319_2024_356_MOESM6_ESM.zip › Figure 4/4A/5_CDE_HET_29238_vim_Split channel/5_29238_vim_250_-Image Export-01_b0s0c0x0-268y0-268.png]

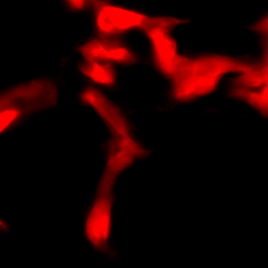

Supplement: Supplementary file 6 — Source data Fig. 4 [file 44319_2024_356_MOESM6_ESM.zip › Figure 4/4A/5_CDE_HET_29238_vim_Split channel/5_29238_vim_250_-Image Export-01_b0s0c1x0-268y0-268.png]

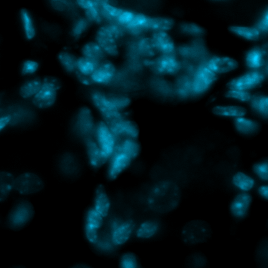

Supplement: Supplementary file 6 — Source data Fig. 4 [file 44319_2024_356_MOESM6_ESM.zip › Figure 4/4A/5_CDE_HET_29238_vim_Split channel/5_29238_vim_250_-Image Export-01_b0s0c2x0-268y0-268.png]

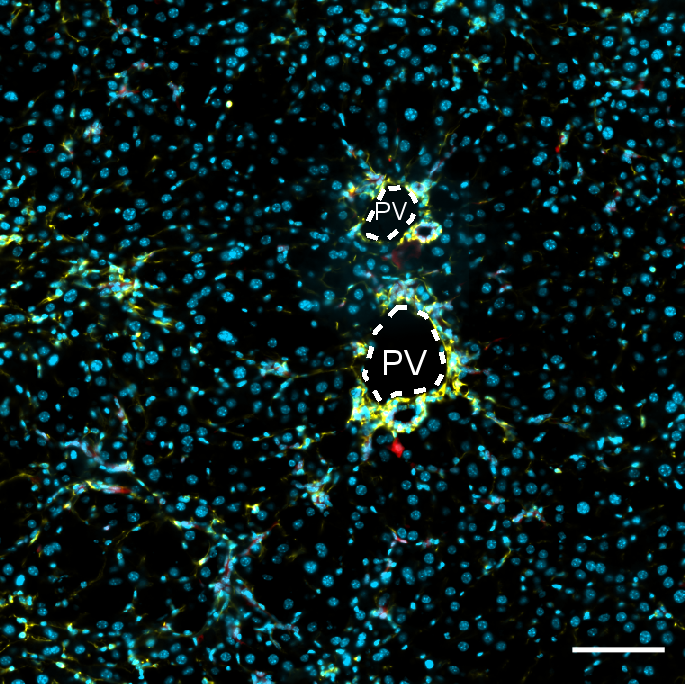

Supplement: Supplementary file 6 — Source data Fig. 4 [file 44319_2024_356_MOESM6_ESM.zip › Figure 4/4A/6_CDE_KO_29840_vim.tif]

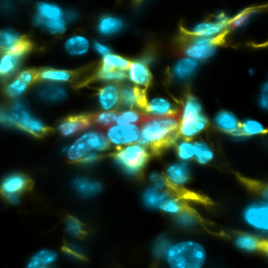

Supplement: Supplementary file 6 — Source data Fig. 4 [file 44319_2024_356_MOESM6_ESM.zip › Figure 4/4A/6_CDE_KO_29840_vim_Split channel/6_29840_vim_250_-Image Export-02_b0s0c0-3x0-268y0-268.png]

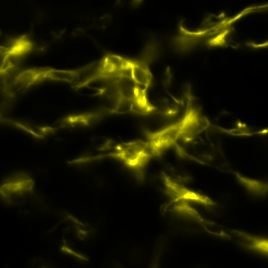

Supplement: Supplementary file 6 — Source data Fig. 4 [file 44319_2024_356_MOESM6_ESM.zip › Figure 4/4A/6_CDE_KO_29840_vim_Split channel/6_29840_vim_250_-Image Export-02_b0s0c0x0-268y0-268.png]

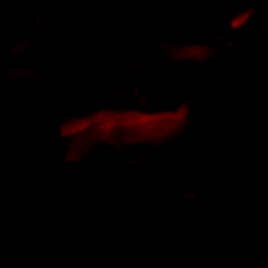

Supplement: Supplementary file 6 — Source data Fig. 4 [file 44319_2024_356_MOESM6_ESM.zip › Figure 4/4A/6_CDE_KO_29840_vim_Split channel/6_29840_vim_250_-Image Export-02_b0s0c1x0-268y0-268.png]

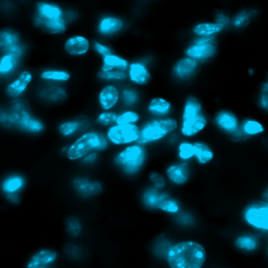

Supplement: Supplementary file 6 — Source data Fig. 4 [file 44319_2024_356_MOESM6_ESM.zip › Figure 4/4A/6_CDE_KO_29840_vim_Split channel/6_29840_vim_250_-Image Export-02_b0s0c2x0-268y0-268.png]

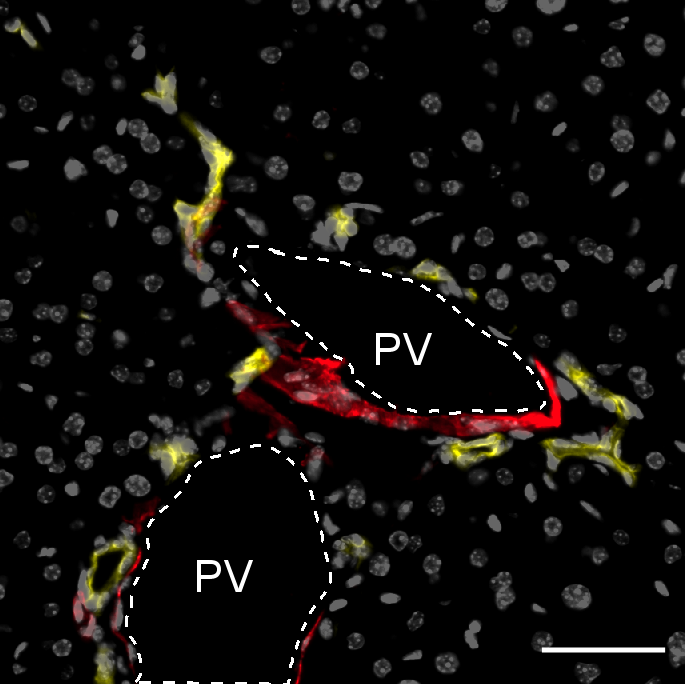

Supplement: Supplementary file 6 — Source data Fig. 4 [file 44319_2024_356_MOESM6_ESM.zip › Figure 4/4C/1_ND_WT_30007.tif]

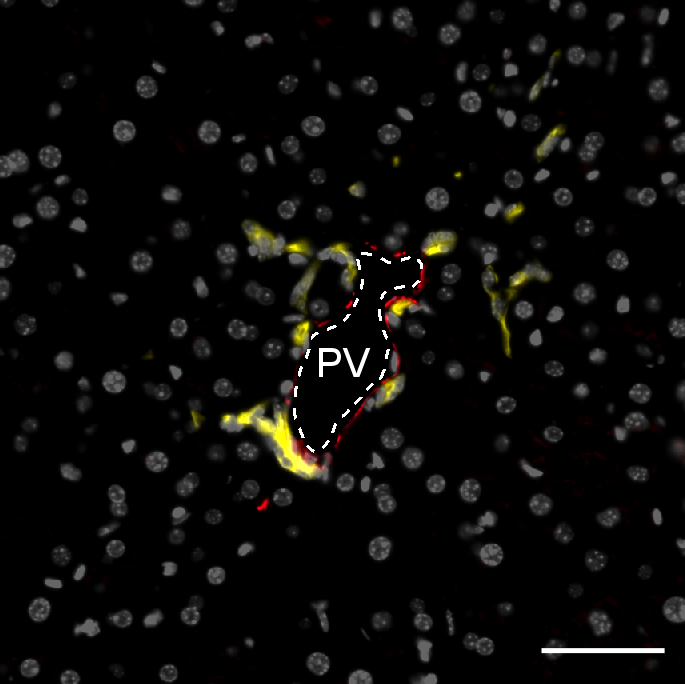

Supplement: Supplementary file 6 — Source data Fig. 4 [file 44319_2024_356_MOESM6_ESM.zip › Figure 4/4C/2_ND_Het_24676.tif]

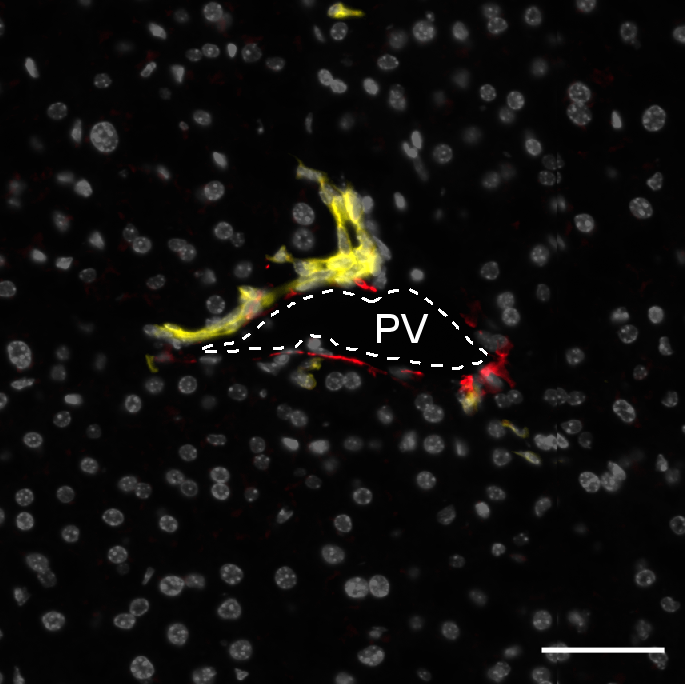

Supplement: Supplementary file 6 — Source data Fig. 4 [file 44319_2024_356_MOESM6_ESM.zip › Figure 4/4C/3_ND_KO_29320.tif]

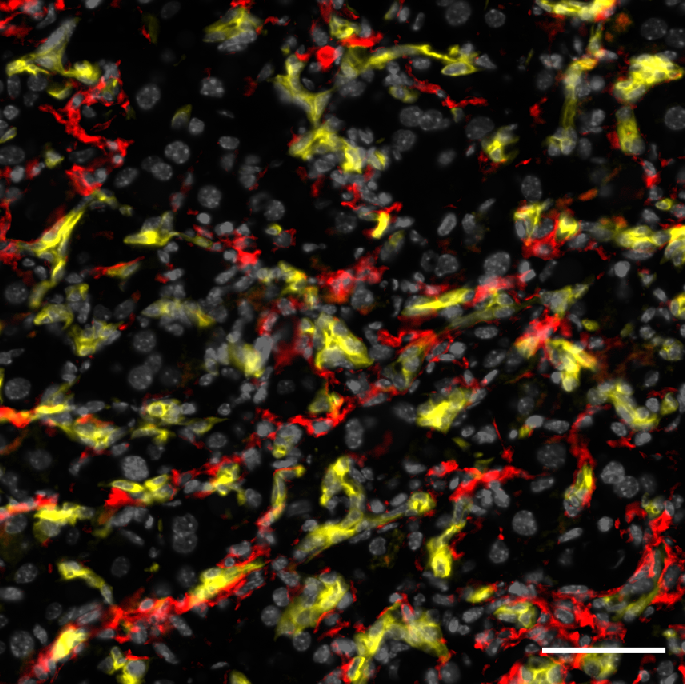

Supplement: Supplementary file 6 — Source data Fig. 4 [file 44319_2024_356_MOESM6_ESM.zip › Figure 4/4C/4_CDE_WT_24463.tif]

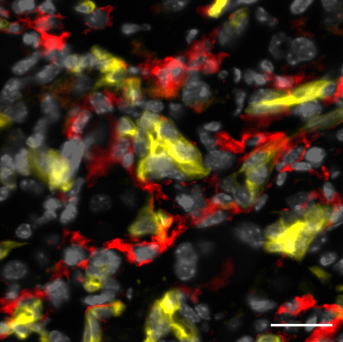

Supplement: Supplementary file 6 — Source data Fig. 4 [file 44319_2024_356_MOESM6_ESM.zip › Figure 4/4C/4_CDE_WT_24463_zoom.tif]

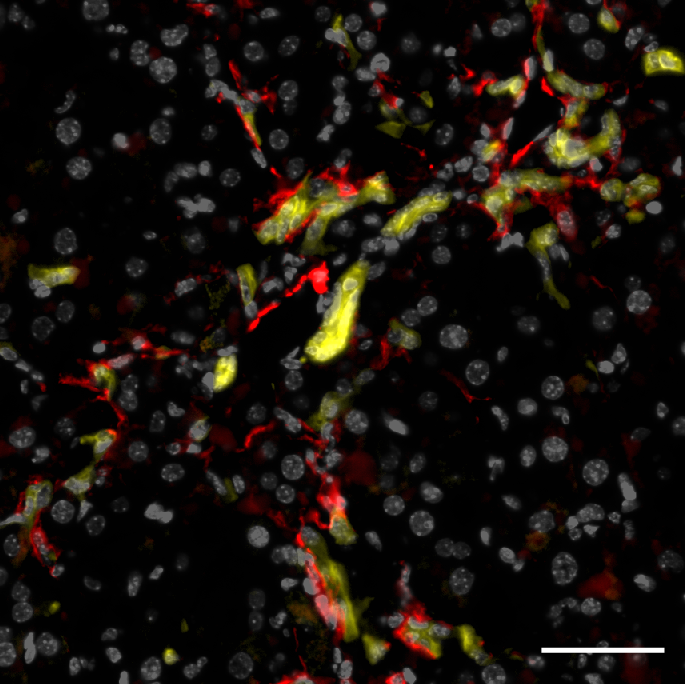

Supplement: Supplementary file 6 — Source data Fig. 4 [file 44319_2024_356_MOESM6_ESM.zip › Figure 4/4C/5_CDE_Het_22192.tif]

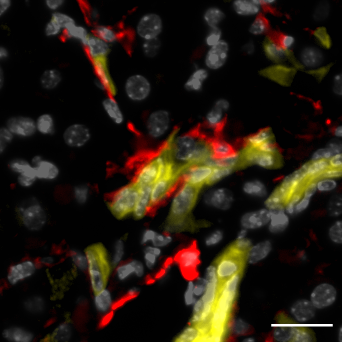

Supplement: Supplementary file 6 — Source data Fig. 4 [file 44319_2024_356_MOESM6_ESM.zip › Figure 4/4C/5_CDE_Het_22192_zoom.tif]

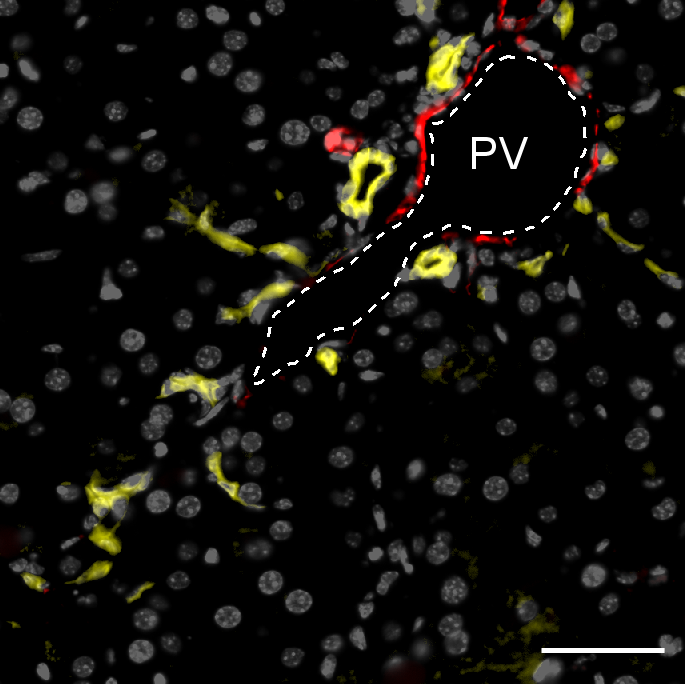

Supplement: Supplementary file 6 — Source data Fig. 4 [file 44319_2024_356_MOESM6_ESM.zip › Figure 4/4C/6_CDE_KO_29254.tif]

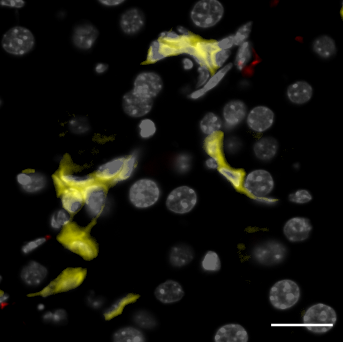

Supplement: Supplementary file 6 — Source data Fig. 4 [file 44319_2024_356_MOESM6_ESM.zip › Figure 4/4C/6_CDE_KO_29254_zoom.tif]

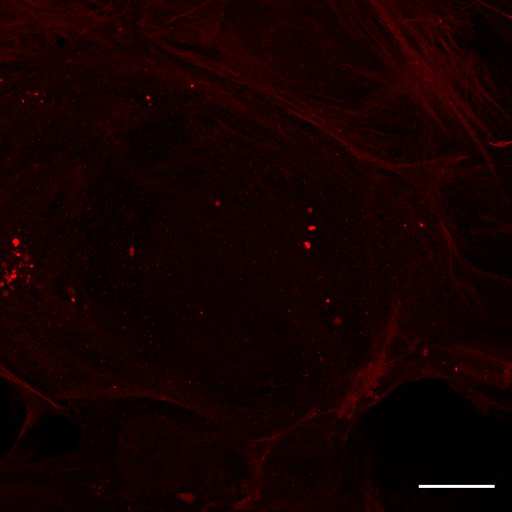

Supplement: Supplementary file 8 — Source data Fig. 6 [file 44319_2024_356_MOESM8_ESM.zip › Figure 6/6A/1_Control/con_aSMA.png]

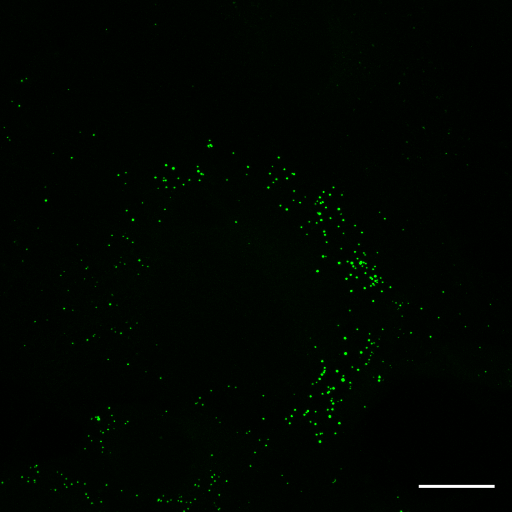

Supplement: Supplementary file 8 — Source data Fig. 6 [file 44319_2024_356_MOESM8_ESM.zip › Figure 6/6A/1_Control/con_BODIPY.png]

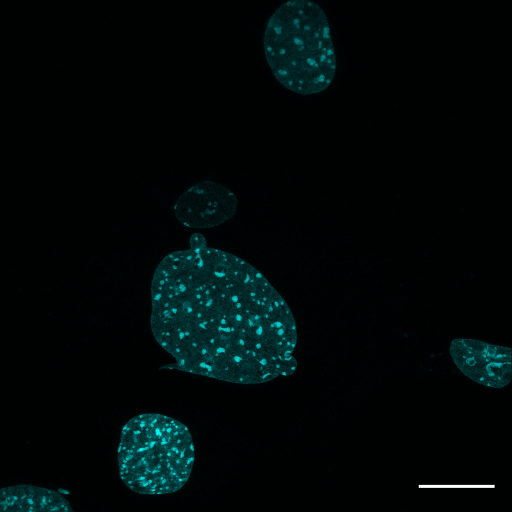

Supplement: Supplementary file 8 — Source data Fig. 6 [file 44319_2024_356_MOESM8_ESM.zip › Figure 6/6A/1_Control/con_DAPI.png]

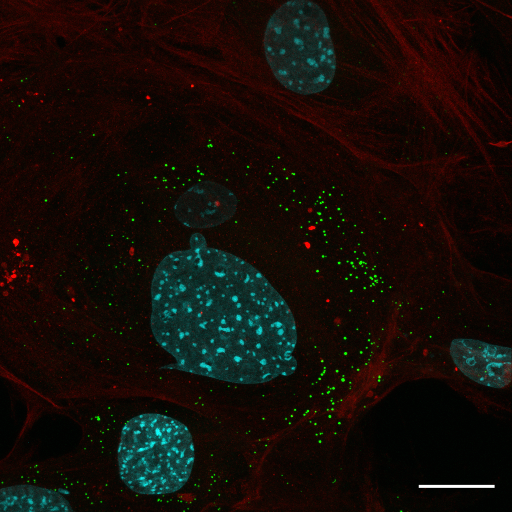

Supplement: Supplementary file 8 — Source data Fig. 6 [file 44319_2024_356_MOESM8_ESM.zip › Figure 6/6A/1_Control/con_merge.png]

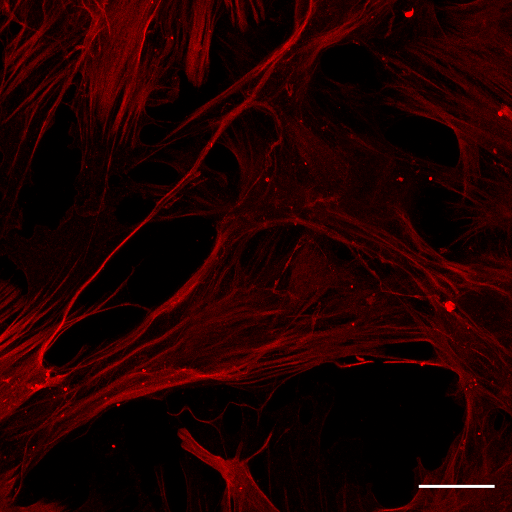

Supplement: Supplementary file 8 — Source data Fig. 6 [file 44319_2024_356_MOESM8_ESM.zip › Figure 6/6A/2_rTGFB/rTGFB_aSMA.png]

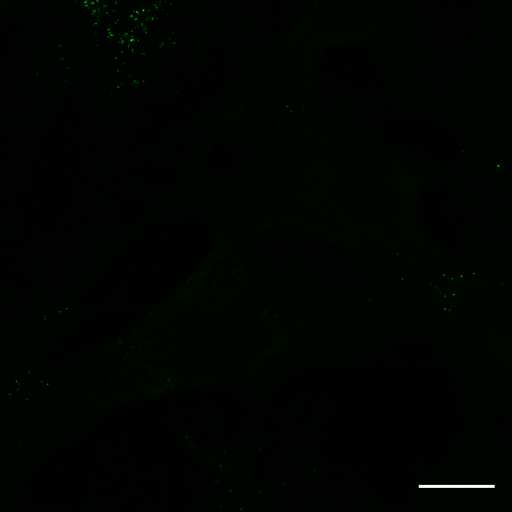

Supplement: Supplementary file 8 — Source data Fig. 6 [file 44319_2024_356_MOESM8_ESM.zip › Figure 6/6A/2_rTGFB/rTGFB_BODIPY.png]

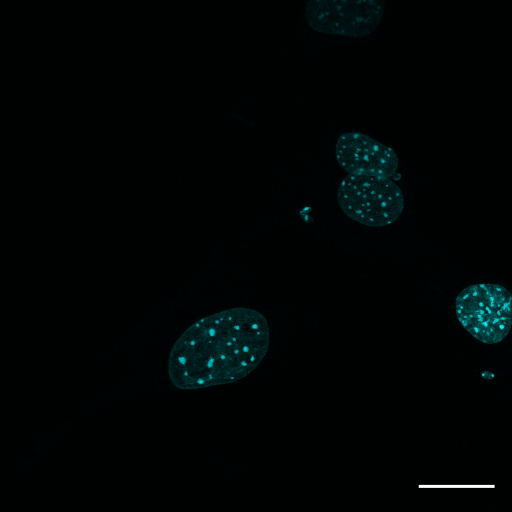

Supplement: Supplementary file 8 — Source data Fig. 6 [file 44319_2024_356_MOESM8_ESM.zip › Figure 6/6A/2_rTGFB/rTGFB_DAPI.png]

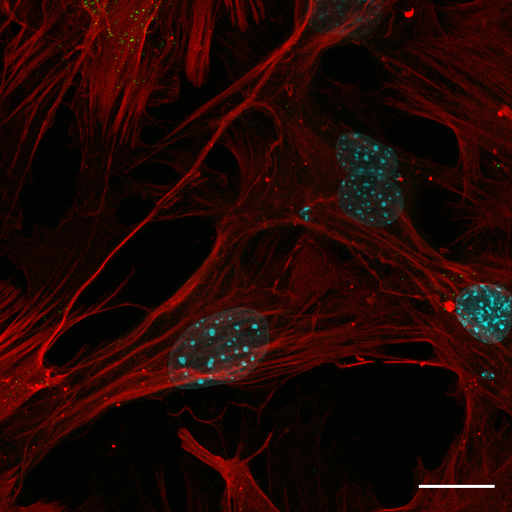

Supplement: Supplementary file 8 — Source data Fig. 6 [file 44319_2024_356_MOESM8_ESM.zip › Figure 6/6A/2_rTGFB/rTGFB_merge.png]

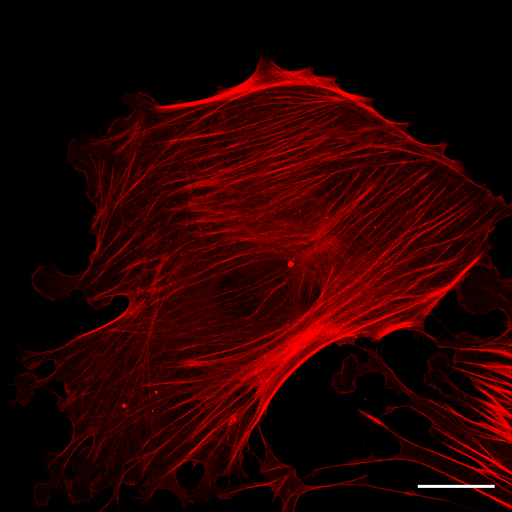

Supplement: Supplementary file 8 — Source data Fig. 6 [file 44319_2024_356_MOESM8_ESM.zip › Figure 6/6A/3_BEC_WT_CM/BEC_WT_CM_aSMA.png]

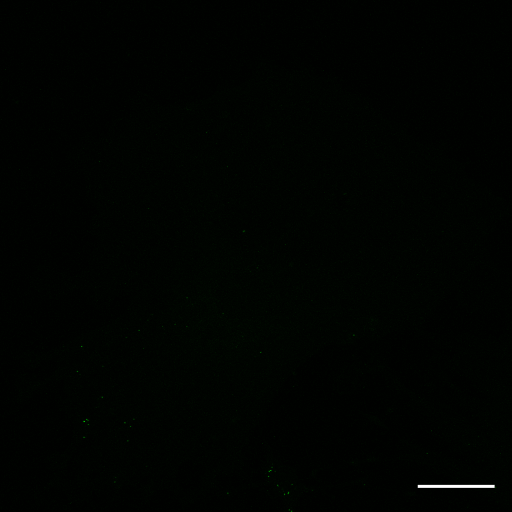

Supplement: Supplementary file 8 — Source data Fig. 6 [file 44319_2024_356_MOESM8_ESM.zip › Figure 6/6A/3_BEC_WT_CM/BEC_WT_CM_BODIPY.png]

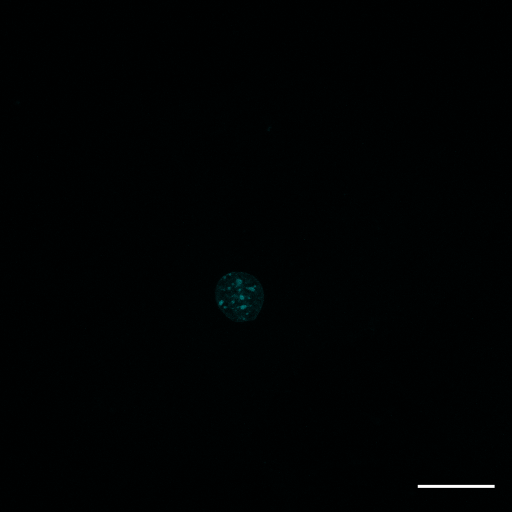

Supplement: Supplementary file 8 — Source data Fig. 6 [file 44319_2024_356_MOESM8_ESM.zip › Figure 6/6A/3_BEC_WT_CM/BEC_WT_CM_DAPI.png]

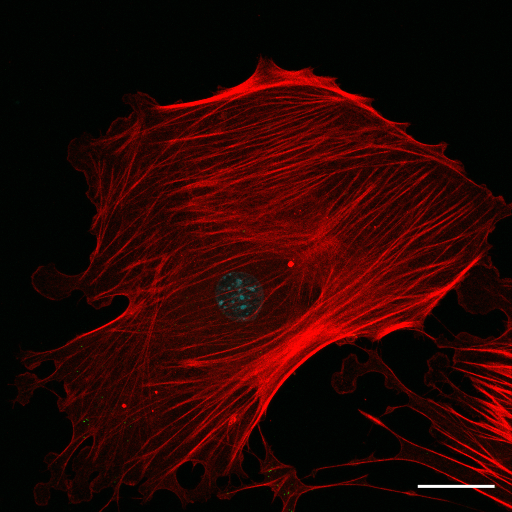

Supplement: Supplementary file 8 — Source data Fig. 6 [file 44319_2024_356_MOESM8_ESM.zip › Figure 6/6A/3_BEC_WT_CM/BEC_WT_CM_merge.png]

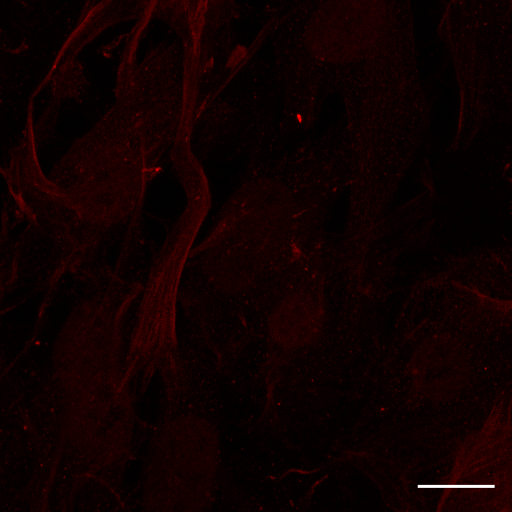

Supplement: Supplementary file 8 — Source data Fig. 6 [file 44319_2024_356_MOESM8_ESM.zip › Figure 6/6A/4_BEC_KO_CM/BEC_KO_CM_aSMA.png]

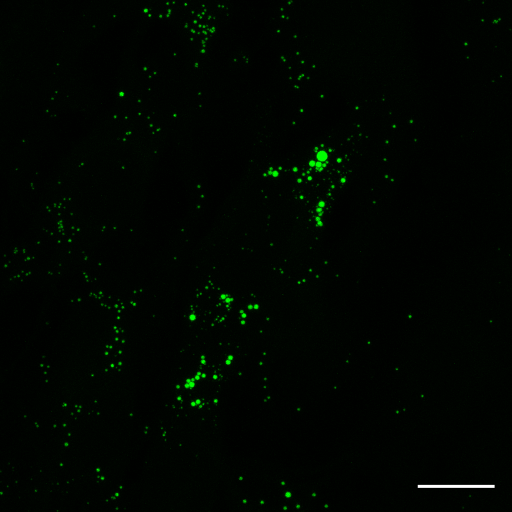

Supplement: Supplementary file 8 — Source data Fig. 6 [file 44319_2024_356_MOESM8_ESM.zip › Figure 6/6A/4_BEC_KO_CM/BEC_KO_CM_BODIPY.png]

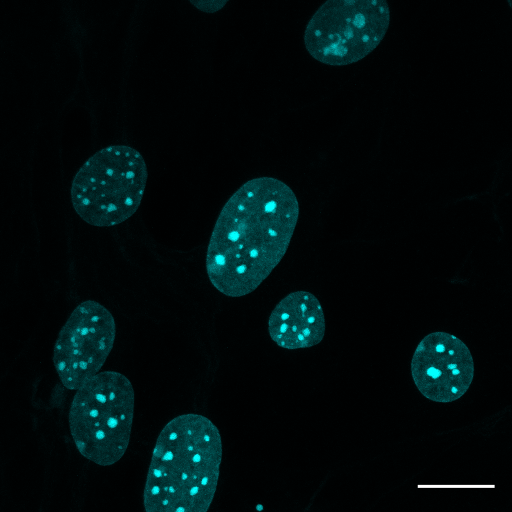

Supplement: Supplementary file 8 — Source data Fig. 6 [file 44319_2024_356_MOESM8_ESM.zip › Figure 6/6A/4_BEC_KO_CM/BEC_KO_CM_DAPI.png]

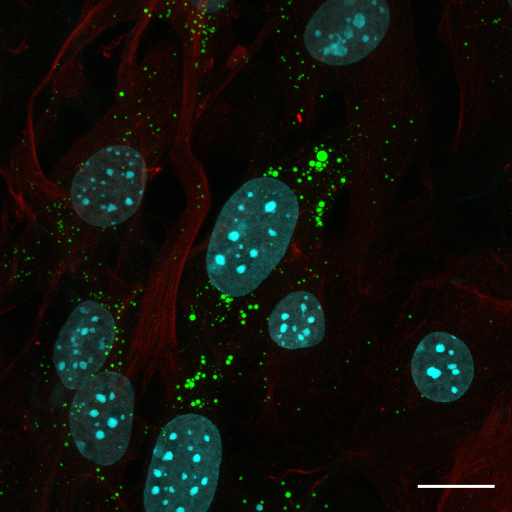

Supplement: Supplementary file 8 — Source data Fig. 6 [file 44319_2024_356_MOESM8_ESM.zip › Figure 6/6A/4_BEC_KO_CM/BEC_KO_CM_merge.png]

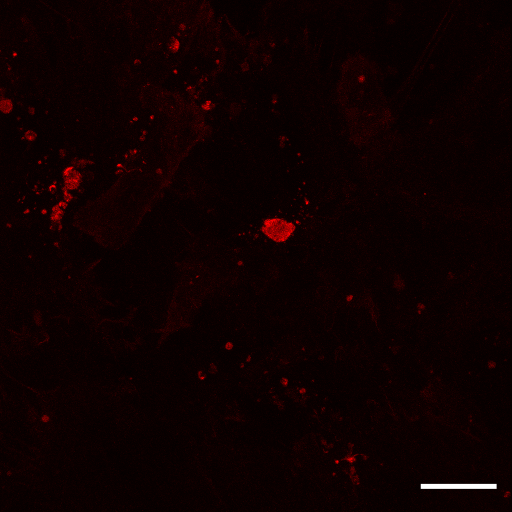

Supplement: Supplementary file 9 — Source data Fig. 7 [file 44319_2024_356_MOESM9_ESM.zip › Figure 7/7C/Ctrl_z1_aSMA.tif]

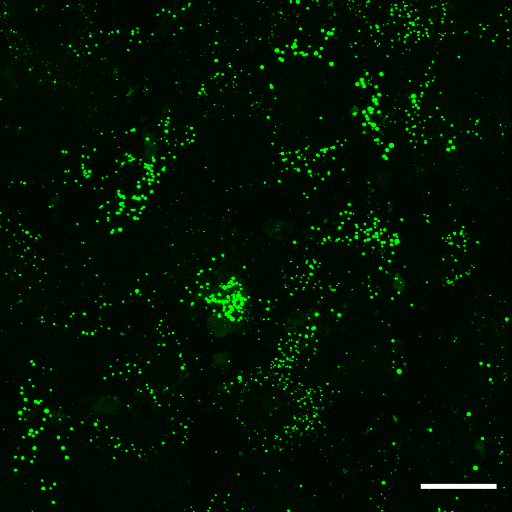

Supplement: Supplementary file 9 — Source data Fig. 7 [file 44319_2024_356_MOESM9_ESM.zip › Figure 7/7C/Ctrl_z1_BODIPY.tif]

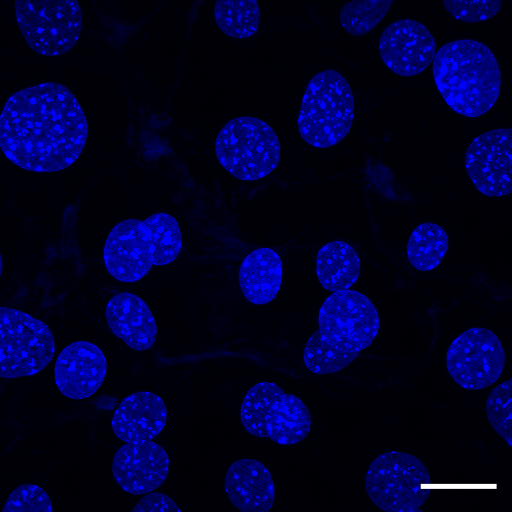

Supplement: Supplementary file 9 — Source data Fig. 7 [file 44319_2024_356_MOESM9_ESM.zip › Figure 7/7C/Ctrl_z1_DAPI.tif]

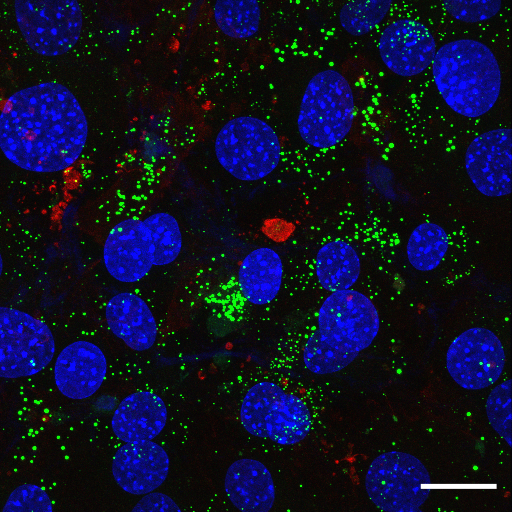

Supplement: Supplementary file 9 — Source data Fig. 7 [file 44319_2024_356_MOESM9_ESM.zip › Figure 7/7C/Ctrl_z1_merge.tif]

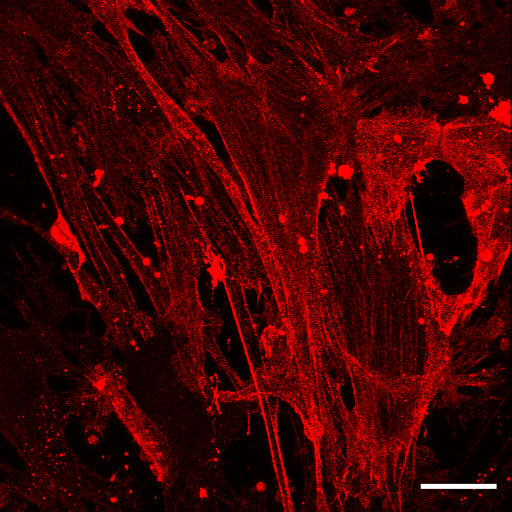

Supplement: Supplementary file 9 — Source data Fig. 7 [file 44319_2024_356_MOESM9_ESM.zip › Figure 7/7C/Jag1_z1_aSMA.tif]

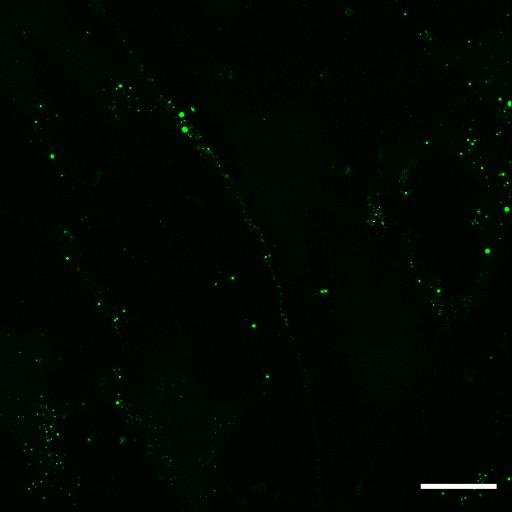

Supplement: Supplementary file 9 — Source data Fig. 7 [file 44319_2024_356_MOESM9_ESM.zip › Figure 7/7C/Jag1_z1_BODIPY.tif]

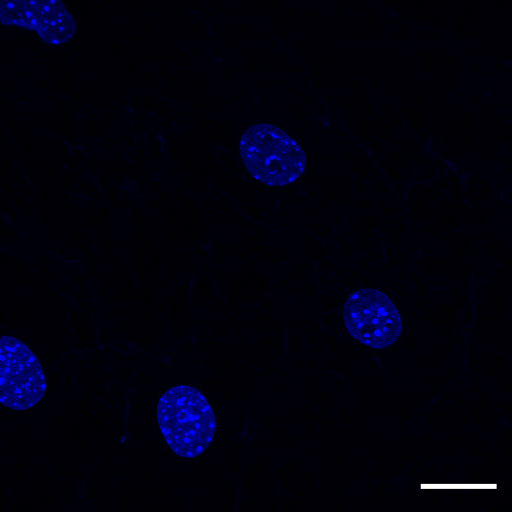

Supplement: Supplementary file 9 — Source data Fig. 7 [file 44319_2024_356_MOESM9_ESM.zip › Figure 7/7C/Jag1_z1_DAPI.tif]

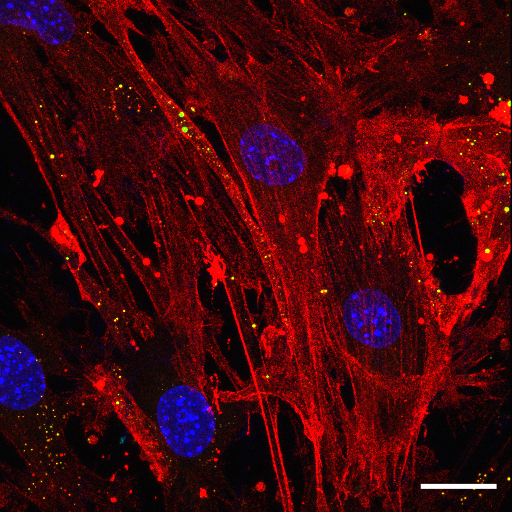

Supplement: Supplementary file 9 — Source data Fig. 7 [file 44319_2024_356_MOESM9_ESM.zip › Figure 7/7C/Jag1_z1_merge.tif]

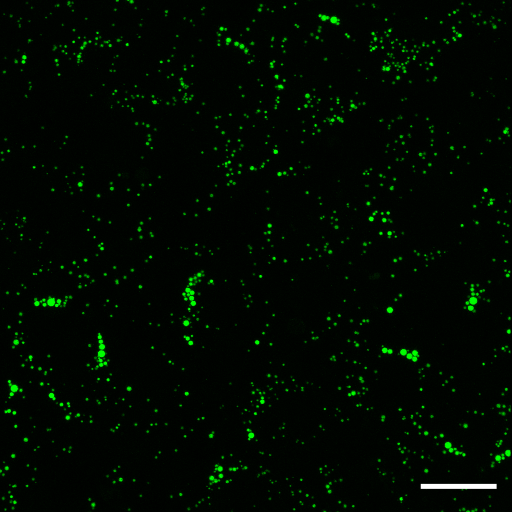

Supplement: Supplementary file 10 — Source data Fig. 8 [file 44319_2024_356_MOESM10_ESM.zip › Figure 8/8C/1_Control/Plain_1-BODIPY.tif]

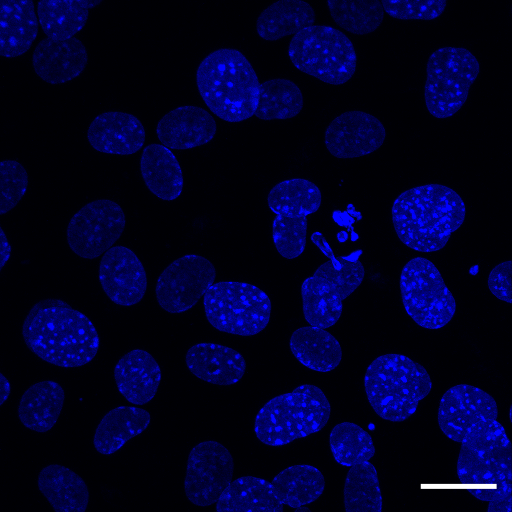

Supplement: Supplementary file 10 — Source data Fig. 8 [file 44319_2024_356_MOESM10_ESM.zip › Figure 8/8C/1_Control/Plain_1-DAPI.tif]

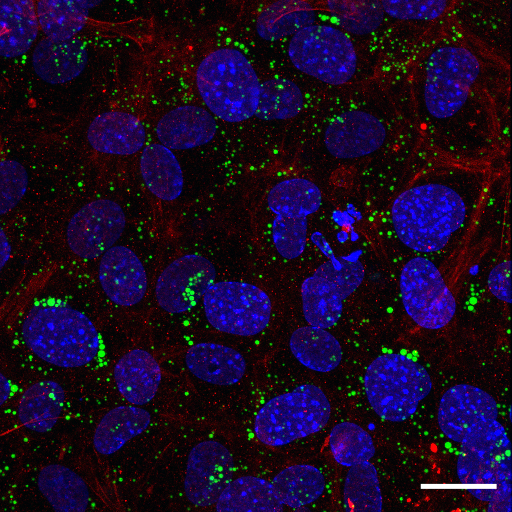

Supplement: Supplementary file 10 — Source data Fig. 8 [file 44319_2024_356_MOESM10_ESM.zip › Figure 8/8C/1_Control/Plain_1-merge.tif]

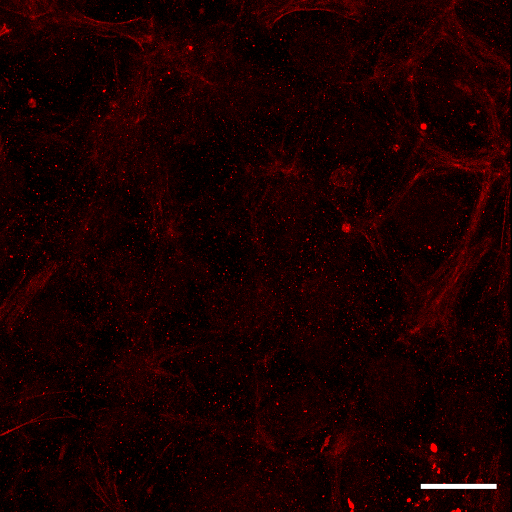

Supplement: Supplementary file 10 — Source data Fig. 8 [file 44319_2024_356_MOESM10_ESM.zip › Figure 8/8C/1_Control/Plain_1-SMA.tif]

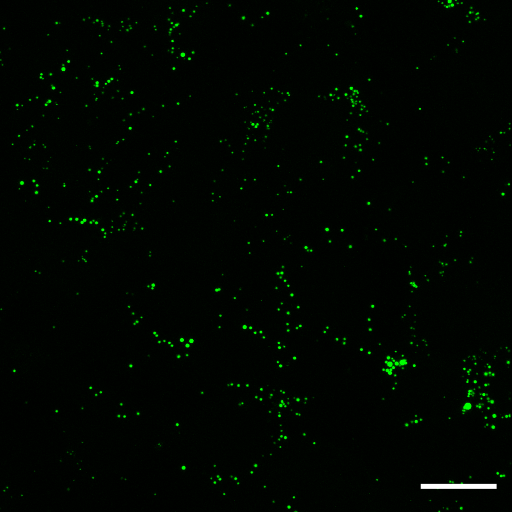

Supplement: Supplementary file 10 — Source data Fig. 8 [file 44319_2024_356_MOESM10_ESM.zip › Figure 8/8C/2_BEC Rage WT CM/EV_ctrl_1_BODIPY.tif]

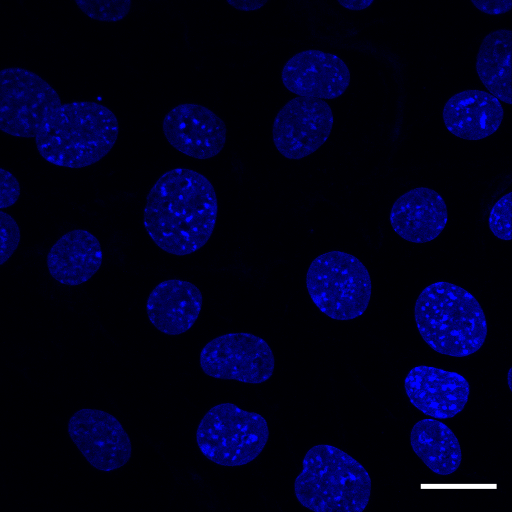

Supplement: Supplementary file 10 — Source data Fig. 8 [file 44319_2024_356_MOESM10_ESM.zip › Figure 8/8C/2_BEC Rage WT CM/EV_ctrl_1_DAPI.tif]

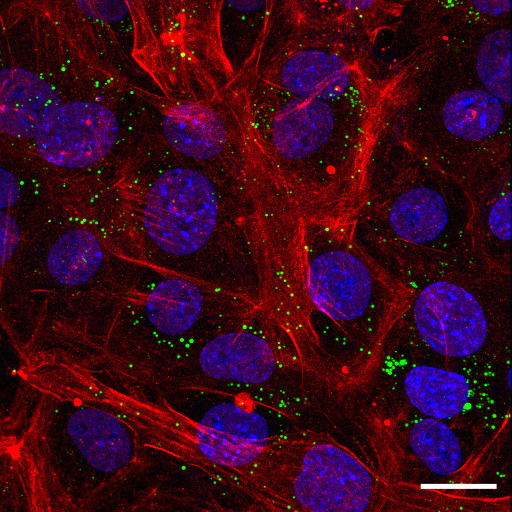

Supplement: Supplementary file 10 — Source data Fig. 8 [file 44319_2024_356_MOESM10_ESM.zip › Figure 8/8C/2_BEC Rage WT CM/EV_ctrl_1_Merge.tif]

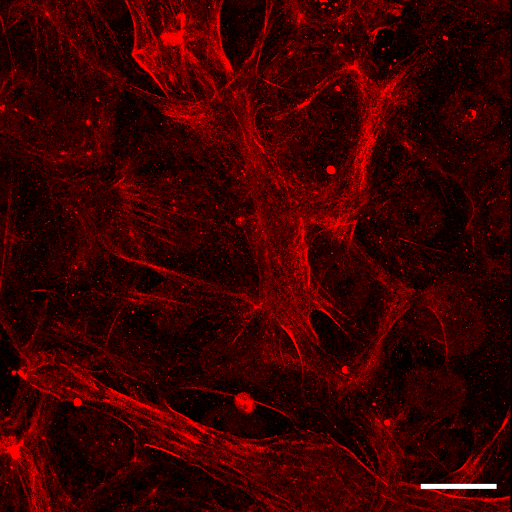

Supplement: Supplementary file 10 — Source data Fig. 8 [file 44319_2024_356_MOESM10_ESM.zip › Figure 8/8C/2_BEC Rage WT CM/EV_ctrl_1_SMA.tif]

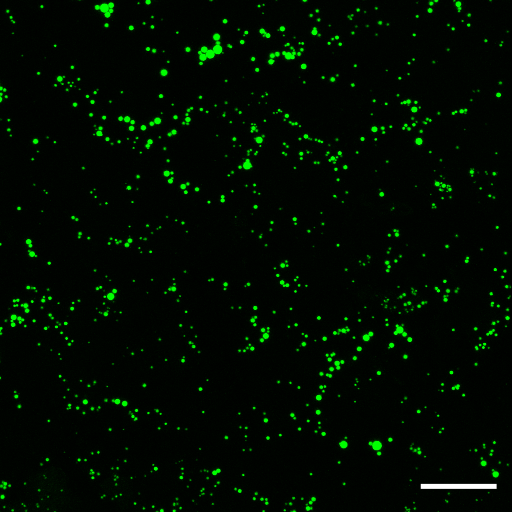

Supplement: Supplementary file 10 — Source data Fig. 8 [file 44319_2024_356_MOESM10_ESM.zip › Figure 8/8C/3_BEC Rage KO CM/cre_ctrl_1_BODIPY.tif]

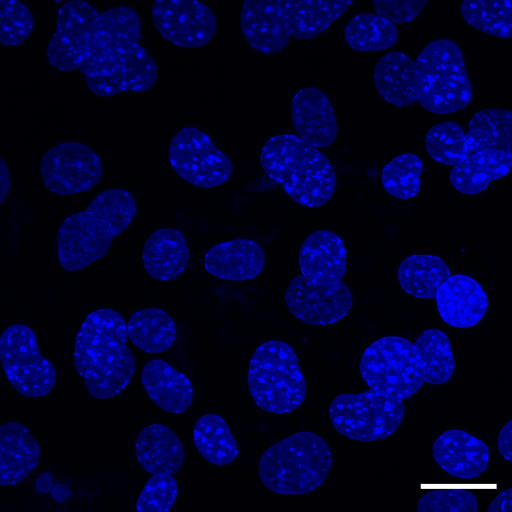

Supplement: Supplementary file 10 — Source data Fig. 8 [file 44319_2024_356_MOESM10_ESM.zip › Figure 8/8C/3_BEC Rage KO CM/cre_ctrl_1_DAPI.tif]

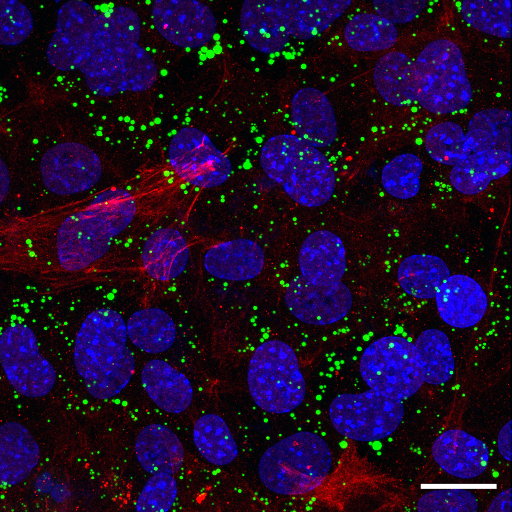

Supplement: Supplementary file 10 — Source data Fig. 8 [file 44319_2024_356_MOESM10_ESM.zip › Figure 8/8C/3_BEC Rage KO CM/cre_ctrl_1_Merge.tif]

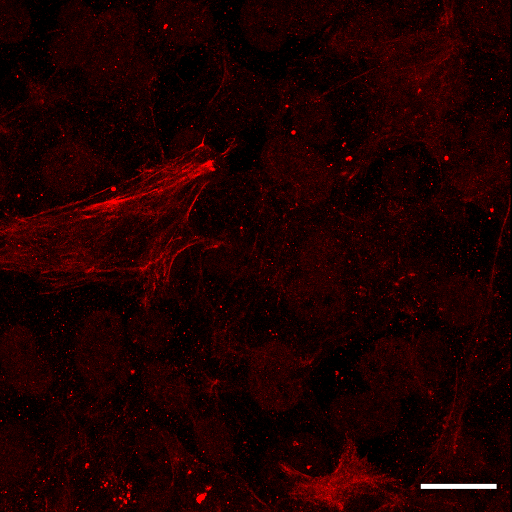

Supplement: Supplementary file 10 — Source data Fig. 8 [file 44319_2024_356_MOESM10_ESM.zip › Figure 8/8C/3_BEC Rage KO CM/cre_ctrl_1_SMA.tif]

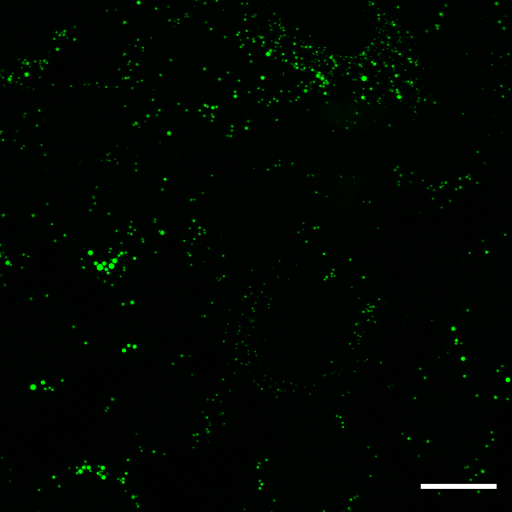

Supplement: Supplementary file 10 — Source data Fig. 8 [file 44319_2024_356_MOESM10_ESM.zip › Figure 8/8C/4_BEC Rage WT+siNegative CM/siNEG_2_BODIPY.tif]

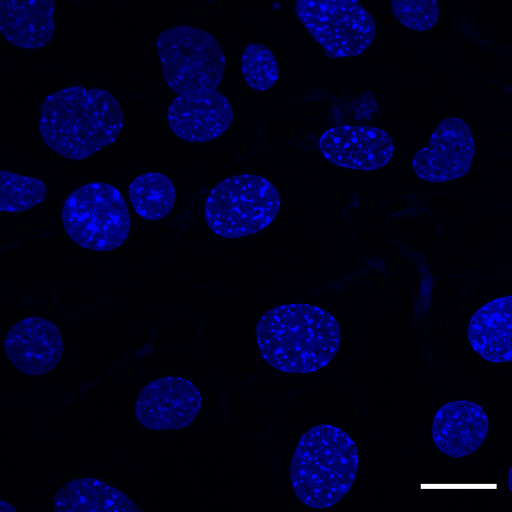

Supplement: Supplementary file 10 — Source data Fig. 8 [file 44319_2024_356_MOESM10_ESM.zip › Figure 8/8C/4_BEC Rage WT+siNegative CM/siNEG_2_DAPI.tif]

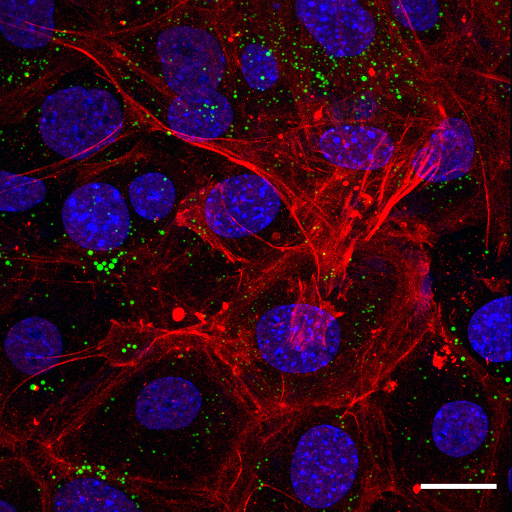

Supplement: Supplementary file 10 — Source data Fig. 8 [file 44319_2024_356_MOESM10_ESM.zip › Figure 8/8C/4_BEC Rage WT+siNegative CM/siNEG_2_Merge.tif]

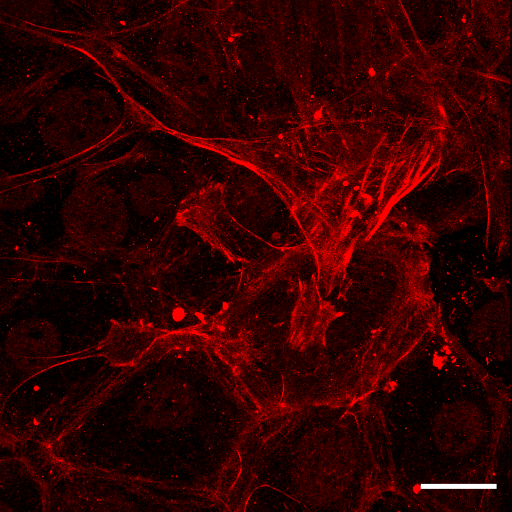

Supplement: Supplementary file 10 — Source data Fig. 8 [file 44319_2024_356_MOESM10_ESM.zip › Figure 8/8C/4_BEC Rage WT+siNegative CM/siNEG_2_SMA.tif]

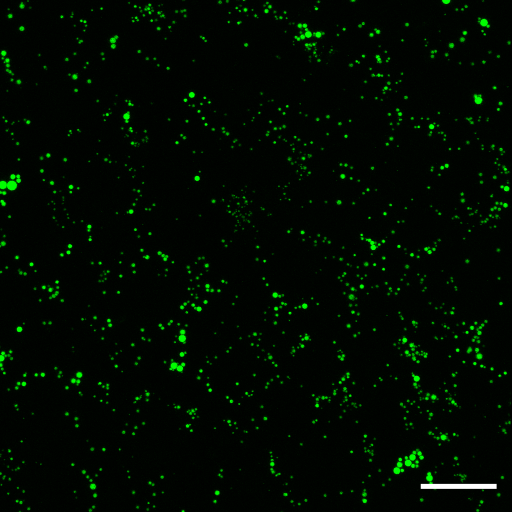

Supplement: Supplementary file 10 — Source data Fig. 8 [file 44319_2024_356_MOESM10_ESM.zip › Figure 8/8C/5_BEC Rage WT+siJAG1#1 CM/siJAG1_68530_1_BODIPY.tif]

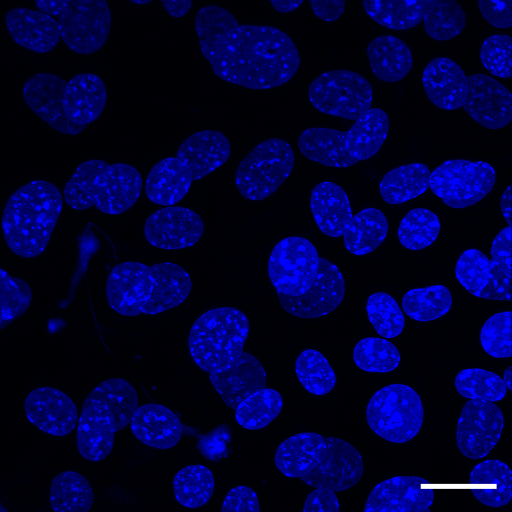

Supplement: Supplementary file 10 — Source data Fig. 8 [file 44319_2024_356_MOESM10_ESM.zip › Figure 8/8C/5_BEC Rage WT+siJAG1#1 CM/siJAG1_68530_1_DAPI.tif]

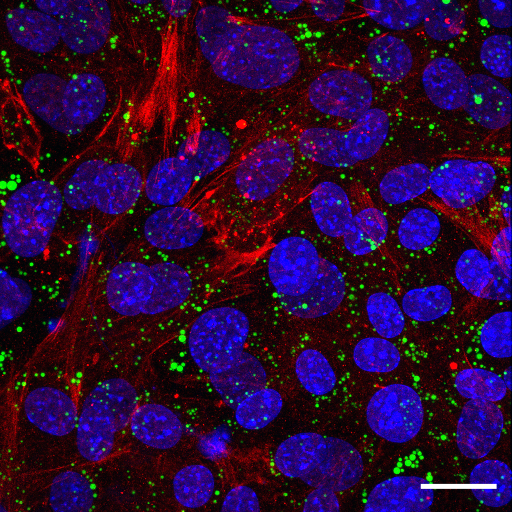

Supplement: Supplementary file 10 — Source data Fig. 8 [file 44319_2024_356_MOESM10_ESM.zip › Figure 8/8C/5_BEC Rage WT+siJAG1#1 CM/siJAG1_68530_1_Merge.tif]

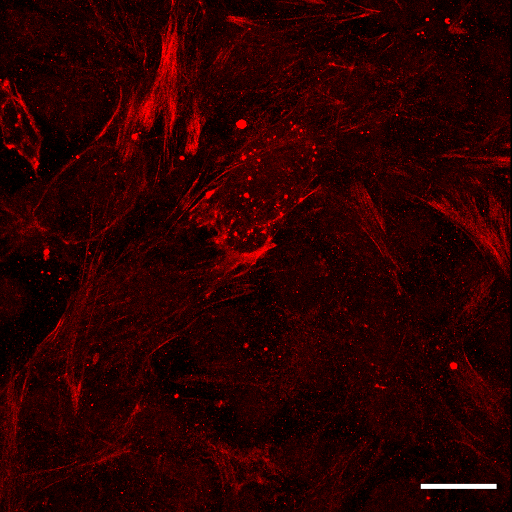

Supplement: Supplementary file 10 — Source data Fig. 8 [file 44319_2024_356_MOESM10_ESM.zip › Figure 8/8C/5_BEC Rage WT+siJAG1#1 CM/siJAG1_68530_1_SMA.tif]

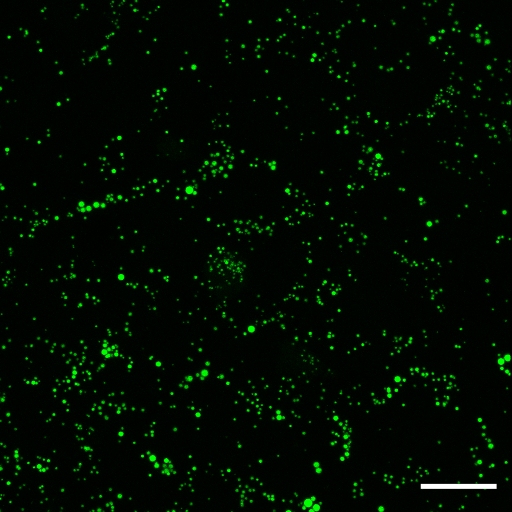

Supplement: Supplementary file 10 — Source data Fig. 8 [file 44319_2024_356_MOESM10_ESM.zip › Figure 8/8C/6_BEC Rage WT+siJag1#2 CM/siJag1_68532_2_BODIPY.tif]

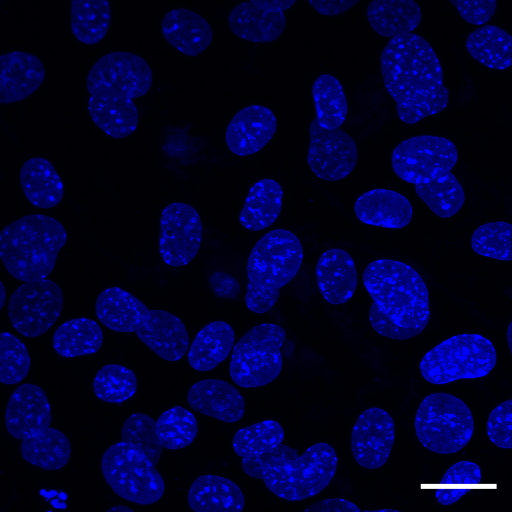

Supplement: Supplementary file 10 — Source data Fig. 8 [file 44319_2024_356_MOESM10_ESM.zip › Figure 8/8C/6_BEC Rage WT+siJag1#2 CM/siJag1_68532_2_DAPI.tif]

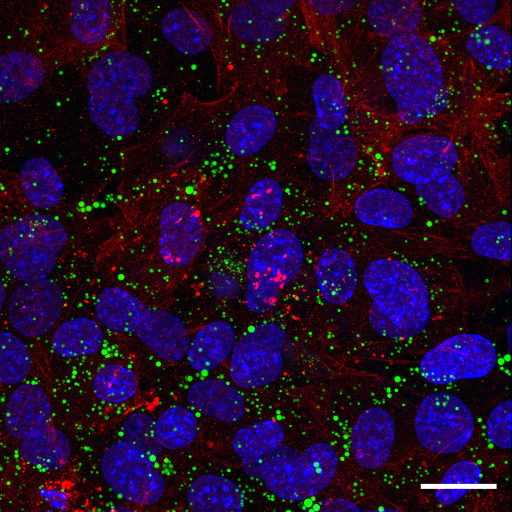

Supplement: Supplementary file 10 — Source data Fig. 8 [file 44319_2024_356_MOESM10_ESM.zip › Figure 8/8C/6_BEC Rage WT+siJag1#2 CM/siJag1_68532_2_Merge.tif]

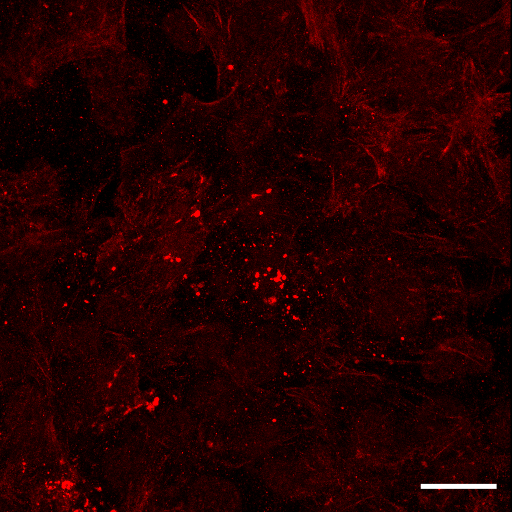

Supplement: Supplementary file 10 — Source data Fig. 8 [file 44319_2024_356_MOESM10_ESM.zip › Figure 8/8C/6_BEC Rage WT+siJag1#2 CM/siJag1_68532_2_SMA.tif]

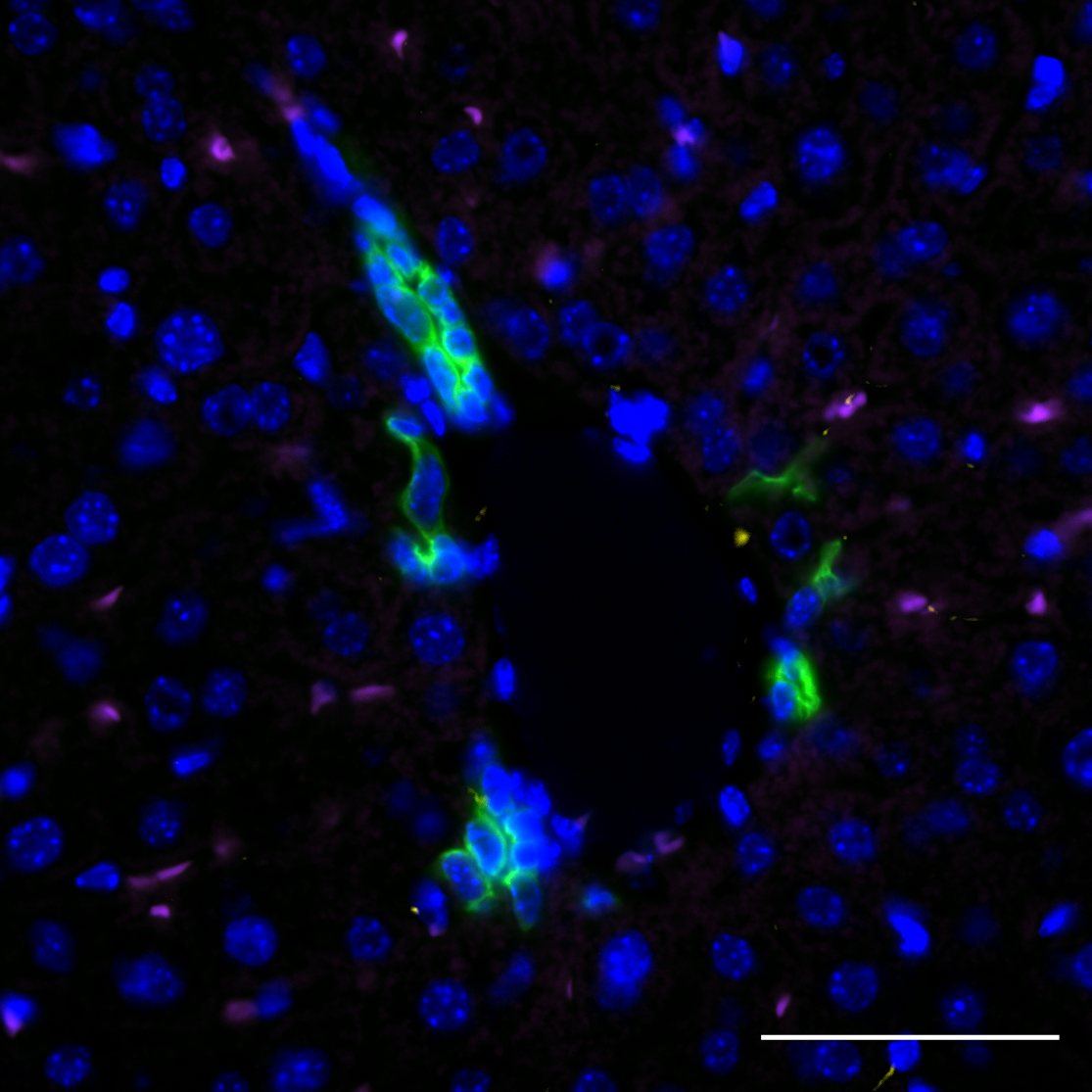

Supplement: Supplementary file 11 — Source data Fig. 9 [file 44319_2024_356_MOESM11_ESM.zip › Figure 9/1_ND_WT_m170_19-Merge.tif]

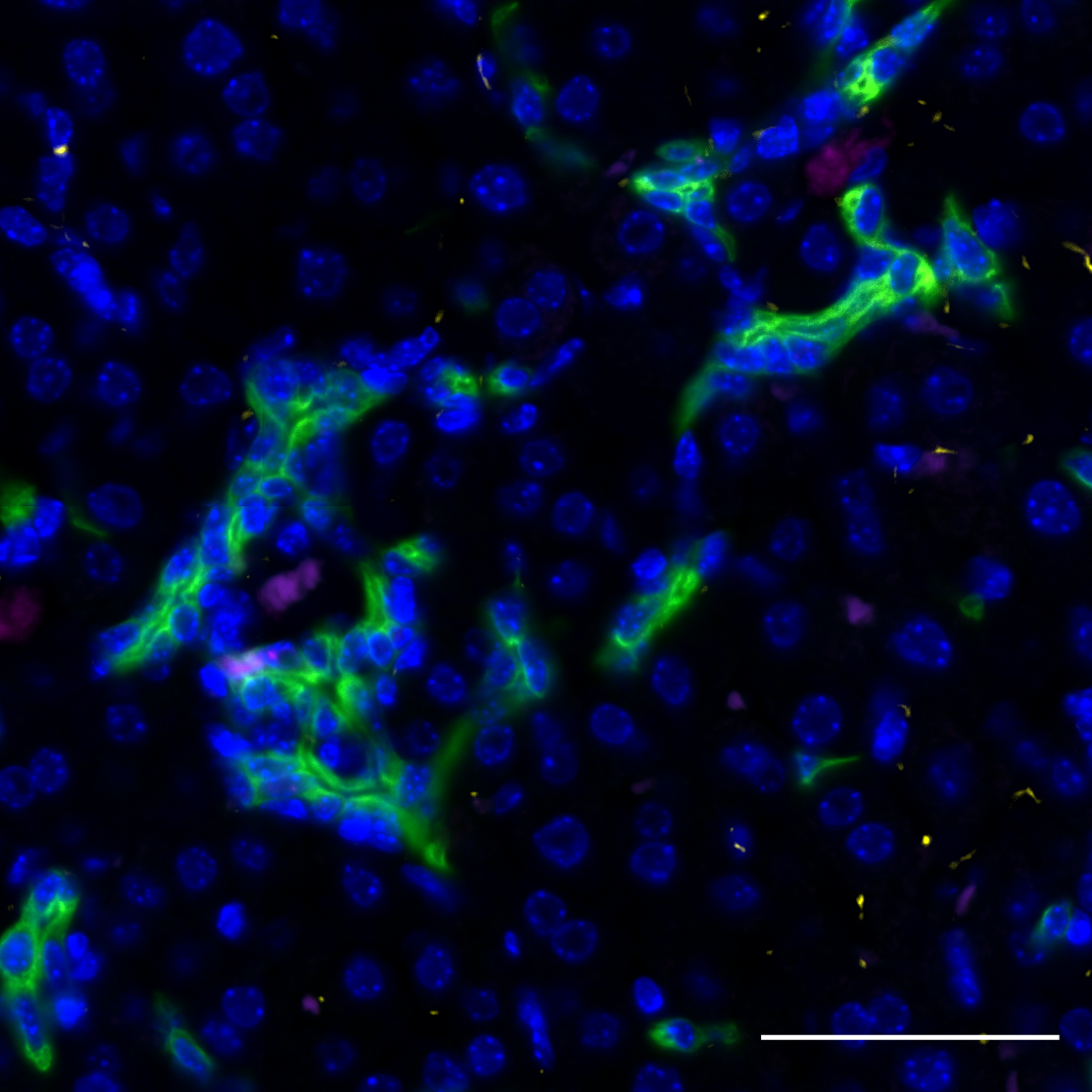

Supplement: Supplementary file 11 — Source data Fig. 9 [file 44319_2024_356_MOESM11_ESM.zip › Figure 9/2_ND_Het_m468_19-Merge.tif]

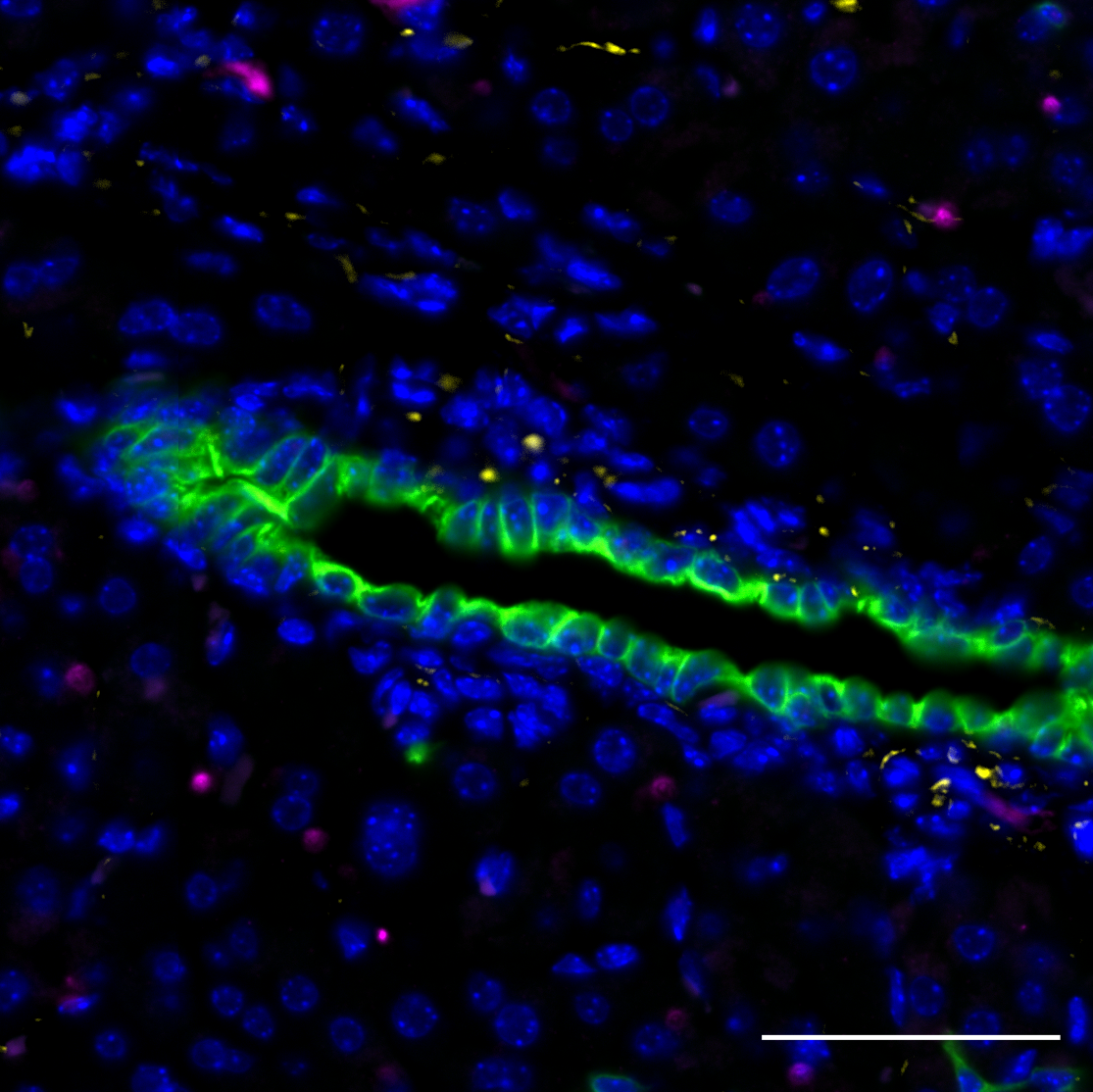

Supplement: Supplementary file 11 — Source data Fig. 9 [file 44319_2024_356_MOESM11_ESM.zip › Figure 9/3_ND_KO_m464_19-Merge.tif]
