# Supplementary material for: RAGE is a key regulator of ductular reaction-mediated fibrosis during cholestasis
Source: EMBO Rep. 2025 Jan 2;26(3):880–907. doi: 10.1038/s44319-024-00356-7 (PMC11811172; doi:10.1038/s44319-024-00356-7)
Supplement: Supplementary file 4 — Source data Fig. 2 [file 44319_2024_356_MOESM4_ESM.zip › Figure 2/2B/Batch 1/SD_2018-12-12.pdf]

BD FACSDiva 8.0.2

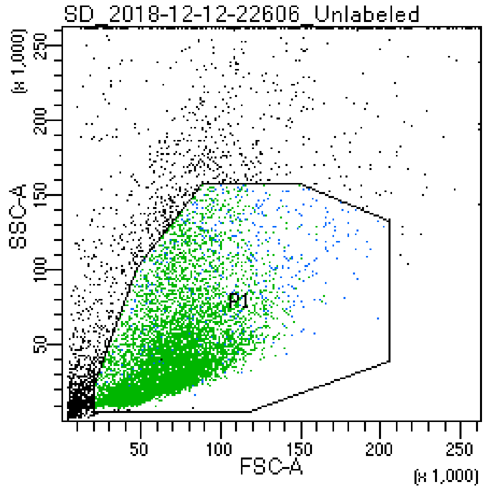

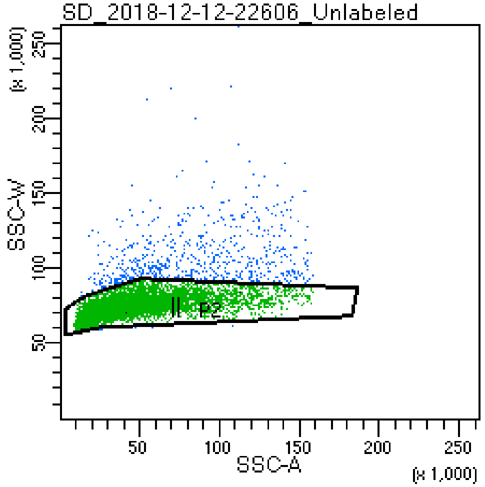

Tube: 22606_Unlabeled

Population	#Events	%Parent	%Total
All Events	10,000	####	100.0
P1	8,025	80.2	80.2
P2	7,369	91.8	73.7
P3	0	0.0	0.0
P4	0	0.0	0.0
P5	2	0.0	0.0

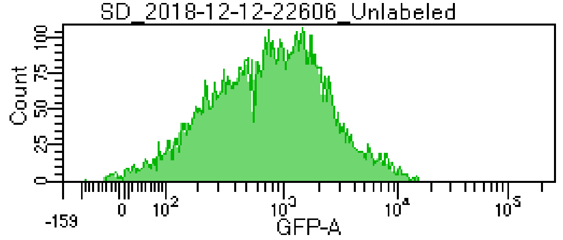

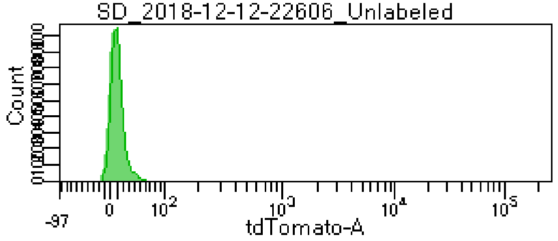

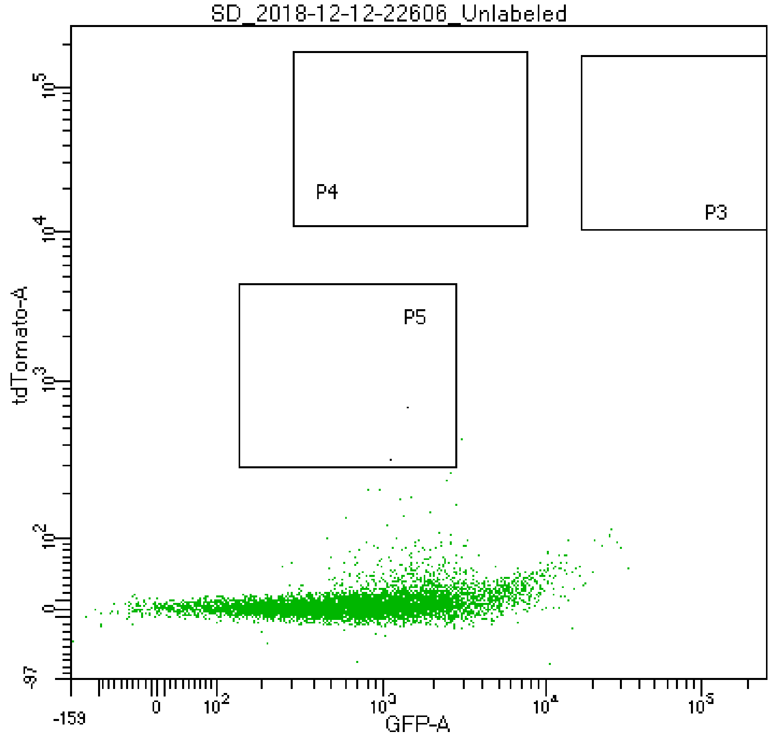

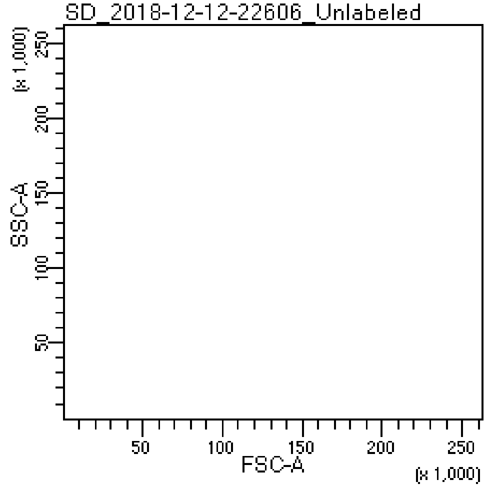

Sort_2018-12-12

22607
P4: 444.000
P5: 65.900

22411
P4: 98.100
P3: 282.800

BD FACSDiva 8.0.2

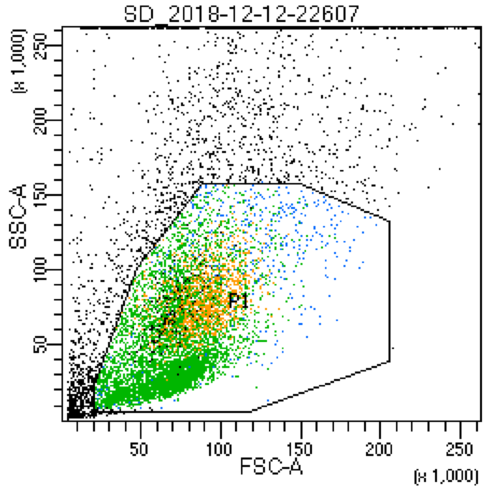

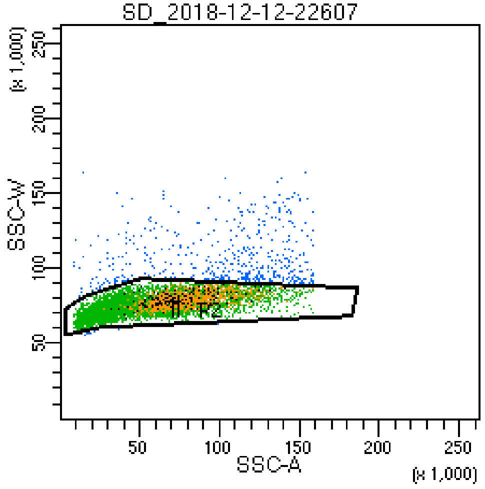

Tube: 22607

Population	#Events	%Parent	%Total
All Events	10,000	####	100.0
P1	7,663	76.6	76.6
P2	7,167	93.5	71.7
P3	0	0.0	0.0
P4	1,058	14.8	10.6
P5	165	2.3	1.7

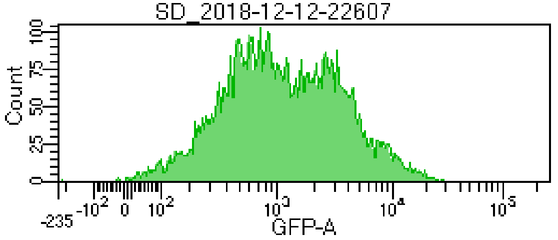

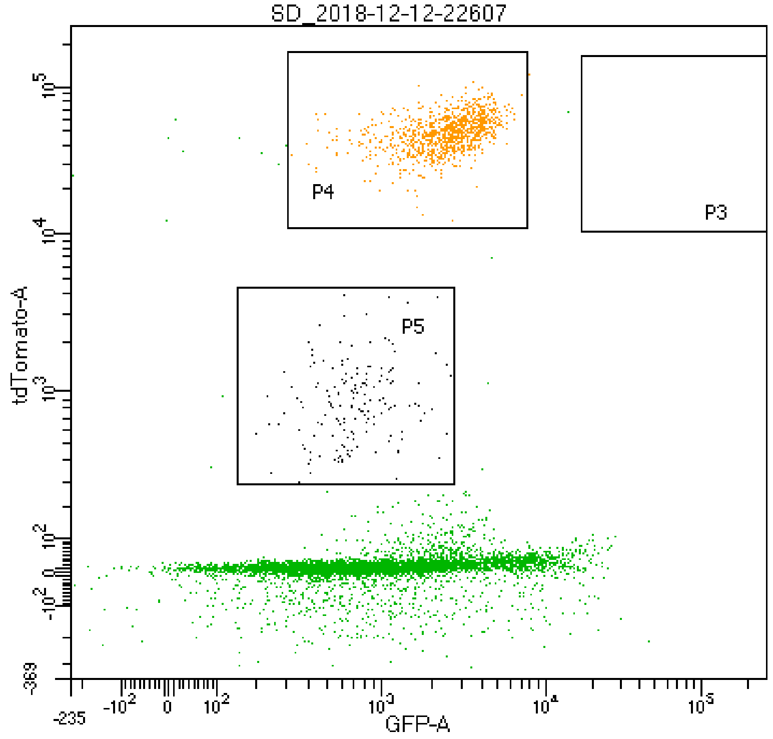

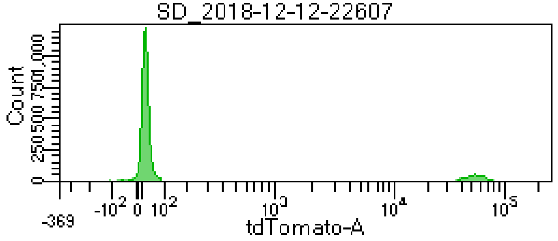

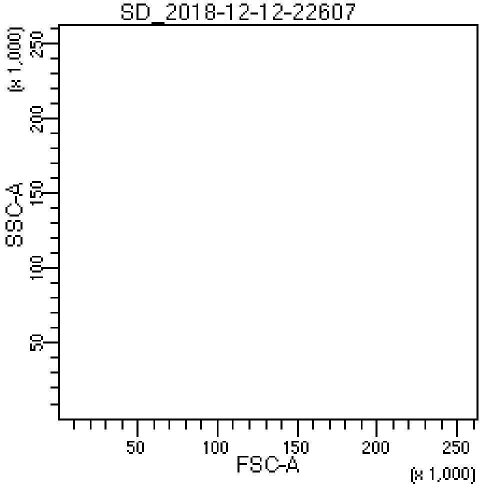

Sort_2018-12-12

	**22607**
**P4:**	**444.000**
**P5:**	**65.900**
	**22411**
**P4:**	**98.100**
**P3:**	**282.800**

BD FACSDiva 8.0.2

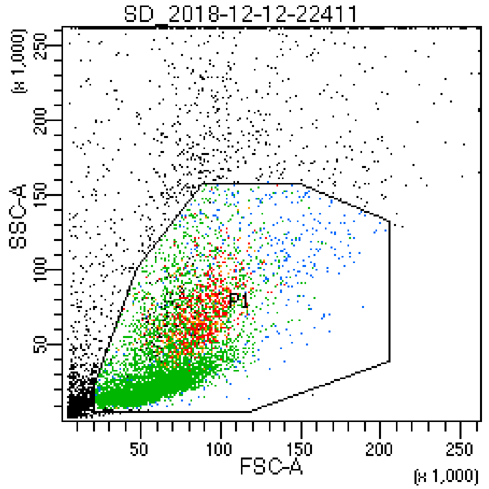

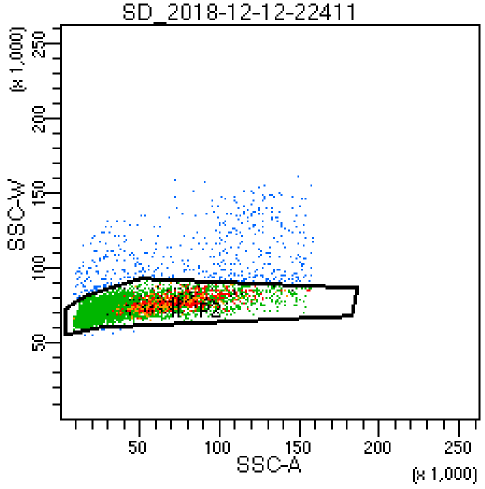

Tube: 22411

Population	#Events	%Parent	%Total
All Events	10,000	####	100.0
P1	7,619	76.2	76.2
P2	7,111	93.3	71.1
P3	550	7.7	5.5
P4	191	2.7	1.9
P5	92	1.3	0.9

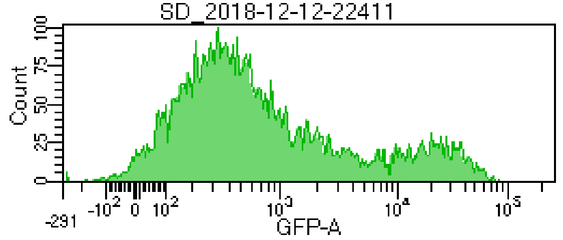

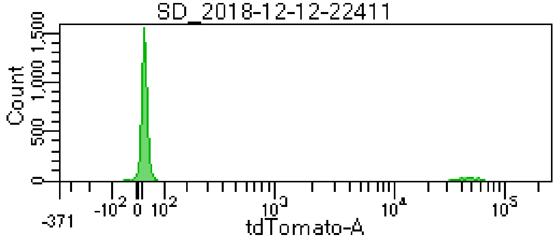

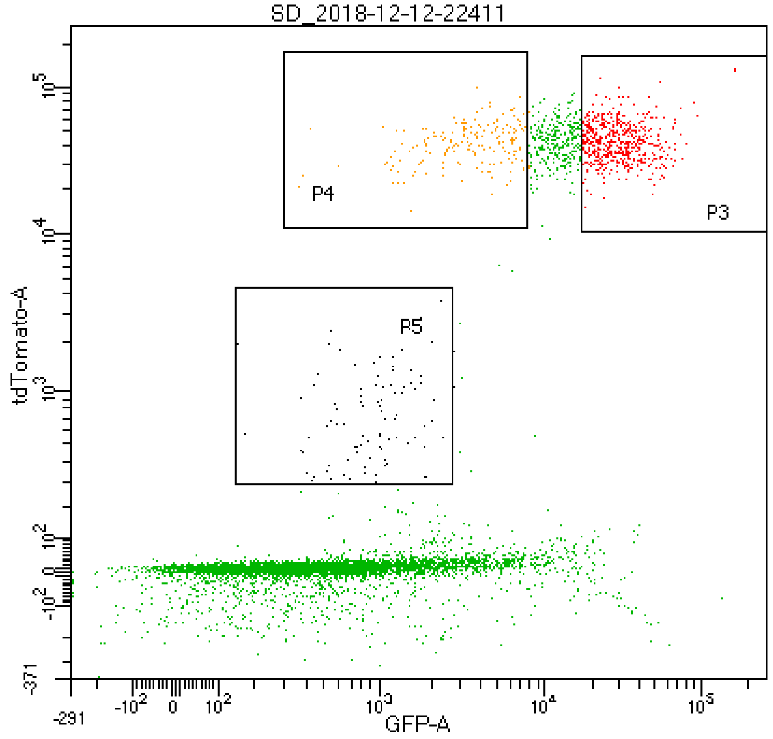

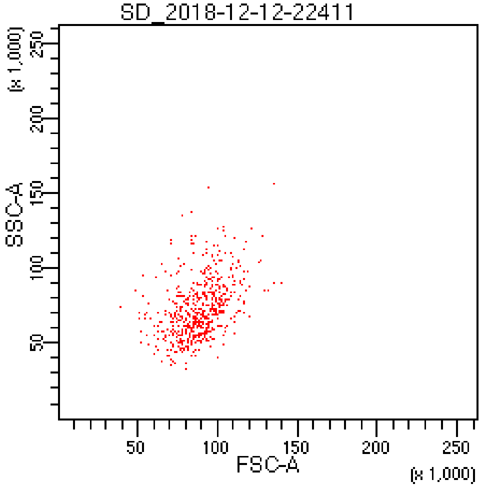

Sort_2018-12-12

	**22607**
**P4:**	**444.000**
**P5:**	**65.900**
	**22411**
**P4:**	**98.100**
**P3:**	**282.800**
