# Supplementary material for: RAGE is a key regulator of ductular reaction-mediated fibrosis during cholestasis
Source: EMBO Rep. 2025 Jan 2;26(3):880–907. doi: 10.1038/s44319-024-00356-7 (PMC11811172; doi:10.1038/s44319-024-00356-7)
Supplement: Supplementary file 4 — Source data Fig. 2 [file 44319_2024_356_MOESM4_ESM.zip › Figure 2/2B/Batch 2/20191103 DS.pdf]

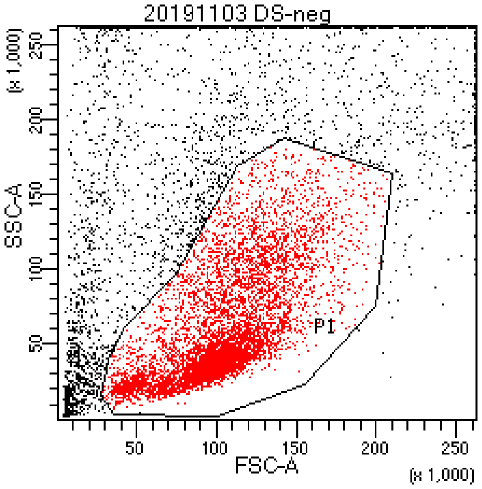

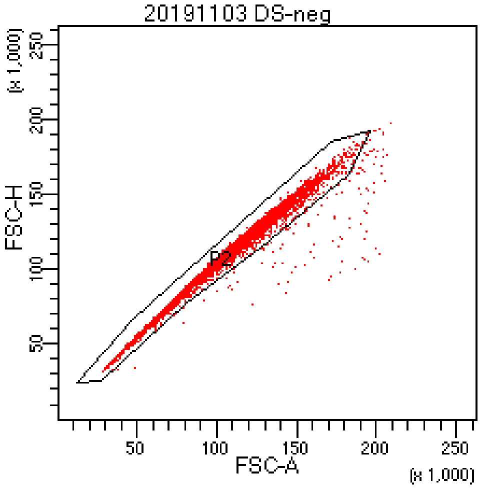

Tube: neg

Population	#Events	%Parent	%Total
All Events	10,000	####	100.0
P1	6,852	68.5	68.5
P2	6,718	98.0	67.2
tdtomato	0	0.0	0.0
tdtomato+GFP	0	0.0	0.0

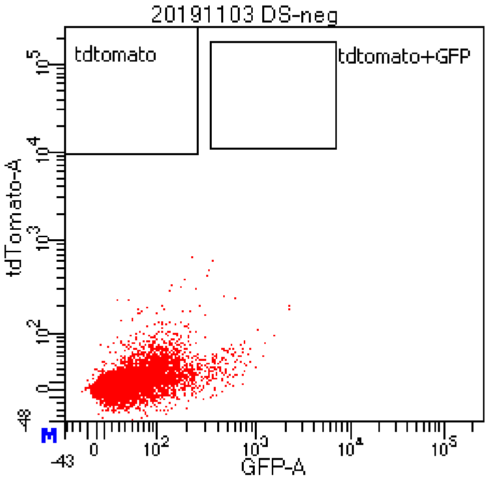

	tdtomato	tdtomato+GFP
tdtomato	124.500	
GFP+tdtomato	43.500	173.400

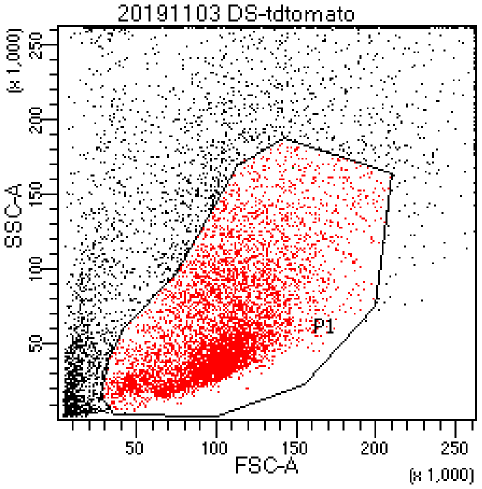

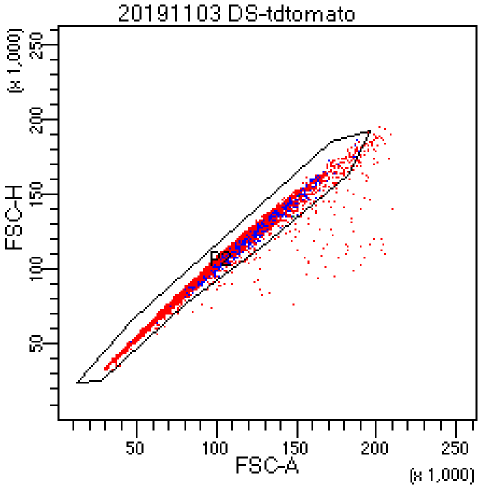

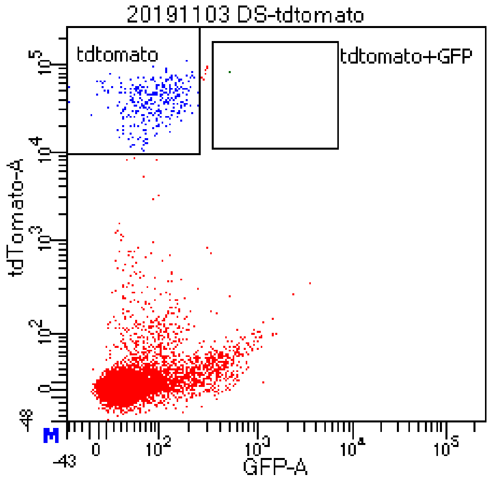

Tube: tdtomato

Population	#Events	%Parent	%Total
All Events	10,000	####	100.0
P1	6,616	66.2	66.2
P2	6,464	97.7	64.6
tdtomato	316	4.9	3.2
tdtomato+GFP	2	0.0	0.0

	tdtomato	tdtomato+GFP
tdtomato	124.500	
GFP+tdtomato	43.500	173.400

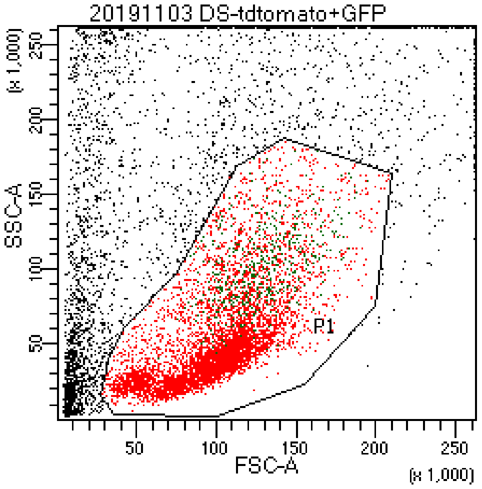

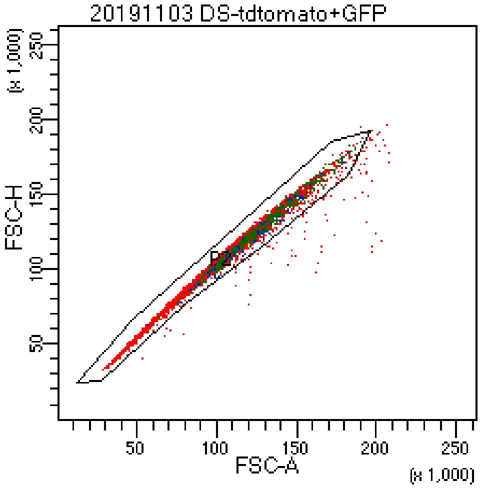

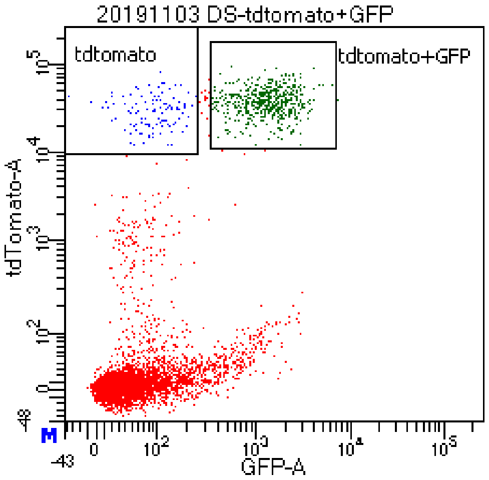

Tube: tdtomato+GFP

Population	#Events	%Parent	%Total
All Events	10,000	####	100.0
P1	6,085	60.8	60.8
P2	5,994	98.5	59.9
tdtomato	113	1.9	1.1
tdtomato+GFP	504	8.4	5.0

	tdtomato	tdtomato+GFP
tdtomato	124.500	
GFP+tdtomato	43.500	173.400
