# Supplementary material for: RAGE is a key regulator of ductular reaction-mediated fibrosis during cholestasis
Source: EMBO Rep. 2025 Jan 2;26(3):880–907. doi: 10.1038/s44319-024-00356-7 (PMC11811172; doi:10.1038/s44319-024-00356-7)
Supplement: Supplementary file 4 — Source data Fig. 2 [file 44319_2024_356_MOESM4_ESM.zip › Figure 2/2B/Batch 4/LM2019-07-29-Batch_Analysis_01082019135629.pdf]

Batch Analysis Report

Run Date: 8/1/19 1:59 PM
Experiment: LM2019-07-29
User ID: LamM
Statistics Output: N/A
Worksheet PDF Output: Z:\A100\Macrina\Sort 2019-0729\LM2019-07-29-Batch_Analysis_0108 2019135629.pdf

Specimen_001

Tube	Status	Run Time
unst	OK	8/1/19 1:59 PM
dtomato	OK	8/1/19 1:59 PM
sample	OK	8/1/19 1:59 PM

BD FACSDiva 8.0.2

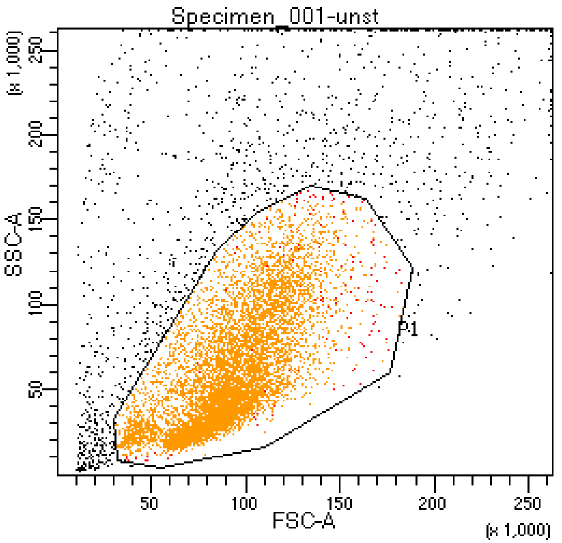

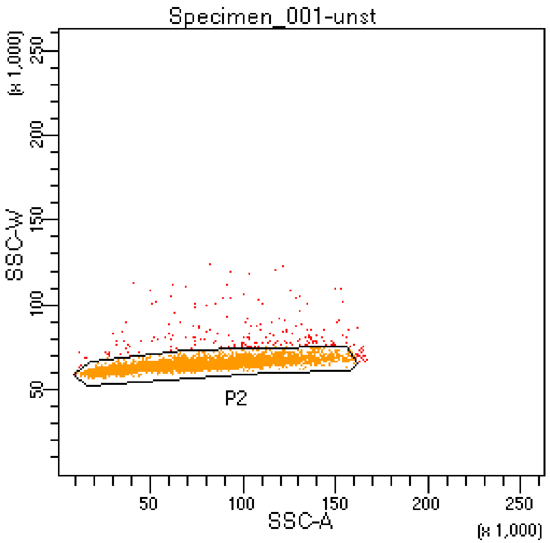

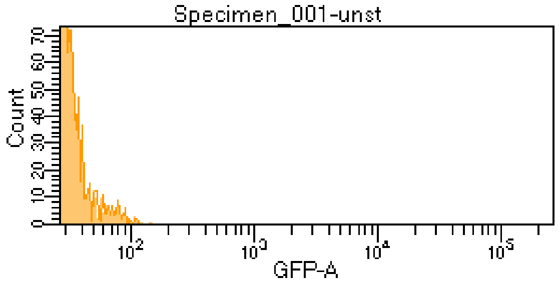

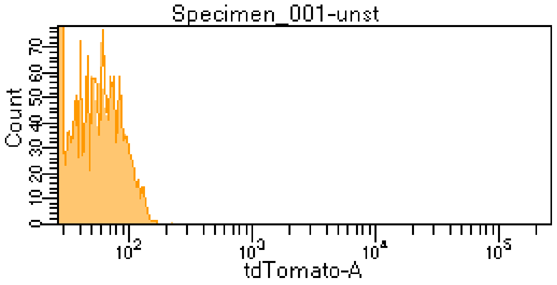

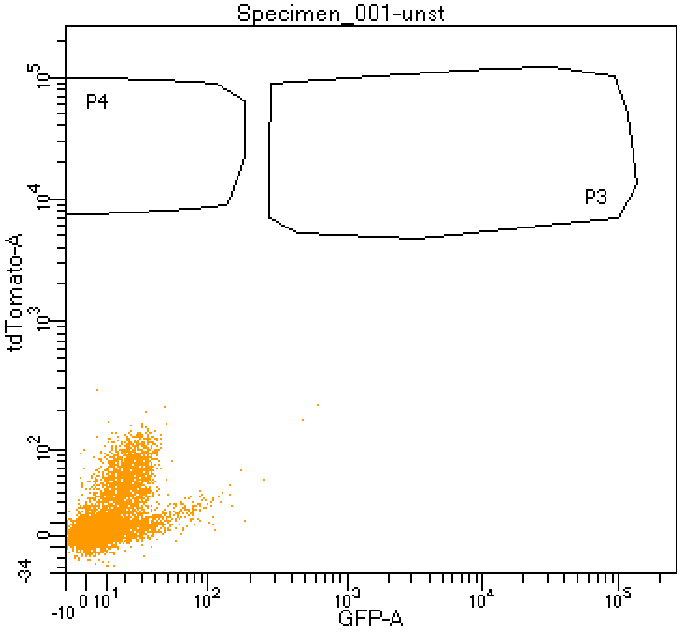

Tube: unst

Population	#Events	%Parent	%Total
All Events	10,000	####	100.0
P1	8,574	85.7	85.7
P2	8,369	97.6	83.7
P3	0	0.0	0.0
P4	0	0.0	0.0

BD FACSDiva 8.0.2

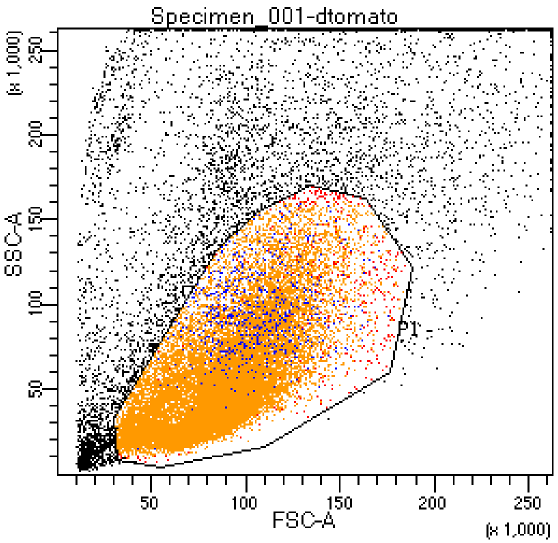

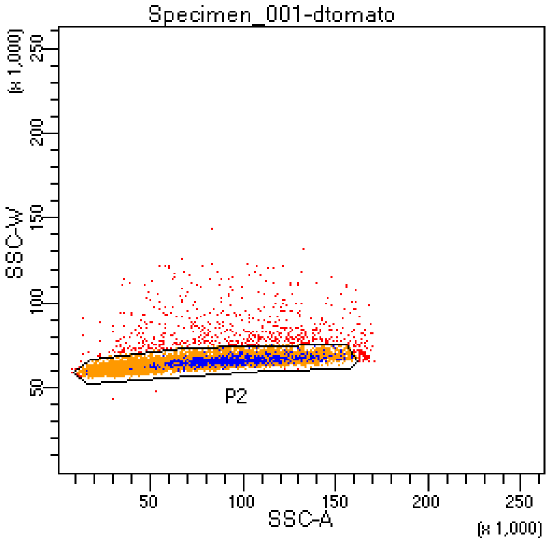

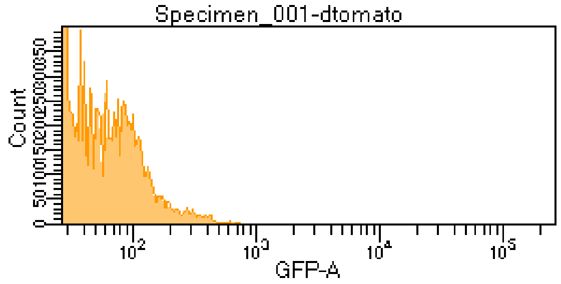

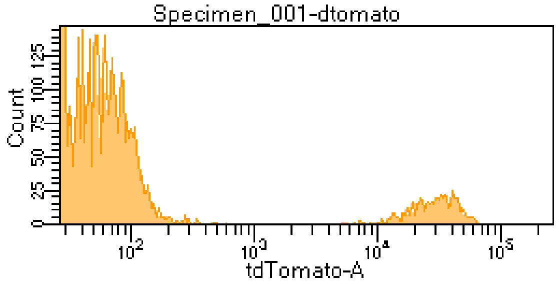

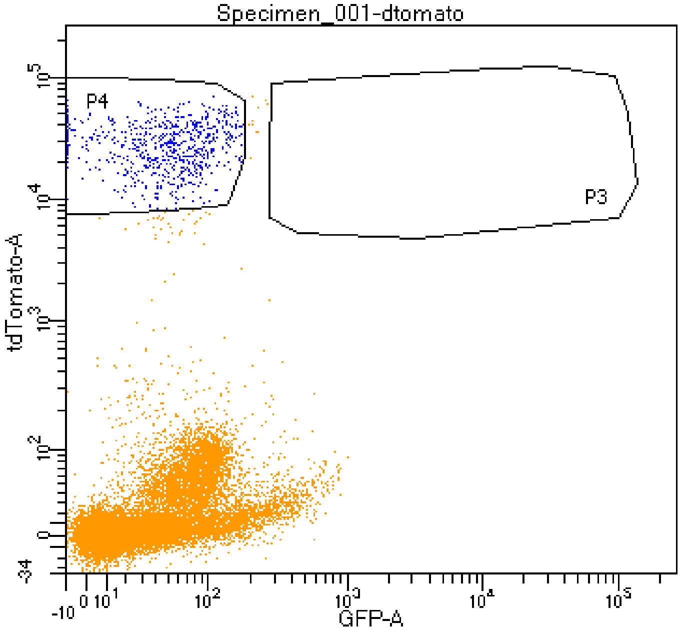

Tube: dtomato

Population	#Events	%Parent	%Total
All Events	30,000	####	100.0
P1	24,460	81.5	81.5
P2	23,794	97.3	79.3
P3	1	0.0	0.0
P4	671	2.8	2.2

BD FACSDiva 8.0.2

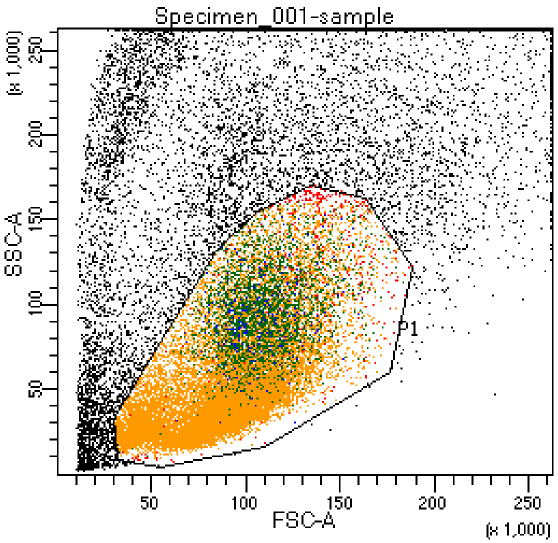

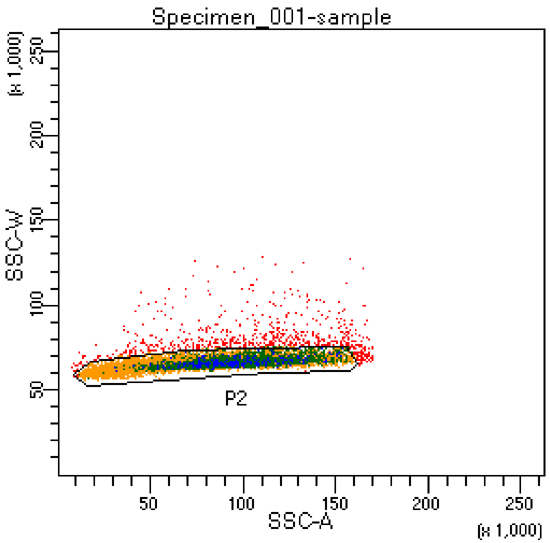

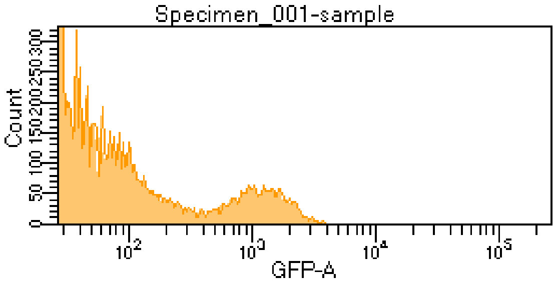

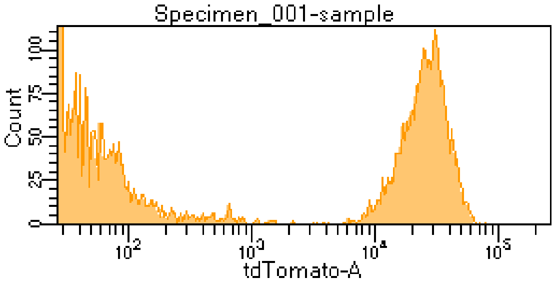

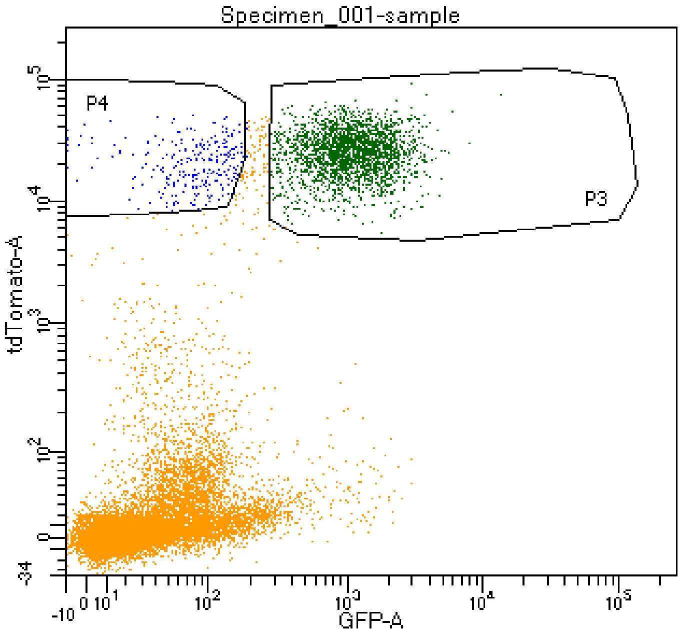

Tube: sample

Population	#Events	%Parent	%Total
All Events	30,000	####	100.0
P1	20,561	68.5	68.5
P2	19,888	96.7	66.3
P3	2,396	12.0	8.0
P4	274	1.4	0.9
