# Supplementary material for: RAGE is a key regulator of ductular reaction-mediated fibrosis during cholestasis
Source: EMBO Rep. 2025 Jan 2;26(3):880–907. doi: 10.1038/s44319-024-00356-7 (PMC11811172; doi:10.1038/s44319-024-00356-7)
Supplement: Supplementary file 4 — Source data Fig. 2 [file 44319_2024_356_MOESM4_ESM.zip › Figure 2/2E/Mouse IPA analysis report.pdf]

INGENUITY PATHWAY ANALYSIS

Analysis Name: Mouse_DE_contrast_genotype_RAGE_KO_vs_RAGE_control - 2020-01-24 12:50 PM
Analysis Creation Date: 2020-01-24
Build version: exported
Content version: 49932394 (Release Date: 2019-11-14)

Experiment Metadata

Name	Value

Analysis Settings

Reference set: Ingenuity Knowledge Base (Genes Only)

Relationship to include: Direct and Indirect

Includes Endogenous Chemicals

Optional Analyses: My Pathways My List

Filter Summary:

Consider only molecules and/or relationships where

(species = Mouse OR Rat OR Uncategorized OR Human) AND

(confidence = Experimentally Observed) AND

(tissues/cell lines = Amygdala OR Cardiomyocytes OR Cells not otherwise specified OR Peripheral blood monocytes OR WEHI-231 OR Effector memory helper T cells OR Peritoneal macrophages OR Activated helper T cells OR Activated Vd1 Gamma-delta T cells OR Other Breast Cancer Cell Lines OR CD56dim NK cells OR Pheochromocytoma cell lines not otherwise specified OR 3T3-L1 cells OR Other Neurons OR RKO OR

(c) 2000-2020 QIAGEN. All rights reserved.

OVCAR-3 OR Peripheral blood lymphocytes OR Prostate Gland OR Other Nervous System OR Lung Cancer Cell Lines not otherwise specified OR Spleen OR SW-620 OR Other Cells OR Activated CD56bright NK cells OR Mesenchymal stem cells OR Activated CD56dim NK cells OR Other CNS Cell Lines OR TK-10 OR Central memory cytotoxic T cells OR Smooth muscle cells not otherwise specified OR OVCAR-5 OR Kidney Cancer Cell Lines not otherwise specified OR Plasma cells OR Endothelial cells not otherwise specified OR Hematopoietic progenitor cells OR Other Ovarian Cancer Cell Lines OR Forestomach OR RAW 264.7 OR MDA-N OR Subventricular Zone OR HS 578T OR HCT-116 OR SF-295 OR Other Lymphoma Cell Lines OR NCI-H23 OR PANC-1 OR SW-480 OR Microvascular endothelial cells OR J-774A.1 OR Other Epithelial cells OR Other Melanoma Cell Lines OR Neutrophils OR Other Cell Line OR Central memory helper T cells OR Other Tissues and Primary Cells OR Other Smooth muscle cells OR SR OR Memory T lymphocytes not otherwise specified OR Vd2 Gamma-delta T cells OR Other Lung Cancer Cell Lines OR Striatum OR Stomach OR Thalamus OR Megakaryocytes OR SF-539 OR P19 OR Ovary OR Other Prostate Cancer Cell Lines OR Dendritic cells not otherwise specified OR RPMI-8266 OR Min6 OR Stem cells not otherwise specified OR Stromal cells OR Monocytes not otherwise specified OR Thymocytes OR SK-N-SH OR Esophagus OR Other Lymphocytes OR Keratinocytes OR Pre-B lymphocytes OR SK-MEL-28 OR Lymph node OR A2780 OR Other B lymphocytes OR T lymphocytes not otherwise specified OR NB4 OR A549-ATCC OR Granule cells OR Naive helper T cells OR Brainstem OR NK cells not otherwise specified OR Other Endothelial cells OR Kidney cell lines not otherwise specified OR Medulla Oblongata OR Other Monocyte-derived dendritic cells OR Cytotoxic T cells OR Beta islet cells OR Other T lymphocytes OR Eosinophils OR K-562 OR Olfactory Bulb OR Other Teratocarcinoma Cell Lines OR Nervous System not otherwise specified OR Skeletal Muscle OR Immune cell lines not otherwise specified OR Vd1 Gamma-delta T cells OR HeLa OR Cartilage Tissue OR Bone marrow cells not otherwise specified OR Sciatic Nerve OR MCF7 OR UACC-257 OR Lens OR White Matter OR KM-12 OR Ventricular Zone OR Liver OR Colon Cancer Cell Lines not otherwise specified OR INS-1 OR Granulosa cells OR Mononuclear leukocytes not otherwise specified OR Cos-7 cells OR Spinal Cord OR Myeloma Cell Lines not otherwise specified OR Activated Vd2 Gamma-delta T cells OR Hepatoma Cell Lines not otherwise specified OR U87MG OR Parietal Lobe OR Granule Cell Layer OR PC-12 cells OR T47-D OR Intraepithelial T lymphocytes OR Chondrocytes OR CCRF-CEM OR HepG2 OR NT2/D1 OR BDCA-3+ dendritic cells OR Tissues and Primary Cells not otherwise specified OR Cervical cancer cell line not otherwise specified OR Astrocytes OR Other Peripheral blood leukocytes OR Neurons not otherwise specified OR UACC-62 OR Th2 cells OR PC-3 OR CD56bright NK cells OR Other Bone marrow cells OR BA/F3 OR B lymphocytes not otherwise specified OR Uterus OR HOP-62 OR Salivary Gland OR MDA-MB-361 OR Fibroblast cell lines not otherwise specified OR Other Kidney Cancer Cell Lines OR Monocyte-derived dendritic cells not otherwise specified OR Peripheral blood leukocytes not otherwise specified OR Lymphoma Cell Lines not otherwise specified OR Neuroblastoma Cell Lines not otherwise specified OR Choroid Plexus OR NCI-H332M OR U2OS OR Melanocytes OR ACHN OR Teratocarcinoma Cell Lines not otherwise specified OR U266 OR Hippocampus OR Pro-B lymphocytes OR Mast cells OR Other Monocytes OR A498 OR Testis OR Gray Matter OR Blood platelets OR SNB-75 OR Other NK cells OR Cortical neurons OR Small Intestine OR Brain OR IGROV1 OR Other Cervical cancer cell line OR NCI-ADR-RES OR HCC-2998 OR Putamen OR NCI-H522 OR Adipose OR Bone marrow-

(c) 2000-2020 QIAGEN. All rights reserved.

derived dendritic cells OR HEL OR Heart OR Macrophages not otherwise specified OR Pancreatic Cancer Cell Lines not otherwise specified OR Dorsal Root Ganglion OR Skin OR Organ Systems not otherwise specified OR Other Granulocytes OR Other Kidney cell lines OR Smooth Muscle OR Other Osteosarcoma Cell Lines OR Bladder OR LNCaP cells OR MEF cells OR Immature monocyte-derived dendritic cells OR Monocyte-derived macrophage OR Ovarian Cancer Cell Lines not otherwise specified OR Calvaria OR Cell Line not otherwise specified OR Swiss 3T3 cells OR 293 cells OR HMC-1 OR Lung OR Other Fibroblast cell lines OR CD4+ T-lymphocytes OR MDA-MB-231 OR Crypt OR Lymphocytes not otherwise specified OR Mammary Gland OR Hepatocytes OR Other Macrophage Cancer Cell Lines OR BDCA-1+ dendritic cells OR MG-63 OR Caco2 cells OR Adrenal Gland OR Other Leukemia Cell Lines OR Memory B cells OR Splenocytes OR Kidney OR RXF-393 OR Th17 cells OR Embryonic stem cells OR Adipocytes OR 786-0 OR Trachea OR Hypothalamus OR HUVEC cells OR SK-MEL-5 OR Epithelial cells not otherwise specified OR Natural T-regulatory cells OR Oocytes OR UO-31 OR Cerebellum OR BT-474 OR THP-1 OR Epidermis OR Granulocytes not otherwise specified OR Sertoli cells OR COLO205 OR DU-145 OR Pituitary Gland OR A375 OR HCT-15 OR Myeloid dendritic cells OR Vascular smooth muscle cells OR H460 OR MOLT-4 OR CAKI-1 OR Melanoma Cell Lines not otherwise specified OR Other Pancreatic Cancer Cell Lines OR EKVX OR U937 OR Substantia Nigra OR RBL-2H3 OR Cerebral Ventricles OR Thyroid Gland OR HOP-92 OR HL-60 OR Langerhans cells OR Cerebral Cortex OR PBMCs OR U251 OR Effector memory RA+ cytotoxic T cells OR Other Neuroblastoma Cell Lines OR MALME-3M OR Purkinje cells OR SK-OV-3 OR SN12C OR Plasmacytoid dendritic cells OR Osteoblasts OR Other Myeloma Cell Lines OR Nucleus Accumbens OR Osteosarcoma Cell Lines not otherwise specified OR BT-549 OR Hep3B OR LOX IMVI OR Other Hepatoma Cell Lines OR Prostate Cancer Cell Lines not otherwise specified OR Other Immune cell lines OR Retina OR Breast Cancer Cell Lines not otherwise specified OR Immune cells not otherwise specified OR Other Memory T lymphocytes OR Other Dendritic cells OR Mature monocyte-derived dendritic cells OR HuH7 OR CD34+ cells OR Effector T cells OR Leukemia Cell Lines not otherwise specified OR Pancreas OR CNS Cell Lines not otherwise specified OR Murine NKT cells OR Th1 cells OR Macrophage Cancer Cell Lines not otherwise specified OR MDA-MB-468 OR MDA-MB-435 OR Large Intestine OR NIH/3T3 cells OR Other Macrophages OR Naive B cells OR OVCAR-8 OR J774 OR Dermis OR Trigeminal Ganglion OR Placenta OR Corpus Callosum OR OVCAR-4 OR Jurkat OR Caudate Nucleus OR Other Immune cells OR NCI-H226 OR SK-MEL-2 OR Other Organ Systems OR Pyramidal neurons OR Thymus OR Fibroblasts OR Cornea OR Other Stem cells OR Other Mononuclear leukocytes OR Bone marrow-derived macrophages OR Effector memory cytotoxic T cells OR HT29 OR M14 OR Microglia OR SF-268 OR Other Colon Cancer Cell Lines OR Other Pheochromocytoma cell lines) AND

(mol. types = biologic drug OR canonical pathway OR chemical - endogenous mammalian OR chemical - endogenous non-mammalian OR chemical - kinase inhibitor OR chemical - other OR chemical - protease inhibitor OR chemical drug OR chemical reagent OR chemical toxicant OR complex OR cytokine OR disease OR enzyme OR function OR G-protein coupled receptor OR group OR growth factor OR ion channel OR kinase OR ligand-dependent nuclear receptor OR mature microRNA OR microRNA OR other OR peptidase OR phosphatase OR transcription regulator OR translation regulator OR transmembrane receptor OR transporter) AND

(c) 2000-2020 QIAGEN. All rights reserved.

(data sources = An Open Access Database of Genome-wide Association Results OR BIND OR BioGRID OR Catalogue Of Somatic Mutations In Cancer (COSMIC) OR Chemical Carcinogenesis Research Information System (CCRIS) OR ClinicalTrials.gov OR ClinVar OR Cognia OR DIP OR DrugBank OR Gene Ontology (GO) OR GVK Biosciences OR Hazardous Substances Data Bank (HSDB) OR HumanCyc OR Ingenuity Expert Findings OR Ingenuity ExpertAssist Findings OR IntAct OR Interactome studies OR MIPS OR miRBase OR miRecords OR Mouse Genome Database (MGD) OR Obesity Gene Map Database OR Online Mendelian Inheritance in Man (OMIM) OR TarBase OR TargetScan Human)

Top Canonical Pathways

Name	p-value	Overlap
**Hepatic Fibrosis / Hepatic Stellate Cell Activation**	1.39E-07	9.1 % 17/186
**Caveolar-mediated Endocytosis Signaling**	1.14E-05	12.3 % 9/73
**Leukocyte Extravasation Signaling**	3.20E-05	7.1 % 14/197
**Hepatic Fibrosis Signaling Pathway**	3.71E-05	5.4 % 20/368
**Granulocyte Adhesion and Diapedesis**	4.87E-05	7.3 % 13/179

Top Upstream Regulators

Upstream Regulators

Name	p-value	Predicted Activation
**TNF**	7.27E-25	
**TGFB1**	1.40E-20	
**beta-estradiol**	6.75E-19	

(c) 2000-2020 QIAGEN. All rights reserved.

INGENUITY PATHWAY ANALYSIS

**dexamethasone**	5.12E-18
**IL1B**	1.26E-16

Causal Network

Name	p-value	Predicted Activation
**TRPS1**	9.32E-25	
**soraphen-A1alpha**	1.56E-23	
**Thrombospondin**	1.85E-23	
**aprotinin**	2.26E-23	
**sulpiride**	2.85E-23	

Top Diseases and Bio Functions

Diseases and Disorders

Name	p-value range	# Molecules
**Cancer**	4.80E-06 - 5.56E-15	408
**Organismal Injury and Abnormalities**	4.80E-06 - 5.56E-15	420
**Connective Tissue Disorders**	3.48E-06 - 1.81E-13	126
**Inflammatory Disease**	1.50E-06 - 1.81E-13	115
**Skeletal and Muscular Disorders**	2.72E-06 - 1.81E-13	107

Molecular and Cellular Functions

(c) 2000-2020 QIAGEN. All rights reserved.

Name	p-value range	# Molecules
Cellular Movement	4.37E-06 - 1.41E-22	168
Cell Death and Survival	4.80E-06 - 6.40E-17	184
Cell-To-Cell Signaling and Interaction	4.61E-06 - 1.76E-13	135
Cell Morphology	4.08E-06 - 1.14E-11	103
Cellular Development	4.08E-06 - 3.83E-11	179

Physiological System Development and Function

Name	p-value range	# Molecules
Cardiovascular System Development and Function	4.65E-06 - 1.28E-18	117
Organismal Development	2.95E-06 - 1.28E-18	196
Hematological System Development and Function	4.61E-06 - 1.14E-17	136
Tissue Morphology	4.50E-06 - 1.14E-17	154
Organismal Survival	3.52E-11 - 2.99E-15	153

Top Tox Functions

Assays: Clinical Chemistry and Hematology

Name	p-value range	# Molecules
Increased Levels of Potassium	1.85E-05 - 1.85E-05	5
Decreased Levels of Albumin	1.11E-01 - 5.34E-04	5

(c) 2000-2020 QIAGEN. All rights reserved.

INGENUITY PATHWAY ANALYSIS

**Increased Levels of Albumin**	6.69E-02 - 1.94E-02	2
**Increased Levels of Bilirubin**	3.83E-02 - 3.83E-02	1
**Increased Levels of Blood Urea Nitrogen**	6.69E-02 - 6.69E-02	2

Cardiotoxicity

Name	p-value range	# Molecules
**Cardiac Enlargement**	4.55E-01 - 2.18E-07	38
**Cardiac Dysfunction**	5.05E-01 - 2.07E-06	19
**Cardiac Fibrosis**	3.87E-01 - 1.26E-05	18
**Cardiac Dilation**	4.55E-01 - 5.26E-04	20
**Cardiac Congestive Cardiac Failure**	1.15E-03 - 1.15E-03	10

Hepatotoxicity

Name	p-value range	# Molecules
**Liver Hyperplasia/Hyperproliferation**	6.01E-01 - 4.97E-10	208
**Liver Necrosis/Cell Death**	9.31E-02 - 3.00E-07	22
**Liver Steatosis**	5.93E-01 - 3.09E-07	26
**Hepatocellular carcinoma**	6.01E-01 - 1.21E-06	51
**Liver Damage**	3.50E-01 - 3.02E-06	18

Nephrotoxicity

(c) 2000-2020 QIAGEN. All rights reserved.

INGENUITY PATHWAY ANALYSIS

Name	p-value range	# Molecules
**Renal Damage**	5.46E-01 - 1.85E-06	25
**Renal Tubule Injury**	5.14E-02 - 1.85E-06	17
**Glomerular Injury**	1.00E00 - 3.31E-04	28
**Renal Necrosis/Cell Death**	4.76E-01 - 6.99E-04	23
**Renal Inflammation**	1.00E00 - 3.53E-03	19

Top Regulator Effect Networks

Top Networks

ID	Associated Network Functions	Score
**1**	Developmental Disorder, Hereditary Disorder, Metabolic Disease	38
**2**	Carbohydrate Metabolism, Small Molecule Biochemistry, Drug Metabolism	36
**3**	Cellular Assembly and Organization, Cell Morphology, Cellular Movement	34
**4**	Small Molecule Biochemistry, Carbohydrate Metabolism, Lipid Metabolism	32

(c) 2000-2020 QIAGEN. All rights reserved.

**5**	Auditory Disease, Hereditary Disorder, Neurological Disease	32

Top Tox Lists

Name	p-value	Overlap
**Hepatic Fibrosis**	5.49E-11	15.2 % 17/112
**Liver Necrosis/Cell Death**	3.71E-07	6.8 % 22/322
**Cardiac Fibrosis**	1.72E-06	7.3 % 18/247
**Liver Proliferation**	2.04E-06	7.2 % 18/250
**Cardiac Hypertrophy**	4.41E-06	5.9 % 22/374

Top My Lists

Top My Pathways

Top Analysis-Ready Molecules

Molecules	Expr. Value	Chart
**RNPS1P1**	1.03E-158	
**Gm13443**	1.36E-26	
**TAGAP**	3.68E-26	
**RNPS1**	2.46E-25	

(c) 2000-2020 QIAGEN. All rights reserved.

**LTBP2**	1.86E-21
**ATP6V0C**	6.64E-21
**COL4A1**	7.13E-19
**Tmem181b-ps**	1.09E-17
**COL16A1**	2.13E-17
**KLF15**	7.29E-16

(c) 2000-2020 QIAGEN. All rights reserved.

INGENUITY PATHWAY ANALYSIS
