# Supplementary figures and images for: RAGE is a key regulator of ductular reaction-mediated fibrosis during cholestasis (part 2 of 2)
Source: EMBO Rep. 2025 Jan 2;26(3):880–907. doi: 10.1038/s44319-024-00356-7 (PMC11811172; doi:10.1038/s44319-024-00356-7)

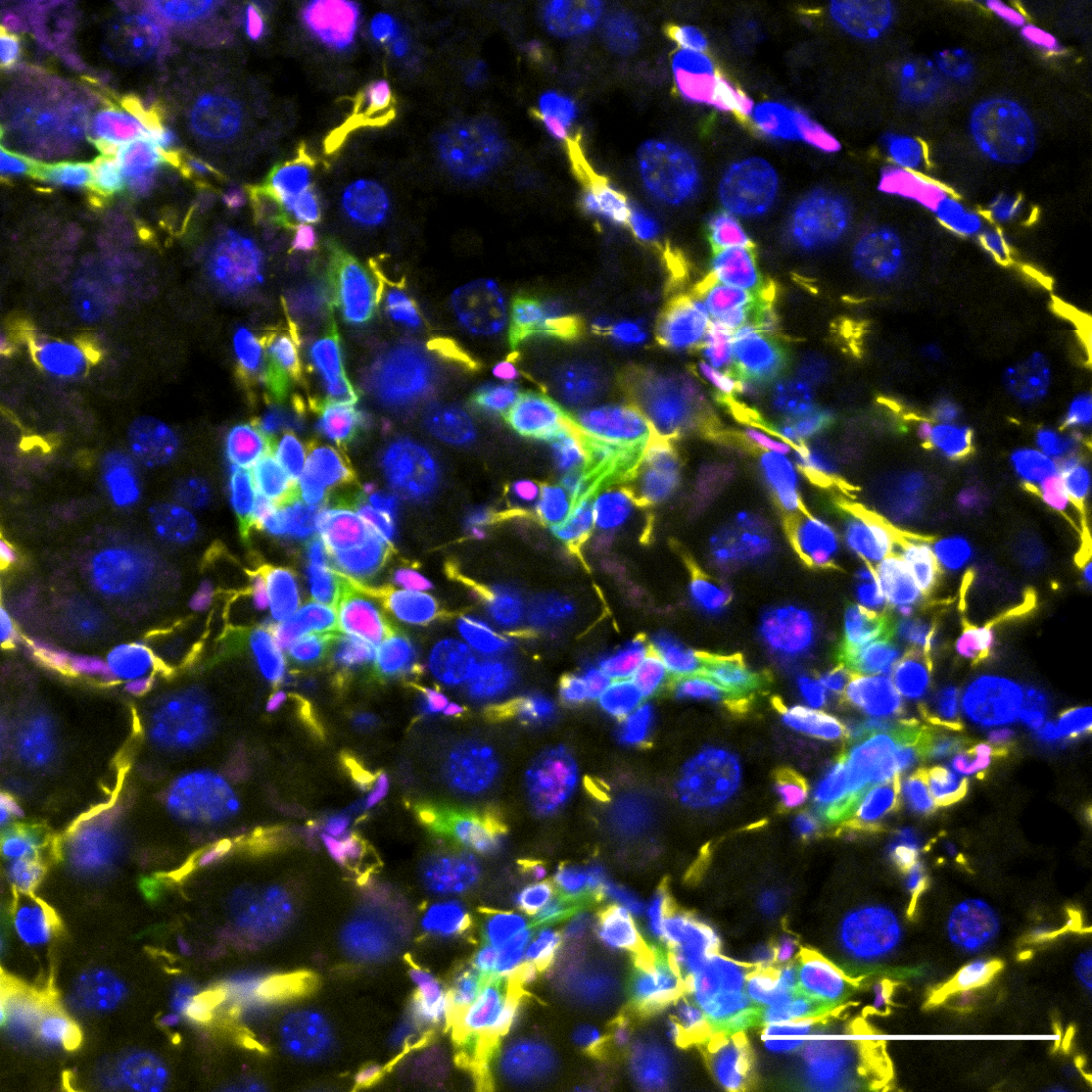

Supplement: Supplementary file 11 — Source data Fig. 9 [file 44319_2024_356_MOESM11_ESM.zip › Figure 9/4_CDE_WT_m152_19-Merge.tif]

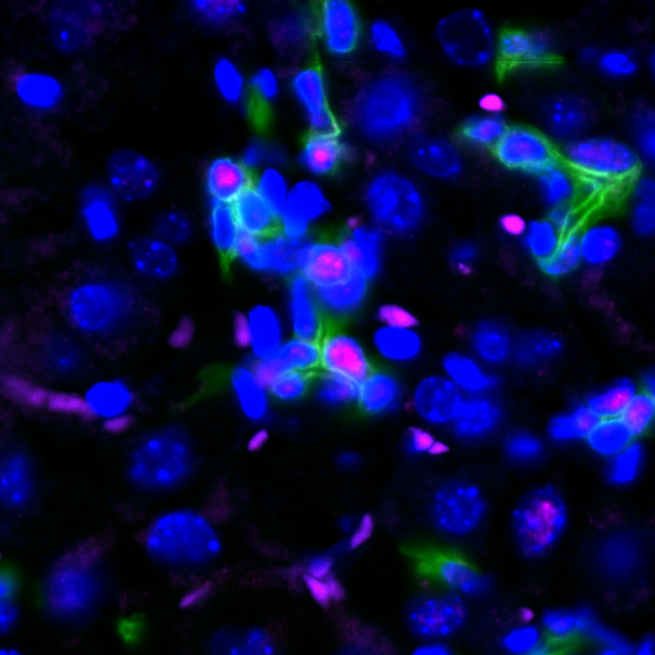

Supplement: Supplementary file 11 — Source data Fig. 9 [file 44319_2024_356_MOESM11_ESM.zip › Figure 9/4_CDE_WT_m152_19-Zoom in_HES1_CK19.tif]

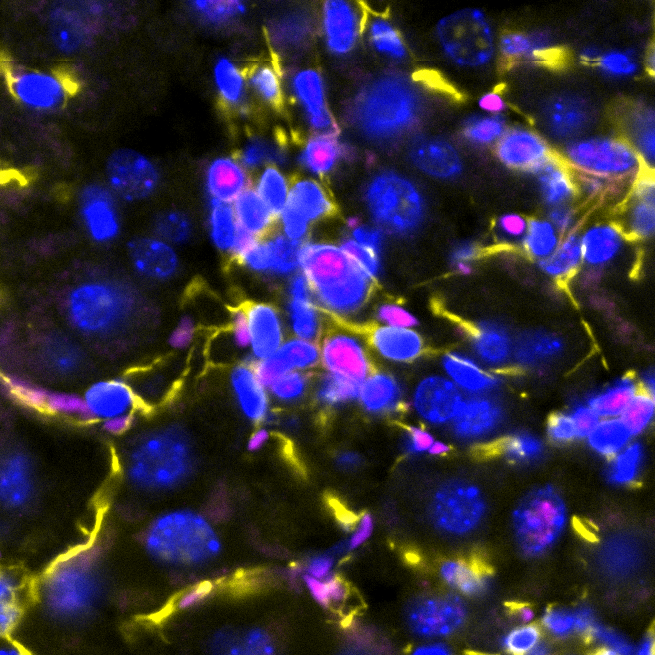

Supplement: Supplementary file 11 — Source data Fig. 9 [file 44319_2024_356_MOESM11_ESM.zip › Figure 9/4_CDE_WT_m152_19-Zoom in_HES1_Desmin.tif]

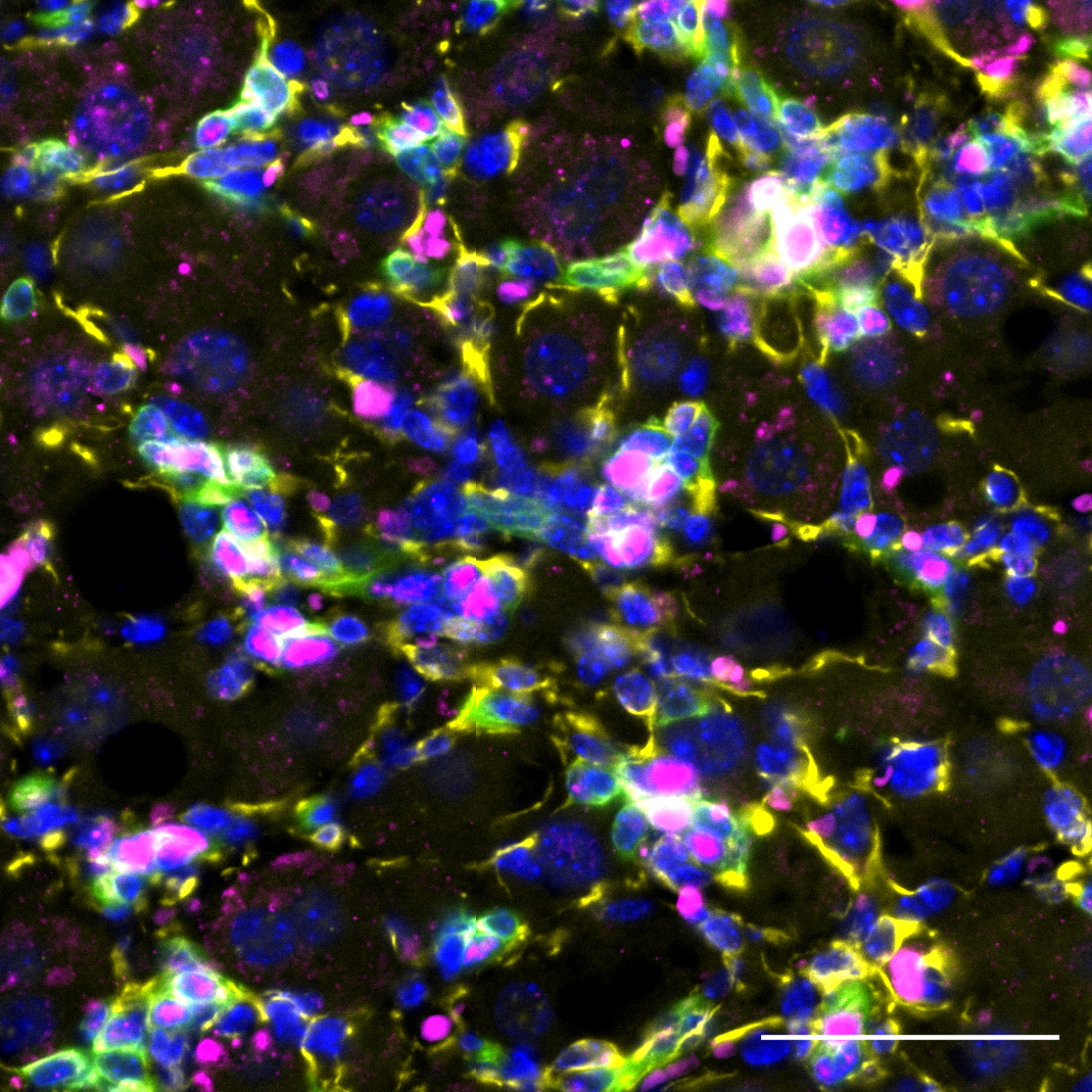

Supplement: Supplementary file 11 — Source data Fig. 9 [file 44319_2024_356_MOESM11_ESM.zip › Figure 9/5_CDE_Het_m414_19-Merge.tif]

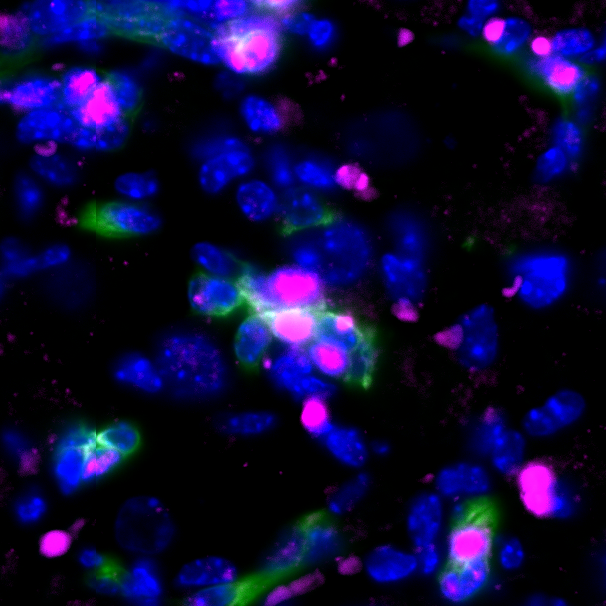

Supplement: Supplementary file 11 — Source data Fig. 9 [file 44319_2024_356_MOESM11_ESM.zip › Figure 9/5_CDE_Het_m414_19-Zoom in_HES1_CK19.tif]

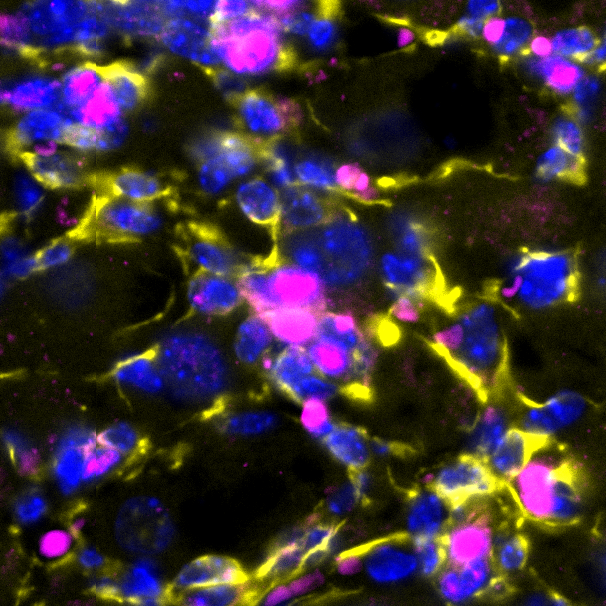

Supplement: Supplementary file 11 — Source data Fig. 9 [file 44319_2024_356_MOESM11_ESM.zip › Figure 9/5_CDE_Het_m414_19-Zoom in_HES1_Desmin.tif]

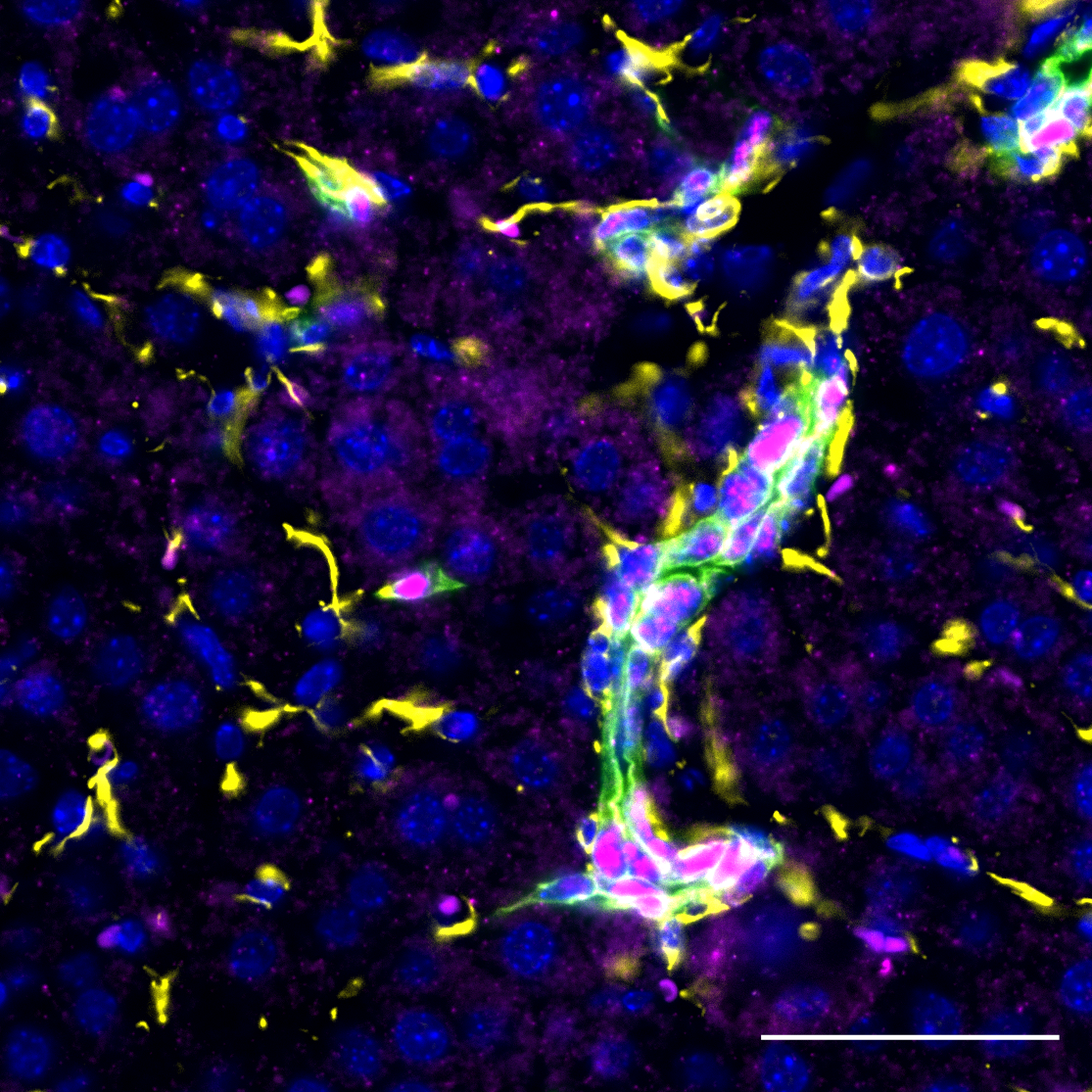

Supplement: Supplementary file 11 — Source data Fig. 9 [file 44319_2024_356_MOESM11_ESM.zip › Figure 9/6_CDE_KO_m418_19-Merge.tif]

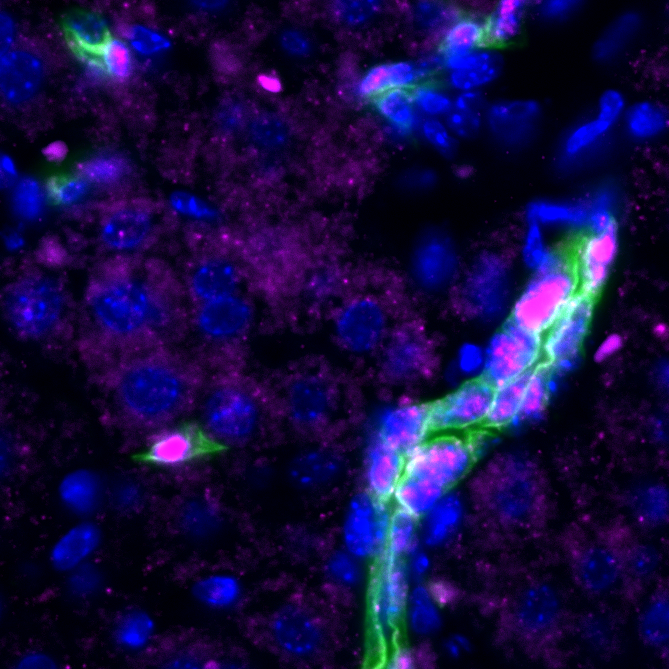

Supplement: Supplementary file 11 — Source data Fig. 9 [file 44319_2024_356_MOESM11_ESM.zip › Figure 9/6_CDE_KO_m418_19-Zoom in_HES1_CK19.tif]

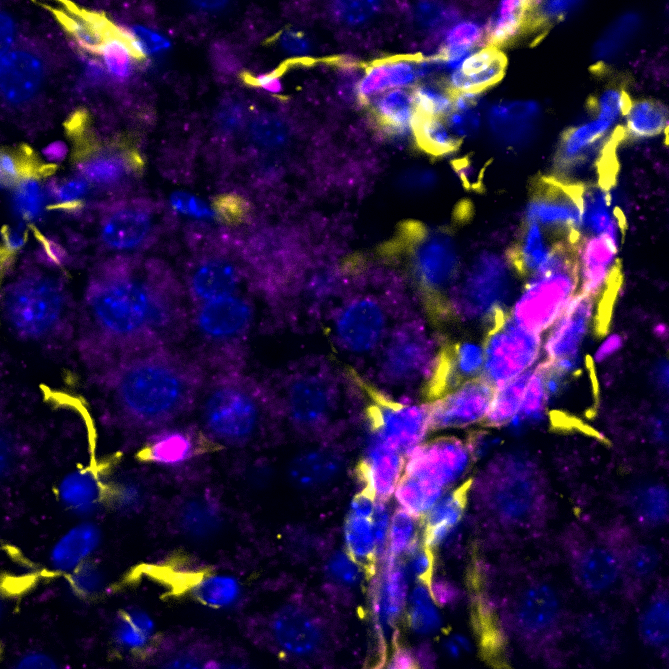

Supplement: Supplementary file 11 — Source data Fig. 9 [file 44319_2024_356_MOESM11_ESM.zip › Figure 9/6_CDE_KO_m418_19-Zoom in_HES1_Desmin.tif]
