# Supplementary material for: RAGE is a key regulator of ductular reaction-mediated fibrosis during cholestasis
Source: EMBO Rep. 2025 Jan 2;26(3):880–907. doi: 10.1038/s44319-024-00356-7 (PMC11811172; doi:10.1038/s44319-024-00356-7)
Supplement: Supplementary file 12 — Expanded View Figures [file 44319_2024_356_MOESM12_ESM.pdf]

Expanded View Figures

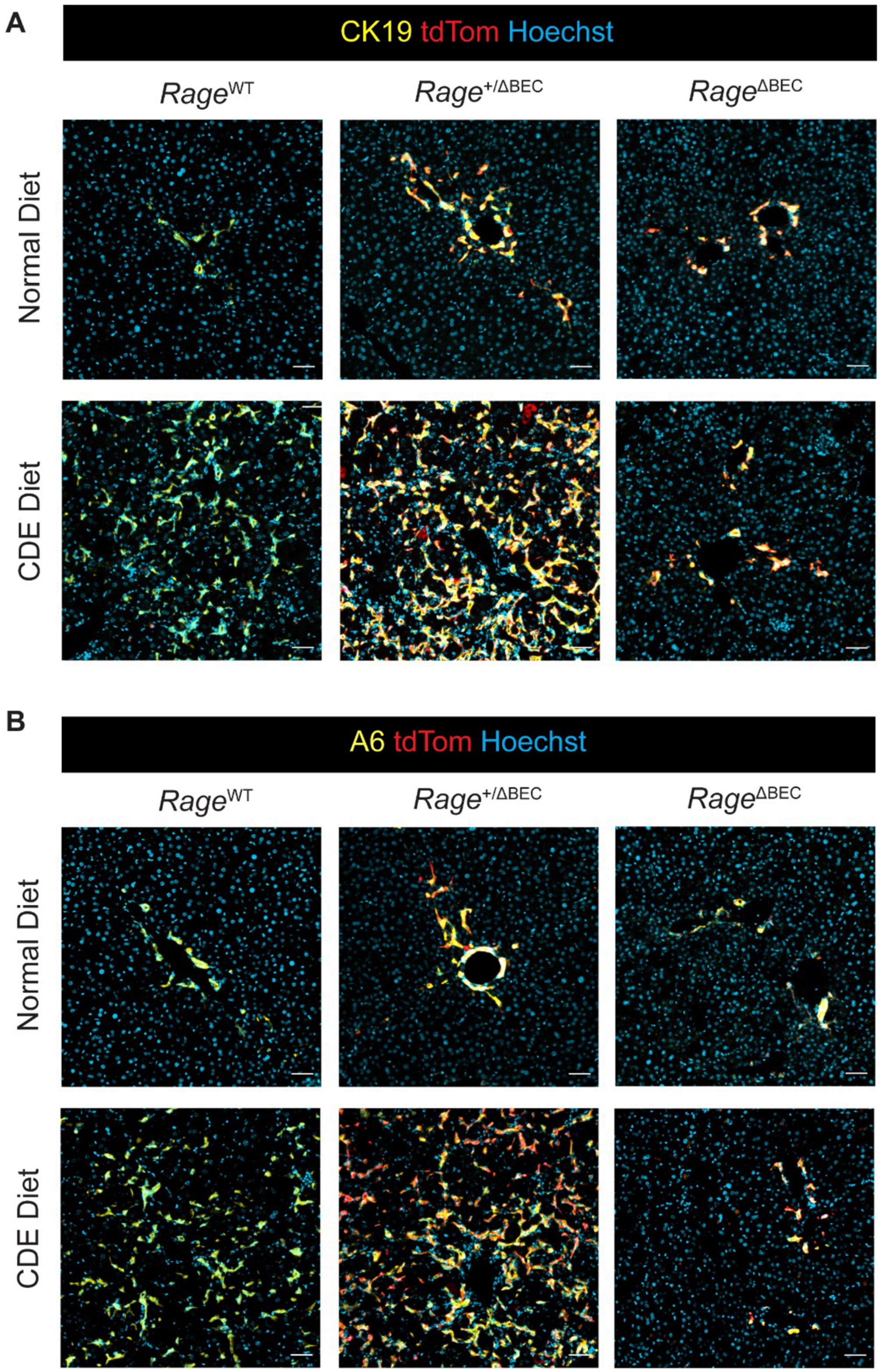

Figure EV1. Co-staining of biliary markers and tdTomato-labeled biliary cells.

(**A**) Representative images of IF of tdTomato and CK19. (**B**) Representative images of IF of tdTomato and A6. Scale bar = 50 μm. For normal diet (ND)-treated mice, *Rage*^{WT} ($n = 12$), *Rage*$^{+/\Delta BEC}$ ($n = 12$), *Rage*$^{\Delta BEC}$ ($n = 11$); for CDE-treated mice, *Rage*^{WT} ($n = 9$), *Rage*$^{+/\Delta BEC}$ ($n = 10$), *Rage*$^{\Delta BEC}$ ($n = 12$) (biological replicates).

 © The Author(s)

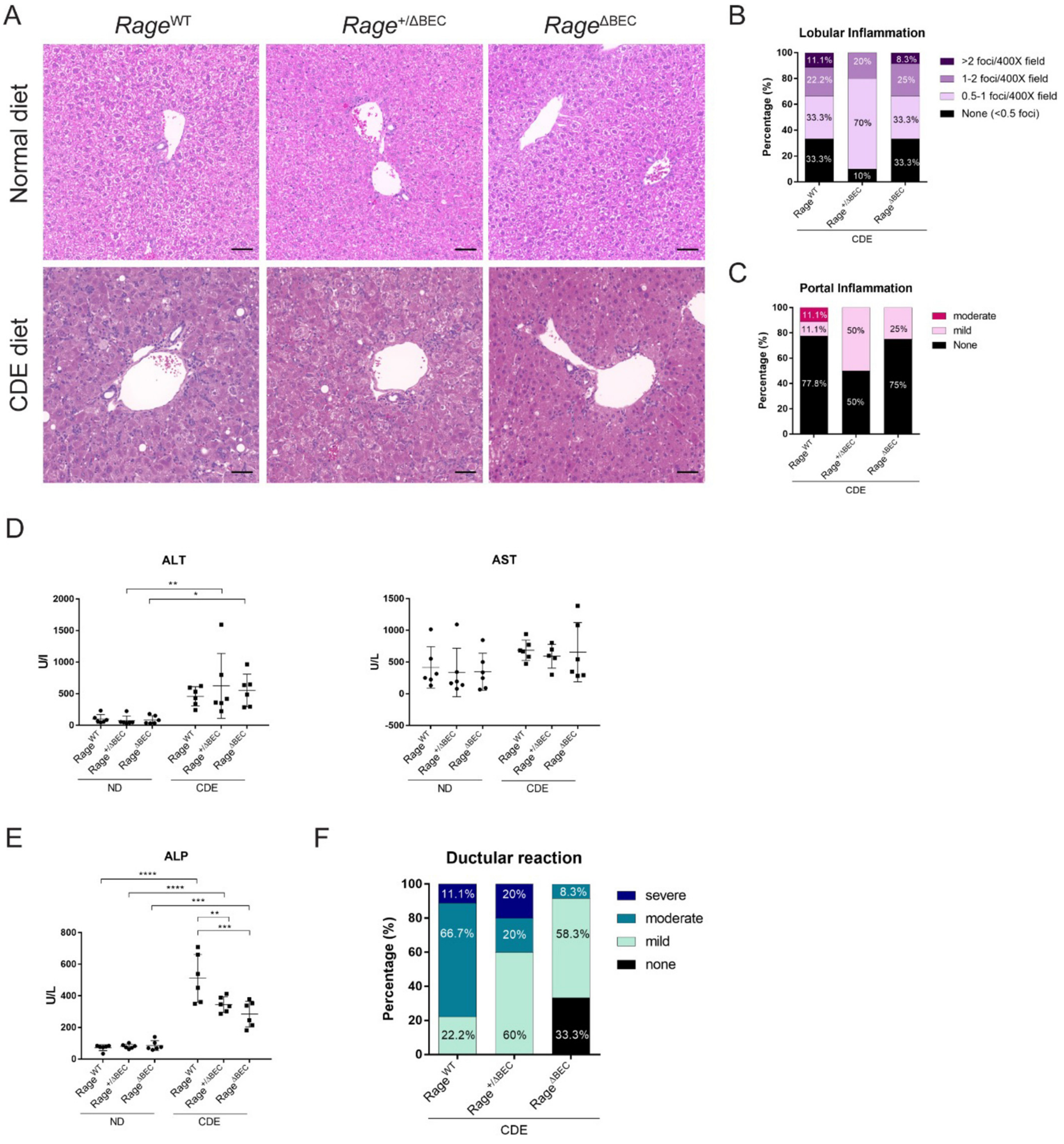

Figure EV2. *Rage* in BEC is not involved in inflammation during cholestatic injury.

(**A**) Hematoxylin & Eosin (H&E) staining of liver sections from *Rage*^{WT}, *Rage*$^{+/\Delta BEC}$ and *Rage*$^{\Delta BEC}$ mice fed with normal or CDE diet for 3 weeks. Scale bar = 50 μm. (**B**, **C**) Histopathological evaluation of lobular inflammation and portal inflammation based on H&E staining in CDE diet-challenged mice. (**D**) Biochemical serum analysis of hepatic damage markers, alanine aminotransferase (ALT) and aspartate aminotransferase (AST) and (**E**) the marker of cholestasis, alkaline phosphatase (ALP). Data was shown as mean ± s.d. of $n = 6$ biological replicates per group. Two-way ANOVA with Turkey's multiple comparisons test was used for statistical comparison (*$P < 0.05$, **$P < 0.01$, ***$P < 0.001$, ****$P < 0.0001$). (**F**) Histopathological evaluation of DR in *Rage*^{WT}, *Rage*$^{+/\Delta BEC}$ and *Rage*$^{\Delta BEC}$ mice fed with CDE diet for 3 weeks. For normal diet (ND)-treated mice, *Rage*^{WT} ($n = 12$), *Rage*$^{+/\Delta BEC}$ ($n = 12$), *Rage*$^{\Delta BEC}$ ($n = 11$); for CDE-treated mice, *Rage*^{WT} ($n = 9$), *Rage*$^{+/\Delta BEC}$ ($n = 10$), *Rage*$^{\Delta BEC}$ ($n = 12$).

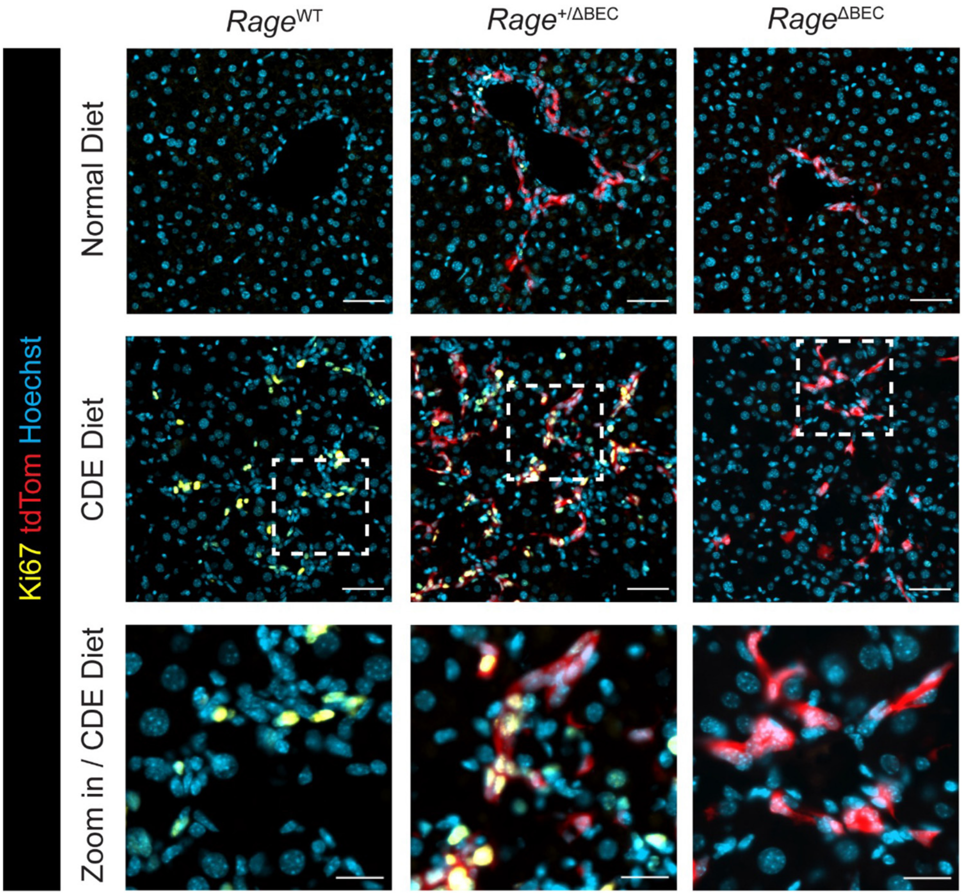

Figure EV3. ***Rage*** **in BECs may contribute to BEC proliferation during chronic injury.**

IF Staining of proliferation marker Ki67 on liver sections from $Rage^{WT}$, $Rage^{+/\Delta BEC}$ and $Rage^{\Delta BEC}$ mice fed with normal or CDE diet for 3 weeks. At least $n = 3$ animals (biological replicates) were evaluated per group. Scale bar = 50 µm for top and middle row. Scale bar = 20 µm for bottom row.

 © The Author(s)

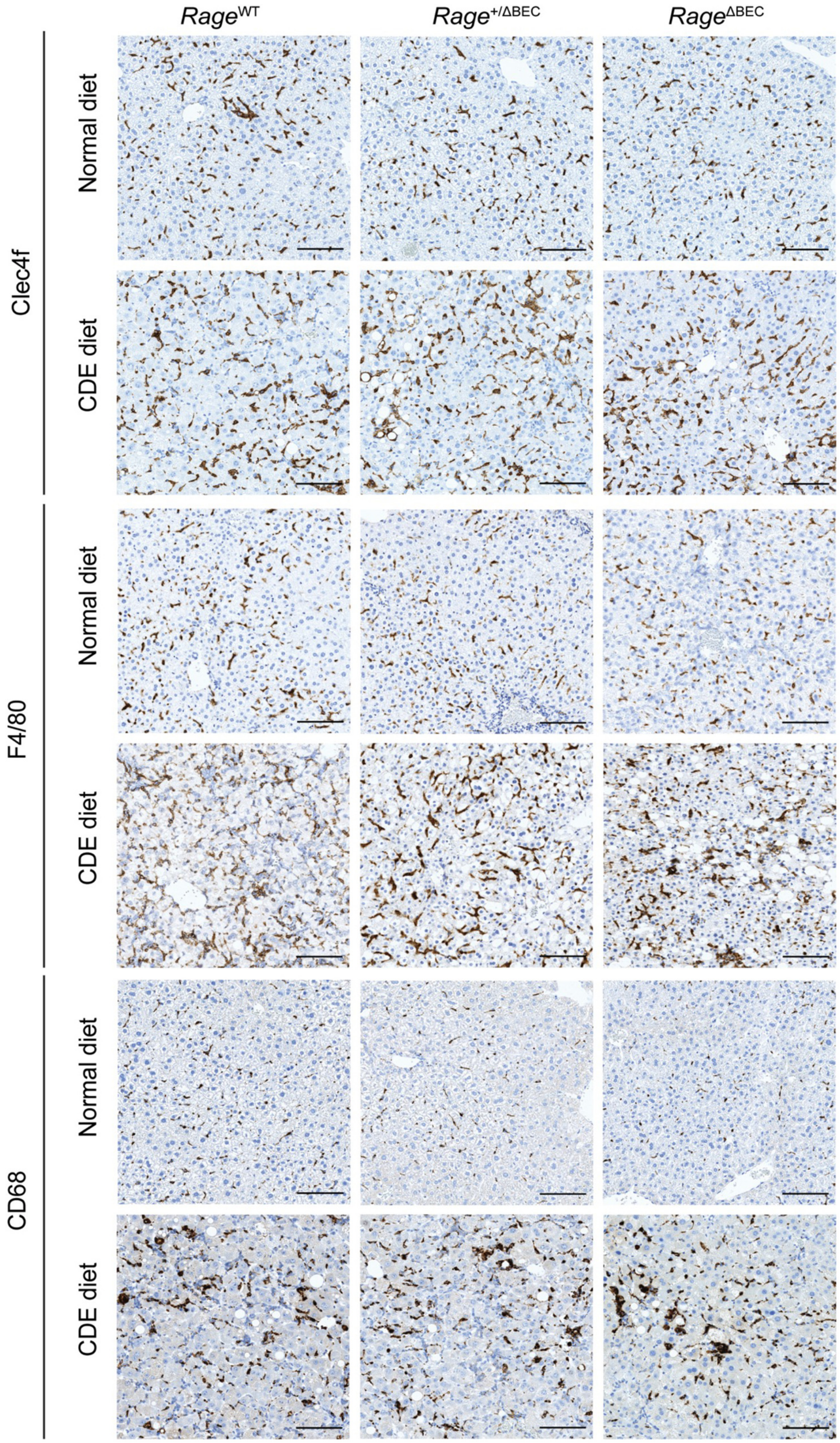

Figure EV4. ***Rage* in BECs does not contribute to immune cell infiltration during cholestatic injury.**

IHC staining of immune cells, including Clec4f for Kupffer cell, F4/80 for macrophages and CD68 for monocytes in *Rage*^{WT}, *Rage*$^{+/\Delta BEC}$ and *Rage*$^{\Delta BEC}$ mice fed with normal or CDE diet for 3 weeks. At least $n = 3$ animals (biological replicates) per group were evaluated. Scale bar = 100 µm.

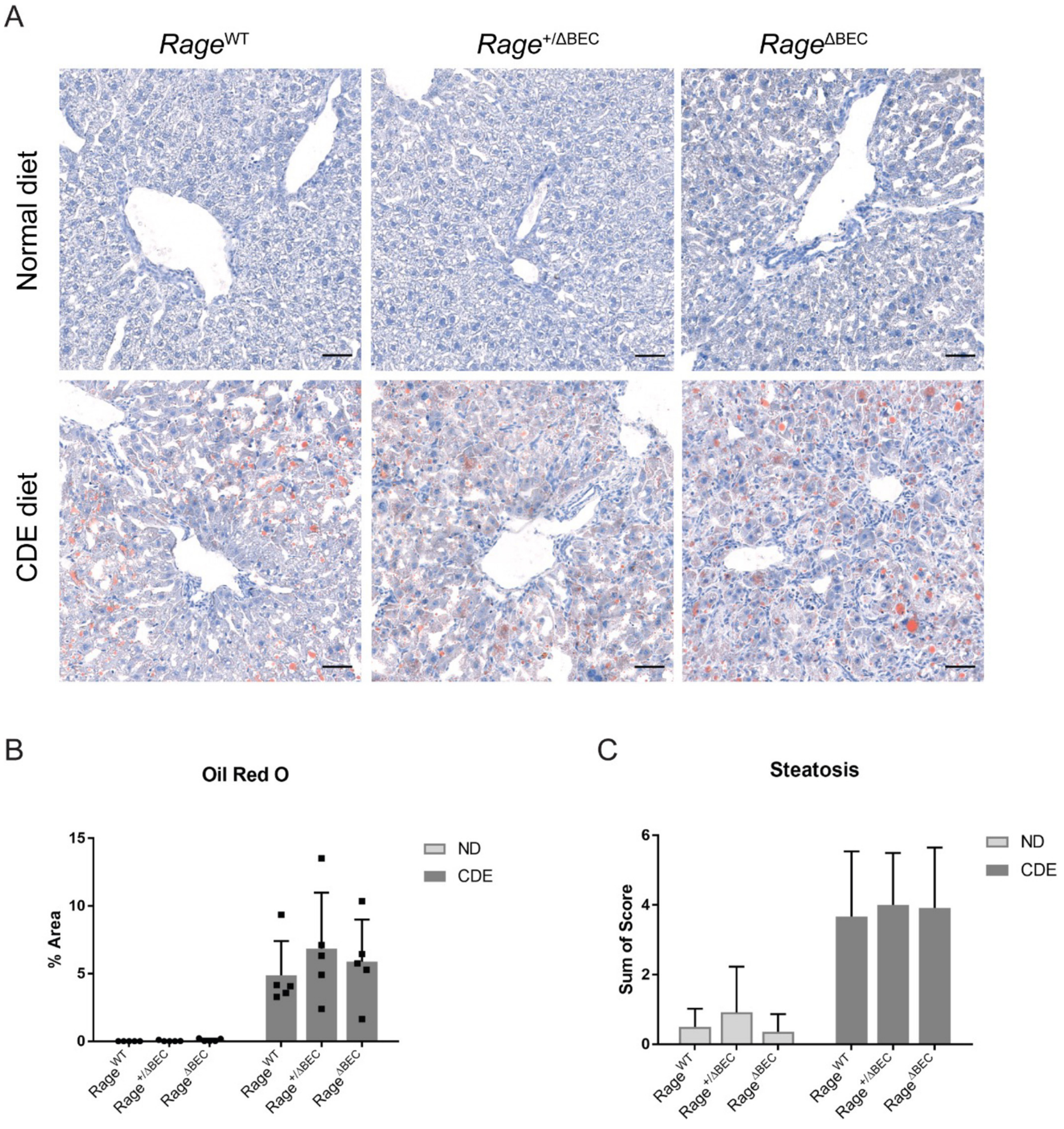

Figure EV5. *Rage* in BEC is not associated with steatosis upon chronic liver injury.

Rage^{WT}, *Rage*$^{+/\Delta BEC}$ and *Rage*$^{\Delta BEC}$ mice were fed with normal or CDE diet for 3 weeks. (**A**) Representative images of Oil Red O staining (scale bar = 50 μm) and (**B**) corresponding Oil Red O quantification. Data was shown as mean ± s.d. of $n = 5$ animals (biological replicates) per group. Two-way ANOVA with Turkey's multiple comparisons test was used for statistical comparison. (**C**) Histopathological evaluation of steatosis (based on H&E staining in Fig. EV2A). Two-way ANOVA with Turkey's multiple comparisons test was used for statistical comparison.

 © The Author(s)
